# Metathesis chemistry of inorganic cumulenes driven by B–O bond formation†

**DOI:** 10.1039/d4sc07487d

**Published:** 2024-12-18

**Authors:** Jianqin Tang, Chenyang Hu, Agamemnon E. Crumpton, Liam P. Griffin, Jose M. Goicoechea, Simon Aldridge

**Affiliations:** a Inorganic Chemistry Laboratory, Department of Chemistry, University of Oxford South Parks Road Oxford OX1 3QR UK simon.aldridge@chem.ox.ac.uk; b Department of Chemistry, Indiana University 800 E. Kirkwood Ave Bloomington IN 47405 USA jgoicoec@iu.edu

## Abstract

The reaction chemistry of an unprecedented ‘inorganic cumulene’ – featuring a five-atom BNBNB chain – towards C=O (and related) multiple bonds is disclosed. In marked contrast to related all-carbon systems, the intrinsic polarity of the BNBNB chain (featuring electron-rich nitrogen and electron-deficient boron centres) enables metathesis chemistry with electrophilic heteroallenes such as CO_2_ and with organic carbonyl compounds. Transfer of the borylimide unit to [CO], [CS], [PP{(NDippCH_2_)_2_}] and [C(H)Ph] moieties generates (boryl)N=C=X systems (X = O, S, PP{(NDippCH_2_)_2_}, C(H)Ph), driven thermodynamically by B–O bond formation. Pairwise exchange of O and {(HCDippN)_2_}BN fragments occurs *via* consecutive [2+2] cyclo-addition/cyclo-reversion steps. An isolable complex of stoichiometry K[(boryl)NB(O)OC(H)Ph], formed *via* [2+2] cycloaddition to [(boryl)N=B=O]^−^ can be shown to be an intermediate in the formation of (boryl)N=C(H)Ph, and provides corroborating evidence for a DFT-calculated mechanism proceeding *via* a ‘bora-Wittig’ mechanism.

## Abstract 2

The reaction chemistry of an unprecedented ‘inorganic cumulene’ – featuring a five-atom BNBNB chain – towards C=O (and related) multiple bonds is disclosed.

## Graphical abstract

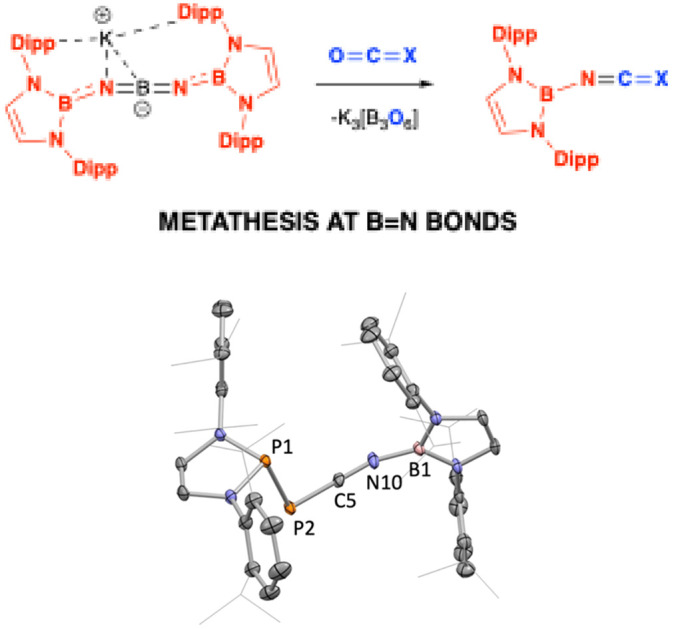

## Introduction

Metathesis constitutes a powerful and widely used methodology in synthetic chemistry, with applications spanning organometallics, pharmaceuticals, fine chemicals and materials science.^1^ While metathesis reactions utilising C=C bonds predominate, the possibility of exploiting C=O bonds in a similar fashion offers a strategy, for example, for the transformation of CO_2_ into useful chemical products.^2^ The use of oxophilic early transition metal complexes to accomplish C=O to C=NR transformations has been extensively reported,^3,4^ and similar chemistry using main group metal/metalloid derivatives has begun to be explored, driven, for example, by the formation of strong Al–O or Si–O bonds.^5,6^ Given the similar thermodynamic driver associated with B–O bond formation (559 kJ mol^−1^, *cf.* 595 kJ mol^−1^ for Al–O),^7^ we wondered whether boron-based non-metal systems could be employed to transform C=O into C=N bonds.^8,9^

**Scheme 1:**
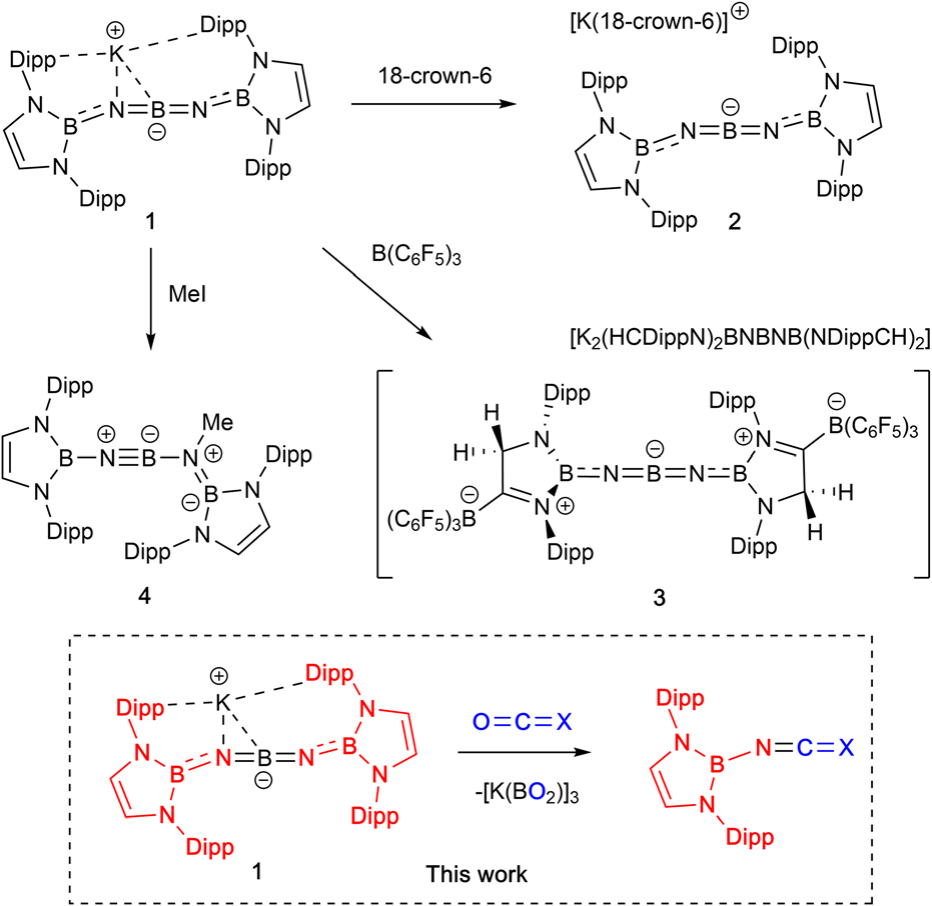
Preliminary studies of ‘inorganic cumulene’ 1.^10^

We have recently developed high-yielding synthetic routes to ‘inorganic cumulenes’ (Scheme 1).^10,11^ These five-atom BNBNB chains analogous to [4]cumulenes can possess either linear or bent structures (*i.e.*1/2 or 3) depending on the nature of the groups attached to the boron termini. Chemical modification of diamido-terminated 1/2 to generate a linear system best described by a B=N=B=N=B resonance structure involves assimilation of the strong Lewis acid B(C_6_F_5_)_3_ to enhance the π-acidity of the end groups and conjugate the N-lone pairs. In addition, preliminary studies of the reactivity of K[(HCDippN)_2_BNBNB(NDippCH)_2_] (1) verify experimentally the intrinsic polarity within the BNBNB chain. The reaction of 1 with methyl iodide, for example, proceeds *via* nucleophilic attack by nitrogen to generate an iminoborane (4), featuring a formal BN triple bond.

The central NBN core of 1 can also be viewed as being a diimidoboryl anion isoelectronic with CO_2_ itself, which prompted us to probe whether it might be capable of metathesis chemistry with O-containing heteroallenes, leading to exchange of borylimide/O atom fragments, driven thermodynamically by the formation of BO bond(s). We perceived that the previously demonstrated N-centred nucleophilicity of 1 might enable facile reactivity with substrates (such as CO_2_ itself) which react primarily as electrophiles.^10^

## Results and discussion

**Scheme 2:**
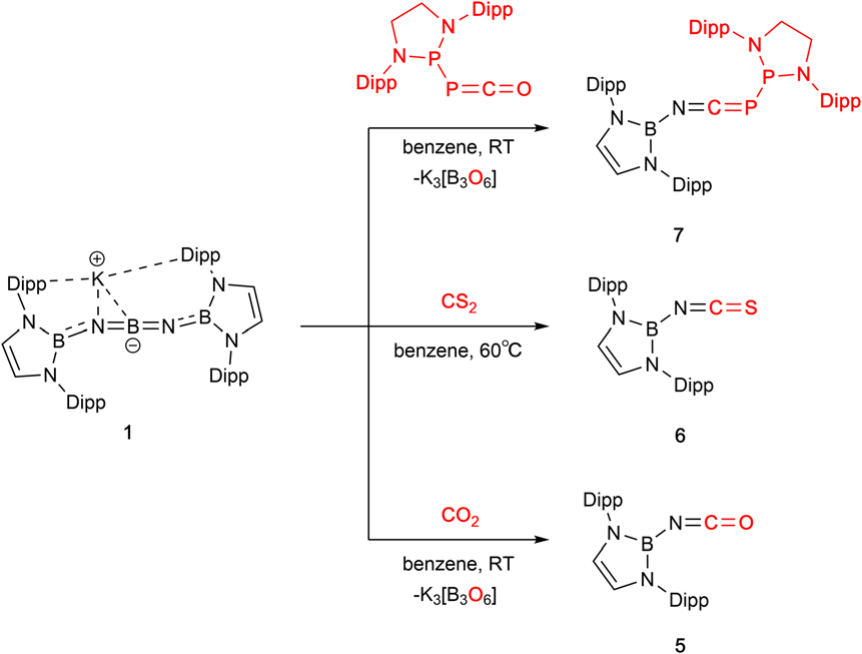
Metathesis reactions of compound 1 with CO_2_, CS_2_ and {(H_2_CDippN)_2_}PPCO.

**Fig. 1:**
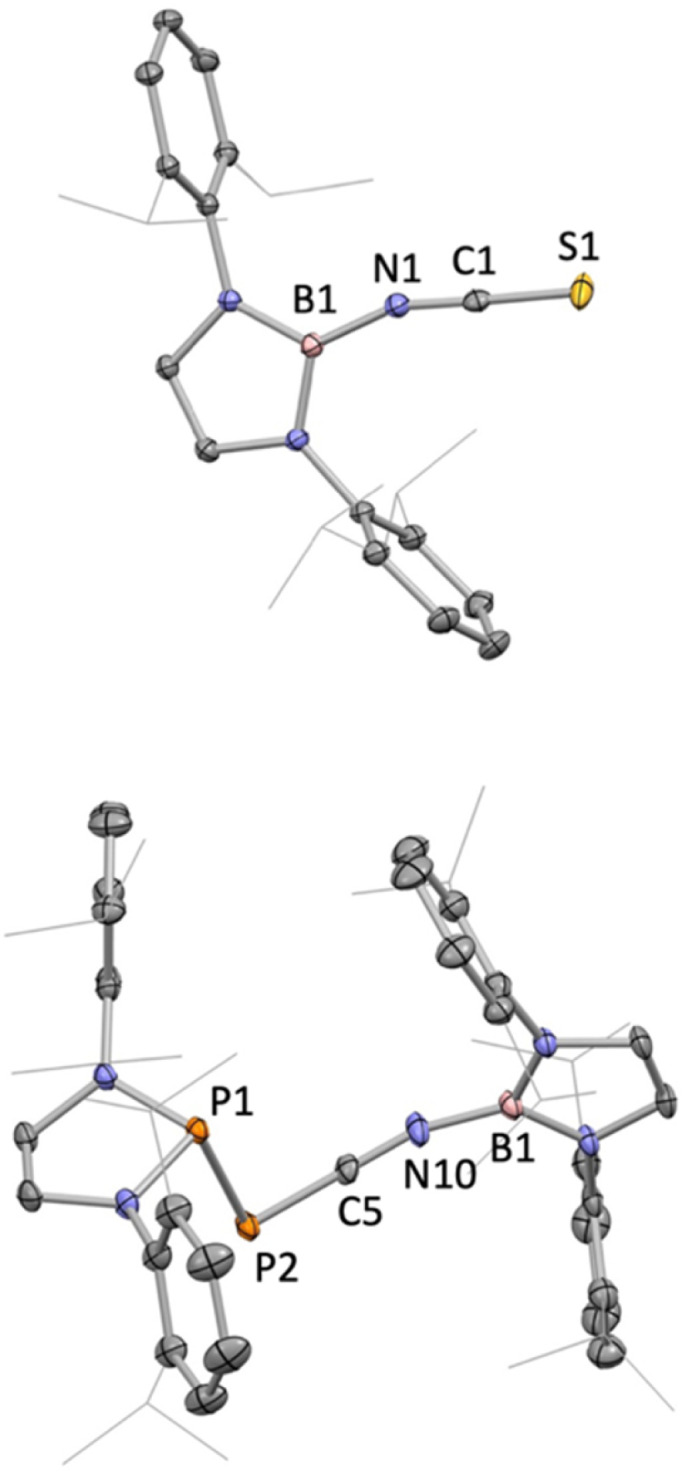
Molecular structures of 6 (upper) and one of the two independent molecules in the asymmetric unit of 7 (lower) in the solid state as determined by X-ray crystallography. Second disorder component and all hydrogen atoms omitted, and ^i^Pr groups shown in wireframe format for clarity; thermal ellipsoids shown at the 35% probability level. Key bond lengths (Å) and angles (°): (for 6) B1–N1 1.447(1), N1–C1 1.179(7), C1–S1 1.584(7), B1–N1–C1 156.8(4), N1–C1–S1 175.3(6); (for 7) B1–N10 1.430(3), N10–C5 1.184(3), C5–P2 1.668(2), P2–P1 2.299(1), B1–N10–C5 162.8(2), N10–C5–P2 178.7(2), C5–P2–P1 88.7(1).

**Fig. 2:**
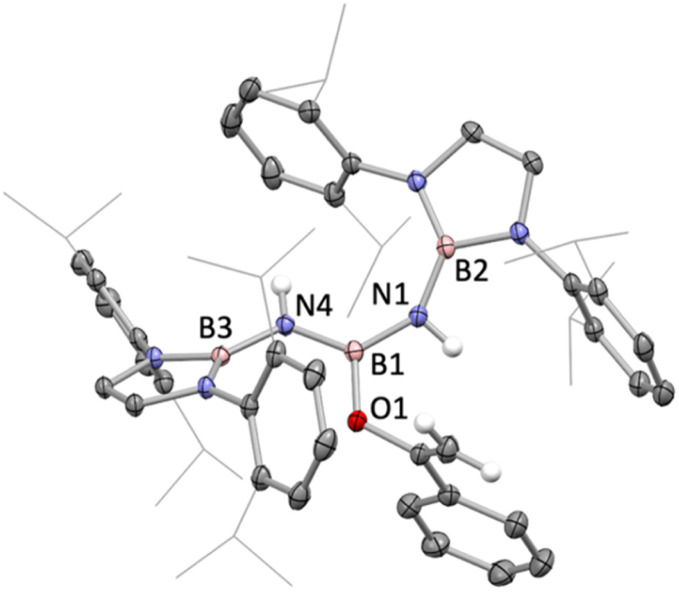
Molecular structure of 8 in the solid state as determined by X-ray crystallography. Most hydrogen atoms omitted and ^i^Pr groups shown in wireframe format for clarity; thermal ellipsoids shown at the 35% probability level. Key bond lengths (Å): B2–N1 1.438(2), N1–B1 1.426(2), B1–N4 1.420(1), N4–B3 1.451(1), B1–O1 1.398(1).

The reactions of 1 with CO_2_, CS_2_ or the phosphaphosphinidene carbonyl {(H_2_CDippN)_2_}PPCO proceed *via* O/N(boryl) meta-thesis, leading to the formation of the iminoboryl compounds (boryl)NCE (E = O (5), S (6) and PP{(NDippCH_2_)_2_} (7); Scheme 2). 5 is a known compound, having recently been reported by Yuvaraj and Jones to be formed by N(boryl) transfer to CO_2_ from a boryl-substituted silaimine at 80 °C (in *ca.* 40% yield).^5*c*^ In the chemistry reported here, 5 is formed in excellent yield (*ca.* 90%, based on the availability of two N(boryl) units per molecule of 1), while the heavier C=E bonds in 6 (E = S) and 7 (E = P) are constructed in more modest yields (*ca.* 50% in each case). Both 6 and 7 are novel compounds, and each has been characterized by standard spectroscopic/analytical techniques and by X-ray crystallography (Fig. 2). The ^11^B NMR resonances determined for 6 and 7 (*δ*_B_ = 16.1 and 16.0 ppm) are similar to that measured for 5 (*δ*_B_ = 17.9 ppm), while the ^31^P signals measured for 7 (*δ*_P_ = −183.3 ppm, PP̲=C; 165.6 ppm, P̲P=C; ^1^*J*_P,P_ = 298.0 Hz) are shifted from those reported for the precursor {(H_2_CDippN)_2_}PPCO (*δ*_P_ = −245.6, 167.9 ppm; ^1^*J*_P,P_ = 252.1 Hz)^12^ to an extent that is markedly greater for the exocyclic phosphorus atom. Structurally, 5–7 are also similar, (Fig. 1) featuring comparable N=C distances (1.170(2), 1.179(7) and 1.184(3) Å), and a B–N–C angle which is marginally wider in the case of 7 (162.8(2)° *vs.* 159.4(1) and 156.8(4)° for 5 and 6, respectively).^5*c*^

The identity of the co-product derived from the central boron atom is difficult to establish unequivocally, although the formation of a white precipitate suggests that (in the cases of 5 and 7 at least) it is possibly the well-known cyclic metaborate K_3_[B_3_O_6_] (*i.e.* the trimeric aggregate of K[BO_2_]), consistent with its insolubility in organic solvents.^13^

**Scheme 3:**
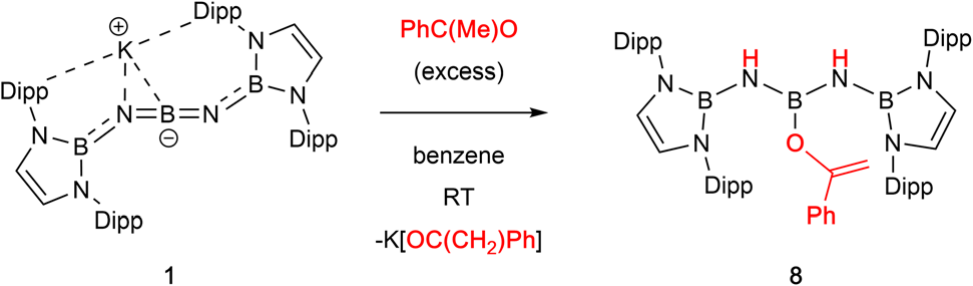
Reaction of 1 with acetophenone to generate boron enolate 8.

With a view to exploring further both the scope and mechanism of this metathesis chemistry, we investigated the reactivity of 1 with organic carbonyl compounds. Ketones featuring enolizable hydrogen atoms, however, react with 1 in a different manner, reflecting the basic nature of the N centres, leading to α-deprotonation and assimilation of the corresponding enolate fragment at the central boron atom. Thus, the reaction of 1 with (excess) acetophenone leads to protonation of both nitrogen atoms, and the formation of a B-bound OC(Ph)CH_2_ moiety (Scheme 3 and Fig. 2).

**Fig. 3:**
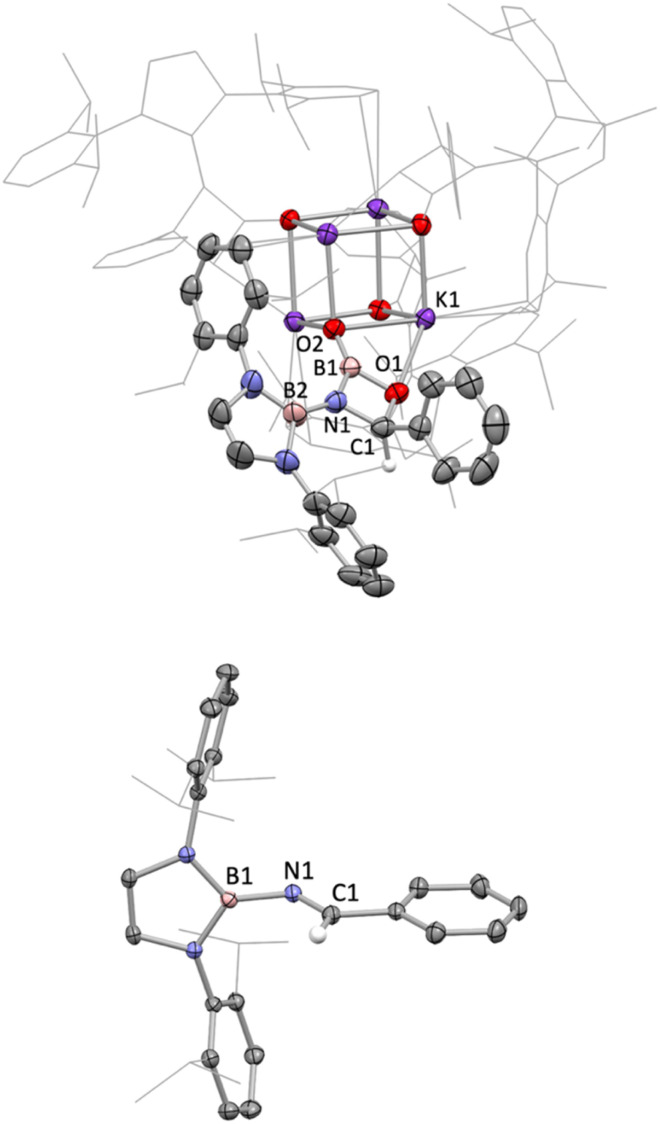
Molecular structures of compounds 9 (upper) and 10 (lower) in the solid state as determined by X-ray crystallography. Thermal ellipsoids are drawn at 50% probability level. Most H atoms are omitted for clarity. Key bond lengths (Å) and angles (°): (for 6) B2–N1 1.423(5), N1–C1 1.470(5), C1–O1 1.441(4), O1–B1 1.453(4), B1–N1 1.478(5), B1–O2 1.283(4); (for 7) B1–N1 1.448(3), N1–C1 1.254(3), B1–N1–C1 126.1(2), N1–C1–C2 123.5(2).

**Scheme 4:**
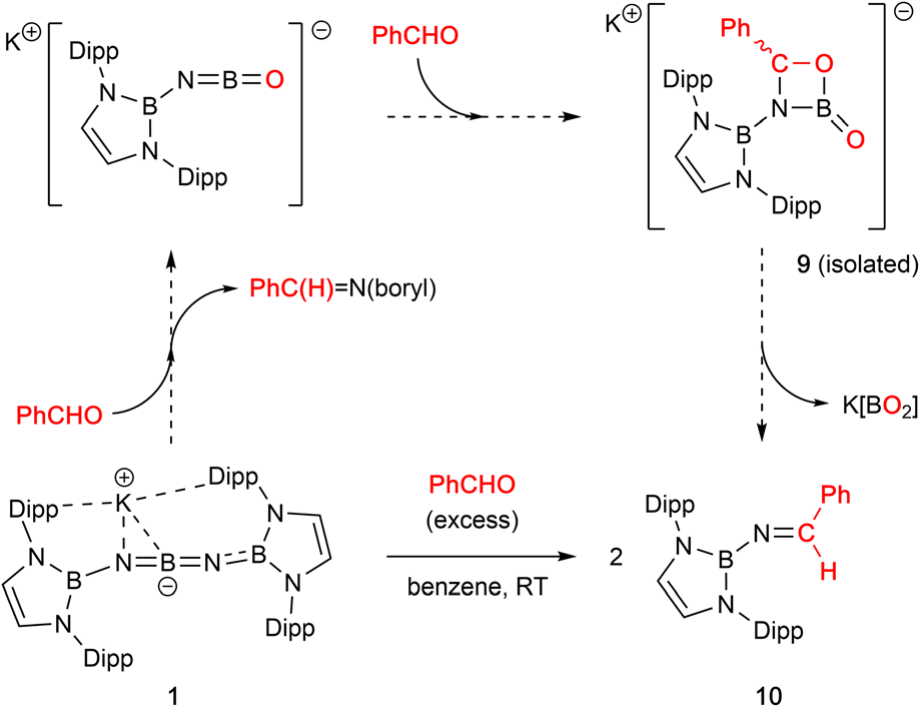
Cycloaddition reactivity of 1 with benzaldehyde, PhCHO, leading to the formation of borylimine 10.

Non-enolizable carbonyls such as benzaldehyde, PhCHO, on the other hand, react *via* C=O bond cleavage in a manner similar to CO_2_ and {(H_2_CDippN)_2_}PPCO, and (in addition) allow further mechanistic details to be uncovered. Addition of excess benzaldehyde to a solution of 1 in benzene, leads to the clean formation (over a period of *ca.* 12 h at room temperature) of the borylimine, *trans*-(boryl)NC(H)Ph (10), which has been characterized structurally by X-ray crystallography (Fig. 3), and by standard spectroscopic/analytical methods. If the reaction is monitored *in situ* by ^1^H NMR spectroscopy in benzene-d_6_ solution, an intermediate species can be identified after 30 min (Fig. S10†), which is characterised by Dipp methine resonances at 3.61/3.89 ppm (each 2H), a boryl backbone signal (also 2H) at 6.05 ppm and a singlet (1H) at 5.28 ppm (*cf. δ*_H_ = 8.65 ppm for the imine proton of the final product 10). If volatiles are removed *in vacuo* after 1 h, and the reaction mixture recrystallized from pentane, a mixture of crystals is obtained, which includes not only 10, but also an intermediate K[(boryl)NB(O)OC(H)Ph], 9, which precedes the formation of the final imine product *via* elimination of the K[BO_2_] fragment (Scheme 4 and Fig. 3).

Analysis of crystalline 9 by X-ray diffraction reveals that the solid material contains a tetrameric potassium salt (based on a cubic K_4_O_4_ core), and an organic fragment featuring a four-membered CBNO ring. [2+2] cyclo-reversion *via* CO and BN bond cleavage then offers a route for the onward conversion of this species to borylimine 10.

Structurally, the organic component of 9 can be viewed as being derived from cycloaddition of the C=O double bond of benzaldehyde across the N=B double bond of an intermediate species of the type [(boryl)N=B=O]^−^. Geometrically, the bond length associated with the B–O linkage within the four-membered ring (1.453(4) Å) is consistent with a single bond; the associated O atom participates in a weak contact with a single K^+^ counterion, *d*(K⋯O) = 2.710(3) Å. By contrast, the exocyclic B–O bond is much shorter (1.283(4) Å), consistent with appreciable π-bonding character,^14^ and the associated O atom bridges three K^+^ cations (*d*(K⋯O) = 2.596(2), 2.604(2), 2.778(2) Å).

**Fig. 4:**
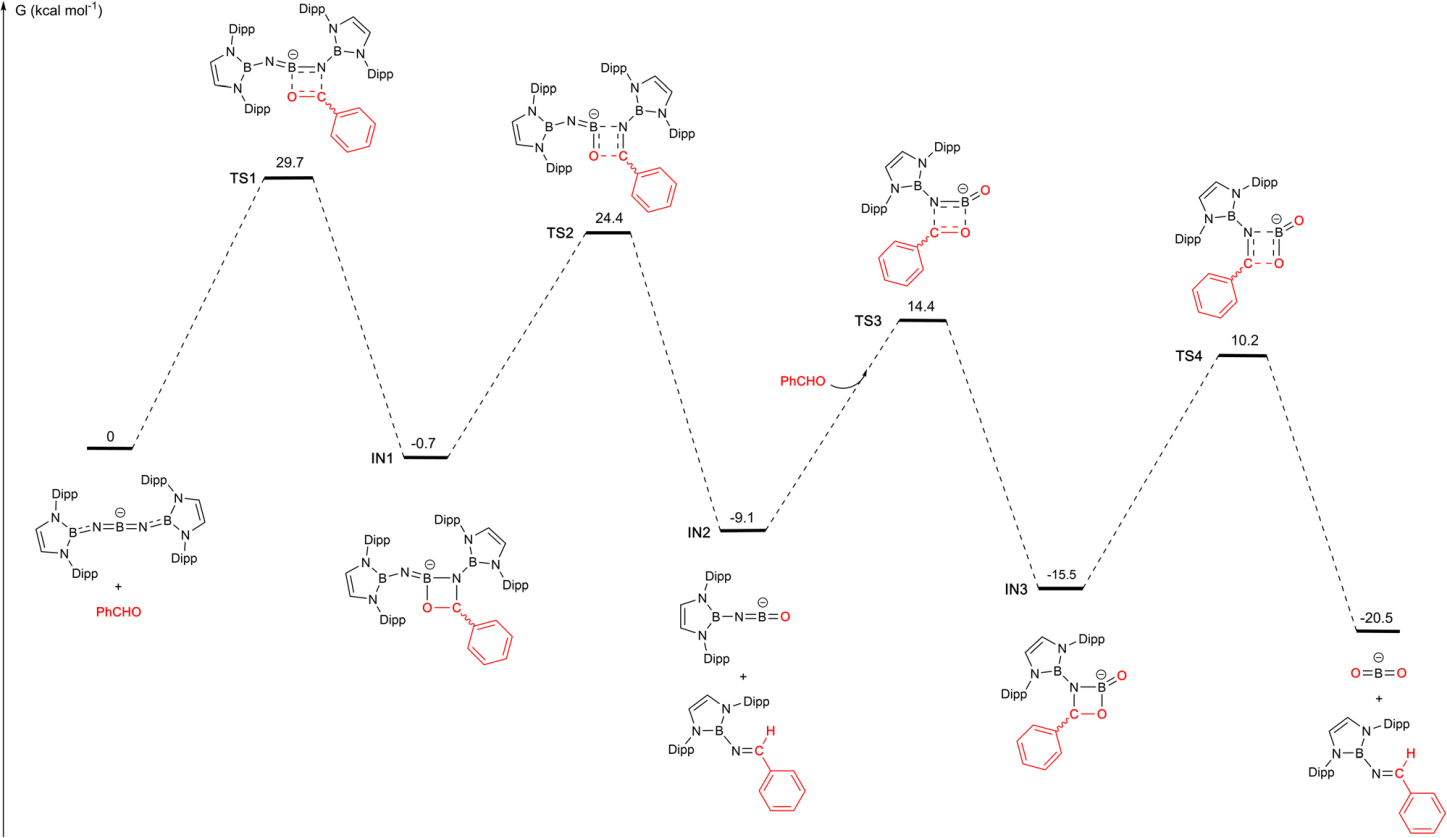
DFT calculated mechanism for the reaction of PhCHO with the anionic component of 1 (calculated at the M06-2X/Def2-SVP//Def2-TZVP level of theory with solvation modelled with smd, benzene; K^+^ counterions omitted for simplicity).^15^

These structural and reactivity data suggest that 1 acts as a transfer agent for the [(boryl)N] group *via* a Wittig-type reaction involving [2+2] cyclo-addition/cyclo-reversion steps. To further probe the mechanism of the reaction with benzaldehyde, we examined the potential energy surface by density functional theory calculations (M06-2X/Def2-SVP//Def2-TZVP). The overall process involves two [2+2] cyclo-addition/reversion steps, each involving one of the B=N bonds associated with the central boron atom of 1 (Fig. 4). The overall process is exergonic (by −20.5 kcal mol^−1^), and successive steps occur *via* transition states which are *ca.* 25–30 kcal mol^−1^ above the preceding intermediate. The first step in the reaction pathway is thought to be rate-determining, and involves [2+2] cyclo-addition of the C=O bond of PhCHO across one B=N unit of the anionic component of 1 to give IN1, which features an COBN four-membered ring bearing a pendant iminoboryl function. This step resembles the [2+2] cyclo-addition reported by Jones in the room temperature reaction of CO_2_ with the Si=N bond of a silaimine.^5*c*^ In the case of 1 it is only slightly exergonic (−0.7 kcal mol^−1^) and occurs *via* a barrier of +29.7 kcal mol^−1^.

Subsequent cyclo-reversion to release a first equivalent of *trans*-(boryl)NC(H)Ph, and generate IN2, [(boryl)N=B=O]^−^, involves traversing a slightly lower activation barrier of 25.1 kcal mol^−1^, and provides an extra 8.4 kcal mol^−1^ of thermodynamic stabilization. The uptake of a second equivalent of PhCHO occurs *via* a similar transition state to first generate a third intermediate IN3, akin to the structurally characterized system 9. [2+2] cyclo-reversion then yields the observed second equivalent of *trans*-(boryl)NC(H)Ph. The last two steps incur activation barriers of 23.5 and 25.7 kcal mol^−1^, respectively, and lead to a process which (overall) is exergonic to the tune of −20.5 kcal mol^−1^.^15^ We postulate, given the relatively low transition state barriers calculated beyond IN1, that the crystallization of compound 9 from solution relates to the crystallinity/insolubility of the tetrameric potassium salt, rather than to the intrinsic reaction barrier associated with its onward conversion to the final product (which is relatively low).

Finally, we set out to probe experimentally whether the differing geometric and electronic structures of bent BNBNB anion 1 and its linear counterpart 3 might lead to differences in reactivity towards electrophilic substrates. Surprisingly, however, the reaction of 3 with MeI can be shown to generate the same iminoborane product (HCDippN)_2_BNBN(Me)B(NDippCH)_2_ (4) as is formed in the reactions of 1/2 under comparable conditions (Scheme 1). The accompanying release of B(C_6_F_5_)_3_ can be confirmed by *in situ*^11^B and ^19^F NMR measurements (Fig. S13 and S14†). In similar fashion, the uptake of CO_2_ by 3 also yields boryl-isocyanate 5 in essentially quantitative conversion (Fig. S15†). These observations imply that the assimilation of the borane by the diazaborolyl heterocycle of 1/2 is chemically reversible – at least under these reactivity conditions.

## Experimental

Included here are synthetic and characterising data for compounds 6, 7, 8 and 10. Data for all compounds, representative ^1^H NMR spectra, and details of crystallographic and computational studies are included in the ESI.†

(HCDippN)_2_BNCS, 6. To a solution of 1 (100 mg 0.117 mmol) in benzene (10 mL) was added a drop of CS_2_ (*ca.* 0.02 mL, excess). The solution was heated at 60 °C overnight with a colour change from orange to red. Volatiles were removed *in vacuo* and the residue extracted with pentane (10 mL). The resulting solution was concentrated to *ca.* 2 mL and colourless crystals of 6 were obtained upon standing at room temperature which were suitable for X-ray crystallography. These were isolated by filtration and washed with cold pentane (2 × 1 mL). Yield: 51.3 mg (52.0%). ^1^H NMR (500 MHz, C_6_D_6_, 298 K): *δ*_H_ 1.17 (d, ^3^*J*_H,H_ = 6.8 Hz, 12H, CH_3_ of Dipp), 1.29 (d, ^3^*J*_H,H_ = 6.8 Hz, 12H, CH_3_ of Dipp), 3.12 (sept, ^3^*J*_H,H_ = 6.8 Hz, 4H,CH̲(CH_3_)_2_ of Dipp), 5.91 (s, 2H, CH of boryl), 7.13 (m, 4H, *m*-ArH of Dipp), 7.20 (m, 2H, *p*-ArH of Dipp). ^11^B{^1^H} NMR (160 MHz, C_6_D_6_, 298 K): *δ*_B_ 16.1 (boryl). ^13^C{^1^H} NMR (126 MHz, C_6_D_6_, 298 K): *δ*_C_ 24.0, 24.5 (CH_3_ of Dipp), 28.9 (C̲H(CH_3_)_2_ of Dipp), 118.6 (CH of boryl), 123.9 (*m*-Ar of Dipp), 128.6 (*p*-Ar of Dipp), 136.4 (CN of Dipp), 146.3 (*o*-Ar of Dipp), not observed (N=C=S). Elemental microanalysis: calc. for C_27_H_36_BN_3_S (%): C 72.80, H 8.15, N 9.43; meas. C 72.76, H 8.26, N 9.17.

(HCDippN)_2_BNCPP(NDippCH_2_)_2_, 7. To a mixture of 1 (80 mg, 0.094 mmol) and (CH_2_DippN)_2_PPCO (69.3 mg, 0.148 mmol) in a 25 mL Schlenk flask was added 5 mL benzene. The resulting solution was stirred for 12 h with a colour change to dark green. Volatiles were removed *in vacuo* and the residue extracted with pentane (3 mL). The resulting solution was concentrated to *ca.* 1 mL and colourless crystals of 7 were obtained upon standing at room temperature which were suitable for X-ray crystallography. These were isolated by filtration and washed with cold pentane (2 × 1 mL). Yield: 88.6 mg (55.2%). ^1^H NMR (500 MHz, C_6_D_6_, 298 K): *δ*_H_ 1.05 (d, ^3^*J*_H,H_ = 7.0 Hz, 12H, CH_3_ of (boryl)Dipp), 1.13 (d, ^3^*J*_H,H_ = 7.0 Hz, 12H, CH_3_ of (boryl)Dipp), 1.19, 1.25, (d, ^3^*J*_H,H_ = 6.6 Hz, 12H, CH_3_ of (phosphanyl)Dipp),1.31 (dd, ^3^*J*_H,H_ = 6.6 Hz, 12H, CH_3_ of (phosphanyl)Dipp), 3.03(sept, ^3^*J*_H,H_ = 7.0 Hz, 4H,CH̲(CH_3_)_2_ of (boryl)Dipp), 3.11, 3.58 (m, 4H, CH_2_ of phosphanyl), 3.48, 3.76 (m, 4H,CH̲(CH_3_)_2_ of (phosphanyl)Dipp), 5.86 (s, 2H, CH of boryl), 7.04 (m, 4H, *m*-ArH of (boryl)Dipp), 7.10 (m, 6H, ArH of (phosphanyl)Dipp), 7.18 (m, 2H, *p*-ArH of (boryl)Dipp). ^11^B{^1^H} NMR (160 MHz, C_6_D_6_, 298 K): *δ*_B_ 16.0 (boryl). ^31^P NMR (162 MHz, C_6_D_6_, 298 K): *δ*_P_ −183.3 (d, PP̲=C, ^1^*J*_P,P_ = 298.0 Hz),165.6 (d, P̲P=C, ^1^*J*_P,P_ = 298.0 Hz). ^13^C{^1^H} NMR (126 MHz, C_6_D_6_, 298 K): *δ*_C_ 23.9, 24.4 (CH_3_ of Dipp), 24.7, 25.2, 25.9 (d, CH_3_ of (phosphanyl)Dipp), 28.8 (C̲H(CH_3_)_2_ of (boryl)Dipp), 28.9, 29.5 (d, C̲H(CH_3_)_2_ of (phosphanyl)Dipp), 54.9 (d, CH_2_ of phosphanyl), 118.5 (CH of boryl), 123.6 (*m*-Ar of (boryl)Dipp), 123.9 (*m*-Ar of (phosphanyl)Dipp), 124.9 (*p*-Ar of (phosphanyl)Dipp), 127.5 (*o*-Ar of (boryl)Dipp), 136.8 (CN of (boryl)Dipp), 137.8 (d, CN of (phosphanyl)Dipp), 146.2 (*o*-Ar of (boryl)Dipp), 148.7, 150.4 (*o*-Ar of (phosphanyl)Dipp), not observed (N=C=P).

[{(HCDippN)_2_BNH}_2_B]OC(CH_2_)Ph, 8. 1 (15 mg 0.018 mmol) was dissolved in benzene (4 mL) and a drop of acetophenone (0.01 mL, excess) added. The reaction mixture was stirred for 1 h with an accompanying colour change to light yellow. Volatiles were removed *in vacuo* and the residue extracted with pentane (3 mL). The resulting solution was concentrated to *ca.* 0.5 mL and yellow crystals of 8 were obtained upon standing at room temperature which were suitable for X-ray crystallography. These were isolated by filtration and washed with cold pentane (2 × 1 mL). Yield: 7.8 mg (47.0%). ^1^H NMR (500 MHz, C_6_D_6_, 298 K): *δ*_H_ 1.16 (d, ^3^*J*_H,H_ = 6.6 Hz, 24H, CH_3_ of Dipp), 1.18 (d, ^3^*J*_H,H_ = 6.6 Hz, 24H, CH_3_ of Dipp), 2.56 (br, 2H, NH), 3.12, 4.43 (s, each 1H, C=CH_2_), 3.28 (sept, ^3^*J*_H,H_ = 6.8 Hz, 8H,CH̲(CH_3_)_2_ of Dipp), 5.97 (s, 2H, CH of boryl), 7.07 (m, 17H, ArH of Dipp). ^11^B{^1^H} NMR (160 MHz, C_6_D_6_, 298 K): *δ*_B_ 22.4 (boryl). ^13^C{^1^H} NMR (126 MHz, C_6_D_6_, 298 K): *δ*_C_ 23.8, 25.5 (CH_3_ of Dipp), 28.8 (C̲H(CH_3_)_2_ of Dipp), 97.7 (C=C̲H_2_), 118.6 (CH of boryl), 124.2 (*m*-Ar of Dipp), 125.3, 128.8, 136.7 (Ph), 127.4 (*p*-Ar of Dipp), 139.6 (CN of Dipp), 146.3 (*o*-Ar of Dipp), 154.4 (C̲=CH_2_). Elemental microanalysis: calc. for C_60_H_81_B_3_N_6_O (%): C 77.09 H 8.73 N 8.99; meas. C 76.89 H 8.51 N 9.12.

(HCDippN)_2_BN[κ^2^-(C,B)-C(H)PhBO_2_] 9 and (HCDippN)_2_BNC(H)Ph, 10. 1 (100 mg 0.117 mmol) was dissolved in benzene (10 mL) and a drop of benzaldehyde (0.02 mL, excess) added. The resulting solution was stirred for 30 min, with an accompanying colour change to yellow. Volatiles were removed *in vacuo*, and the residue extracted with pentane (5 mL). The resulting solution was concentrated to *ca.* 0.5 mL and a mixture of colourless crystals of 9 (unstable in solvent and briefly visible in NMR) and 10 were obtained which were suitable for X-ray crystallography.

(HCDippN)_2_BNCHPh, 10. 1 (100 mg 0.117 mmol) was dissolved in benzene (10 mL) and a drop of benzaldehyde (0.02 mL, excess) added. The resulting solution was stirred for 12 h with a colour change to light yellow. Volatiles were removed *in vacuo* and the residue extracted with pentane (5 mL). The resulting solution was concentrated to *ca.* 2 mL and colourless crystals of 10 were obtained which were suitable for X-ray crystallography. These were isolated by filtration and washed with cold pentane (2 × 1 mL). Yield: 16.6 mg (14.4%). ^1^H NMR (500 MHz, C_6_D_6_, 298 K): *δ*_H_ 1.27 (d, ^3^*J*_H,H_ = 6.9 Hz, 12H, CH_3_ of Dipp), 1.29 (d, ^3^*J*_H,H_ = 6.9 Hz, 12H, CH_3_ of Dipp), 3.43 (sept, ^3^*J*_H,H_ = 6.9 Hz, 4H,CH̲(CH_3_)_2_ of Dipp), 6.19 (s, 2H, CH of boryl), 6.82 (m, *m*-ArH of Ph), 6.89 (m, *p*-ArH of Ph), 7.17 (m, *m*-ArH of Dipp), 7.22 (m, *p*-ArH of Dipp), 7.46 (m, *o*-ArH of Ph), 8.65 (s, PhC(H)=N). ^11^B{^1^H} NMR (160 MHz, C_6_D_6_, 298 K): *δ*_B_ 24.6 (boryl). ^13^C{^1^H} NMR (126 MHz, C_6_D_6_, 298 K): *δ*_C_ 24.2, 24.4 (CH_3_ of Dipp), 28.8 (C̲H(CH_3_)_2_ of Dipp), 119.0 (CH of boryl), 123.7 (*m*-Ar of Dipp), 128.6 (*p*-Ar of Dipp),128.6 (*o*-Ar of Ph), 128.7 (*m*-Ar of Ph), 131.2 (*p*-Ar of Ph), 138.8 (CN of Dipp), 146.6 (*o*-Ar of Dipp), 167.8 (C=N), overlap by solvent peak (Ph(C̲)–C=N).

## Conclusions

In conclusion, we report on the reaction chemistry of an unprecedented ‘inorganic cumulene’ – featuring a five-atom BNBNB chain – in particular with respect to species containing C=O (and related) multiple bonds. The intrinsic polarity of the BNBNB chain (featuring electronic-rich nitrogen centres) is demonstrated by metathesis chemistry with electrophilic heteroallenes such as CO_2_, and with organic carbonyl compounds. Metathesis leads to {(HCDippN)_2_}BN transfer chemistry *via* consecutive [2+2] cyclo-addition/cyclo-reversion steps. Transfer of the imide unit to [CO], [CS], [PP{(NDippCH_2_)_2_}] and [C(H)Ph] moieties generates the (boryl)N=C=X systems (X = O, S, PP{(NDippCH_2_)_2_}, C(H)Ph), *via* processes driven thermodynamically by B–O bond formation. An isolable complex of stoichiometry K[(boryl)NB(O)OC(H)Ph] (9), formed *via* [2+2] cycloaddition of PhCHO to [(boryl)N=B=O]^−^ can be shown to be an intermediate in the formation of (boryl)N=C(H)Ph, and provides corroborating evidence for a DFT-calculated mechanism proceeding *via* consecutive bora-Wittig steps.

## Data availability

The data on which this study is based are included in the ESI.†

## Author contributions

JT and CH carried out the syntheses and measured the characterizing data. AEC and LPG measured the single crystal X-ray diffraction data and solved the structures. AEC and JT carried out the quantum chemical calculations. SA and JT wrote the draft manuscript. SA and JMG managed the project.

## Conflicts of interest

There are no conflicts to declare.

## Supplementary Material

SC-OLF-D4SC07487D-s001

SC-OLF-D4SC07487D-s002
