# Supplementary material for: Metathesis chemistry of inorganic cumulenes driven by B–O bond formation
Source: Chem Sci. 2024 Dec 18;16(5):2231–7. doi: 10.1039/d4sc07487d (PMC11706233; doi:10.1039/d4sc07487d)
Supplement: SC-016-D4SC07487D-s001 [file SC-016-D4SC07487D-s001.pdf]

Supplementary Information (SI) for Chemical Science.
This journal is © The Royal Society of Chemistry 2025

# Metathesis chemistry of inorganic cumulenes driven by B–O bond formation

Jianqin Tang,[a] Chenyang Hu,[a,b] Agamemnon E. Crumpton,[a] Liam P. Griffin,[a] Jose M. Goicoechea,*[,b] Simon Aldridge*[,a]

Inorganic Chemistry Laboratory, Department of Chemistry, University of Oxford, South Parks Road, Oxford, OX1 3QR (UK)

Department of Chemistry, Indiana University, 800 E. Kirkwood Ave, Bloomington, IN 47405 (USA)

simon.aldridge@chem.ox.ac.uk; jgoicoec@iu.edu

**Supporting information** (58 pages)

1. General considerations ........ S2

2. Preparation of starting materials ........ S2

3. Synthetic procedures, characterising data and representative spectra of new compounds ........ S3

4. Details of X-ray crystallography ........ S19

5. Details of quantum chemical calculations ........ S24

6. References for supporting information ........ S58

## 1. General considerations

All experiments were carried out under an atmosphere of dry argon or dinitrogen using standard Schlenk line or dry-box techniques. Solvents were degassed by sparging with argon and dried by passing through a column of appropriate drying agent using a commercially available Braun SPS and stored over potassium mirror under argon in a Teflon valve ampoule. NMR spectra were measured in $d_6$-benzene, with the solvent being dried over calcium hydride or molten potassium respectively, distilled, degassed by three freeze-pump-thaw-cycles and stored over 3 Å sieves. NMR samples were prepared under argon in 5 mm Wilmad 507-PP tubes fitted with J. Young Teflon valves. $^{1}$H and $^{13}$C{$^{1}$H} NMR spectra were measured on a Bruker Avance III HD Nanobay 400 MHz or Bruker Avance III 500 MHz spectrometer, and referenced internally to residual protio-solvent ($^{1}$H) or solvent ($^{13}$C) resonances; resonances are reported relative to tetramethylsilane (δ = 0 ppm). Assignments were confirmed using two-dimensional $^{1}$H-$^{1}$H, $^{13}$C-$^{1}$H, NMR correlation experiments. Chemical shifts are quoted in δ (ppm) and coupling constants in Hz. The reported yields are the yields obtained after crystallisation and subsequent measurement of the material by single crystal-ray diffraction if not stated otherwise. Elemental analyses were carried out by London Metropolitan University or Elemental Microanalysis Ltd, Okehampton, Devon, UK.

## 2. Preparation of starting materials

K[(HCDippN)$_2$BNBNB(NDippCH)$_2$], **1**, [K$_2${(HCDippN)$_2$BN}$_2$B][{(C$_6$F$_5$)$_3$BCCH$_2$(DippN)$_2$BN}$_2$B], **3**, and (H$_2$CDippN)$_2$PPCO were prepared by literature procedures.[S1,S2]

## 3. Synthetic procedures, characterising data and representative spectra of new compounds

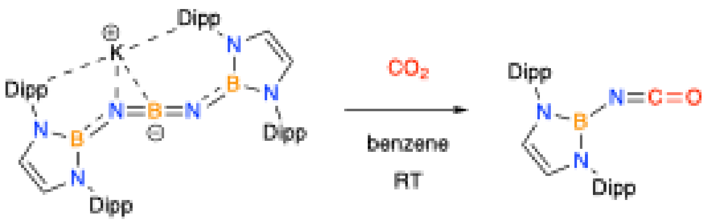


**Scheme S1.** Synthesis of $(HCDippN)_2BNCO$, **5**.

**$(HCDippN)_2BNCO$, 5.** A solution of **1** (100 mg 0.117 mmol) in benzene (5 mL) was degassed by three freeze-pump-thaw cycles, before $CO_2$ was admitted (at ca. 1 atm pressure), leading to a near colourless solution. After stirring for 12 h, volatiles were removed in vacuo and the resulting pale solid dissolved in 3 mL pentane. The solution was concentrated to ca. 1 mL and crystals of **5** were obtained upon standing at room temperature that were suitable for X-ray crystallography. These were isolated by filtration and washed with cold pentane (2 x 1 mL).

Yield: 89.6 mg (90.0 %)

$^1$H NMR (500 MHz, $C_6D_6$, 298 K): $\delta_H$ 1.18 (d, $^3J_{H,H}$ = 6.8 Hz, 12H, $CH_3$ of Dipp), 1.25 (d, $^3J_{H,H}$ = 6.8 Hz, 12H, $CH_3$ of Dipp), 3.14 (sept, $^3J_{H,H}$ = 6.8 Hz, 4H,C<u>H</u>$(CH_3)_2$ of Dipp), 5.95 (s, 2H, CH of boryl), 7.12 (m, 4H, m-ArH of Dipp), 7.21, (m, 2H, p-ArH of Dipp).

$^{11}$B{$^1$H} NMR (128 MHz, $C_6D_6$, 298 K): $\delta_B$ 17.9 (boryl).

$^{13}$C{$^1$H} NMR (126 MHz, $C_6D_6$, 298 K): $\delta_C$ 24.1, 24.3 ($CH_3$ of Dipp), 28.8 (<u>C</u>H$(CH_3)_2$ of Dipp), 118.2 (CH of boryl), 123.9 (m-Ar of Dipp), 128.4 (p-Ar of Dipp), 136.8 (CN of Dipp), 146.5 (o-Ar of Dipp), not observed (N=C=O).

Elemental microanalysis: calc. for $C_{27}H_{36}BN_3O$ (%): C 75.52 H 8.45 N 9.79; meas. C 75.82 H 8.51 N 9.54.

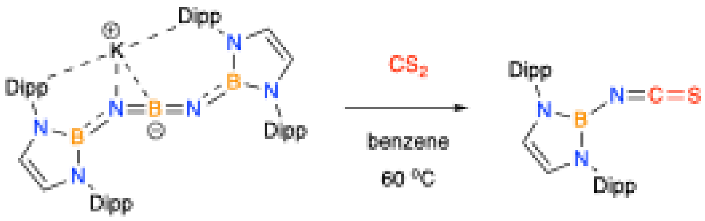

1 6

**Scheme S2.** Synthesis of $(HCDippN)_2BNCS$, **6**.

**$(HCDippN)_2BNCS$, 6. 1** (100 mg 0.117 mmol) was dissolved in benzene (10 mL) and a drop of $CS_2$ (ca. 0.02 mL, excess) added. The solution was heated at 60 °C overnight with a colour change from orange to red. Volatiles were removed in vacuo and the residue extracted with pentane (10 mL). The resulting solution was concentrated to ca. 2 mL and colourless crystals of **6** were obtained upon standing at room temperature which were suitable for X-ray crystallography. These were isolated by filtration and washed with cold pentane (2 x 1 mL).

Yield: 51.3 mg (52.0 %)

$^1$H NMR (500 MHz, $C_6D_6$, 298 K): $\delta_H$ 1.17 (d, $^3J_{H,H}$ = 6.8 Hz, 12H, $CH_3$ of Dipp), 1.29 (d, $^3J_{H,H}$ = 6.8 Hz, 12H, $CH_3$ of Dipp), 3.12 (sept, $^3J_{H,H}$ = 6.8 Hz, 4H,C<u>H</u>$(CH_3)_2$ of Dipp), 5.91 (s, 2H, CH of boryl), 7.13(m, 4H, m-ArH of Dipp), 7.20 (m, 2H, p-ArH of Dipp).

$^{11}$B{$^1$H} NMR (160 MHz, $C_6D_6$, 298 K): $\delta_B$ 16.1 (boryl).

$^{13}$C{$^1$H} NMR (126 MHz, $C_6D_6$, 298 K): $\delta_C$ 24.0, 24.5 ($CH_3$ of Dipp), 28.9 (<u>C</u>H$(CH_3)_2$ of Dipp), 118.6 (CH of boryl), 123.9 (m-Ar of Dipp), 128.6 (p-Ar of Dipp), 136.4 (CN of Dipp), 146.3 (o-Ar of Dipp), not observed (N=C=S).

Elemental microanalysis: calc. for $C_{27}H_{36}BN_3S$ (%): C 72.80 H 8.15 N 9.43; meas. C 72.76 H 8.26 N 9.17.

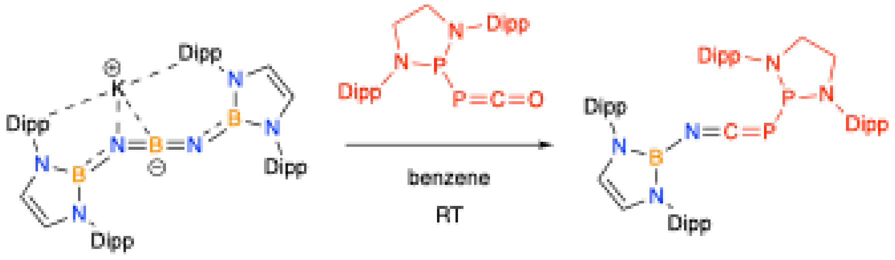


**Scheme S3.** Synthesis of $(HCDippN)_2BNCPP(NDippCH_2)_2$, **7**.

**$(HCDippN)_2BNCPP(NDippCH_2)_2$, 7.** To a mixture of **1** (80 mg, 0.094 mmol) and $(CH_2DippN)_2PPCO$ (69.3 mg, 0.148 mmol) in a 25 mL Schlenk flask was added 5 mL benzene. The resulting solution was stirred for 12 h with a colour change to dark green. Volatiles were removed in vacuo and the residue extracted with pentane (3 mL). The resulting solution was concentrated to ca. 1 mL and colourless crystals of **7** were obtained upon standing at room temperature which were suitable for X-ray crystallography. These were isolated by filtration and washed with cold pentane (2 x 1 mL).

Yield: 88.6 mg (55.2 %)

$^1H$ NMR (500 MHz, $C_6D_6$, 298 K): $\delta_H$ 1.05 (d, $^3J_{H,H}$ = 7.0 Hz, 12H, $CH_3$ of boryl Dipp),1.13 (d, $^3J_{H,H}$ = 7.0 Hz, 12H, $CH_3$ of boryl Dipp), 1.19, 1.25, (d, $^3J_{H,H}$ = 6.6 Hz, 12H, $CH_3$ of phosphanyl Dipp),1.31 (d, $^3J_{H,H}$ = 6.6 Hz, 12H, $CH_3$ of phosphanyl Dipp), 3.03 (sept, $^3J_{H,H}$ = 7.0 Hz, 4H,C<u>H</u>$(CH_3)_2$ of boryl Dipp), 3.11, 3.58 (m, 4H, $CH_2$ of phosphanyl), 3.48, 3.76 (m, 4H,C<u>H</u>$(CH_3)_2$ of phosphanyl Dipp), 5.86 (s, 2H, CH of boryl), 7.04 (m, 4H, m-ArH of boryl Dipp), 7.10 (m, 6H, ArH of phosphanyl Dipp), 7.18 (m, 2H, p-ArH of boryl Dipp).

$^{11}B\{^1H\}$ NMR (160 MHz, $C_6D_6$, 298 K): $\delta_B$ 16.0 (boryl).

$^{31}P$ NMR (162 MHz, $C_6D_6$, 298 K): $\delta_P$ -183.3 (d, P<u>P</u>=C, $^1J_{P,P}$ = 298.0 Hz),165.6 (d, <u>P</u>P=C, $^1J_{P,P}$ = 298.0 Hz).

$^{13}C\{^1H\}$ NMR (126 MHz, $C_6D_6$, 298 K): $\delta_C$ 23.9, 24.4 ($CH_3$ of Dipp), 24.7, 25.2 (d, $^5J_{C,p}$ = 3.6 Hz, $CH_3$ of phosphanyl Dipp), 25.7,25.9 ($CH_3$ of phosphanyl Dipp), 28.8 (<u>C</u>H$(CH_3)_2$ of boryl Dipp), 28.9, 29.5 (d, $^4J_{C,P}$ = 7.3 Hz, <u>C</u>H$(CH_3)_2$ of phosphanyl Dipp), 54.9 (d, $^2J_{C,p}$ = 8.7 Hz, $CH_2$ of phosphanyl), 118.5 (CH of boryl), 123.6 (m-Ar of boryl Dipp), 123.9 (m-Ar of phosphanyl Dipp), 124.9 (p-Ar of phosphanyl Dipp), 127.5 (o-Ar of boryl Dipp), 136.8 (CN of boryl Dipp), 137.7, 137.8 (CN of phosphanyl Dipp), 146.2 (o-Ar of boryl Dipp), 148.7, 150.4 (o-Ar of phosphanyl Dipp), not observed (N=C=P)

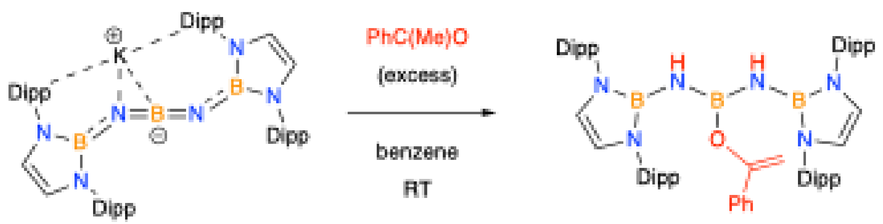


1 8

**Scheme S4.** Synthesis of [{(HCDippN)$_2$BN}$_2$B]OC(CH$_2$)Ph, **8**.

**[{(HCDippN)$_2$BN}$_2$B]OC(CH$_2$)Ph, 8. 1** (15 mg 0.018 mmol) was dissolved in benzene (4 mL) and a drop of acetophenone (0.01 mL, excess) added. The reaction mixture was stirred for 1 h with an accompanying colour change to light yellow. Volatiles were removed in vacuo and the residue extracted with pentane (3 mL). The resulting solution was concentrated to ca. 0.5 mL and yellow crystals of **8** were obtained upon standing at room temperature which were suitable for X-ray crystallography. These were isolated by filtration and washed with cold pentane (2 x 1 mL).

Yield: 7.8 mg (47.0 %)

$^1$H NMR (500 MHz, $C_6D_6$, 298 K): $\delta_H$ 1.16 (d, $^3J_{H,H}$ = 6.6 Hz, 24H, CH$_3$ of Dipp), 1.18 (d, $^3J_{H,H}$ = 6.6 Hz, 24H, CH$_3$ of Dipp), 2.56 (br, 2H, NH), 3.12, 4.43 (s, each 1H, C=CH$_2$), 3.28 (sept, $^3J_{H,H}$ = 6.8 Hz, 8H,C<u>H</u>(CH$_3$)$_2$ of Dipp), 5.97 (s, 2H, CH of boryl), 7.07 (m, 17H, ArH of Dipp).

$^{11}$B{$^1$H} NMR (160 MHz, $C_6D_6$, 298 K): $\delta_B$ 23.9 (boryl).

$^{13}$C{$^1$H} NMR (126 MHz, $C_6D_6$, 298 K): $\delta_C$ 23.8, 25.5 (CH$_3$ of Dipp), 28.8 (<u>C</u>H(CH$_3$)$_2$ of Dipp), 97.7 (C=<u>C</u>H$_2$), 118.6 (CH of boryl), 124.2 (m-Ar of Dipp), 125.3, 128.8, 136.7 (Ph), 127.4 (p-Ar of Dipp), 139.6 (CN of Dipp), 146.3 (o-Ar of Dipp), 154.4 (<u>C</u>=CH$_2$).

Elemental microanalysis: calc. for $C_{60}H_{81}B_3N_6O$ (%): C 77.09 H 8.73 N 8.99; meas. C 76.89 H 8.51 N 9.12.

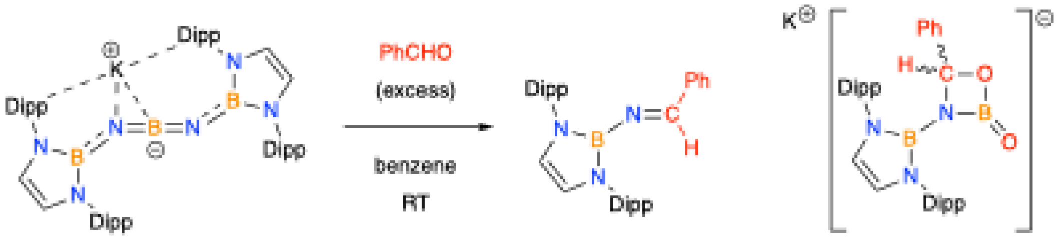


1 10 9

**Scheme S5.** Synthesis of $(HCDippN)_2BN[\kappa^2\text{-}(C,B)\text{-}C(H)PhBO_2]$, **9** and $(HCDippN)_2BNCHPh$, **10**.

**$(HCDippN)_2BN[\kappa^2\text{-}(C,B)\text{-}C(H)PhBO_2]$, 9**. **1**(100 mg 0.117 mmol) was dissolved in benzene (10 mL) and a drop of benzaldehyde (0.02 mL, excess) added. The resulting solution was stirred for 30 min, with an accompanying colour change to yellow. Volatiles were removed in vacuo, and the residue extracted with pentane (5 mL). The resulting solution was concentrated to ca. 0.5 mL and a mix of colourless crystals of **9** (unstable in solvent and briefly visible in NMR) and **10** were obtained which were suitable for X-ray crystallography.

**$(HCDippN)_2BNCHPh$, 10**. **1** (100 mg 0.117 mmol) was dissolved in benzene (10 mL) and a drop of benzaldehyde (0.02 mL, excess) added. The resulting solution was stirred for 12 h with a colour change to light yellow. Volatiles were removed in vacuo and the residue extracted with pentane (5 mL). The resulting solution was concentrated to ca. 2 mL and colourless crystals of **10** were obtained which were suitable for X-ray crystallography. These were isolated by filtration and washed with cold pentane (2 x 1 mL).

Yield: 16.6 mg (14.4 %)

$^1$H NMR (500 MHz, $C_6D_6$, 298 K): $\delta_H$ 1.27 (d, $^3J_{H,H}$ = 6.9 Hz, 12H, $CH_3$ of Dipp), 1.29 (d, $^3J_{H,H}$ = 6.9 Hz, 12H, $CH_3$ of Dipp), 3.43 (sept, $^3J_{H,H}$ = 6.9 Hz, 4H,C<u>H</u>$(CH_3)_2$ of Dipp), 6.19 (s, 2H, CH of boryl), 6.82 (m, m-ArH of Ph), 6.89 (m, p-ArH of Ph), 7.17 (m, m-ArH of Dipp), 7.22 (m, p-ArH of Dipp), 7.46 (m, o-ArH of Ph), 8.65 (s, PhC(H)=N).

$^{11}$B{$^1$H} NMR (160 MHz, $C_6D_6$, 298 K): $\delta_B$ 24.6 (boryl).

$^{13}$C{$^1$H} NMR (126 MHz, $C_6D_6$, 298 K): $\delta_C$ 24.2, 24.4 ($CH_3$ of Dipp), 28.8 (<u>C</u>H$(CH_3)_2$ of Dipp), 119.0 (CH of boryl), 123.7 (m-Ar of Dipp), 128.6 (p-Ar of Dipp),128.6 (o-Ar of Ph), 128.7 (m-Ar of Ph), 131.2 (p-Ar of Ph), 138.8 (CN of Dipp), 146.6 (o-Ar of Dipp), 167.8 (C=N), overlap by solvent peak (Ph(<u>C</u>)-C=N).

**Reactions of 3 with electrophiles**

With MeI. **3** (15 mg, 0.011 mmol) was dissolved in $C_6D_6$ (0.4 mL) and a drop of MeI (0.01 mL, excess) added. The reaction mixture was heated at 60 °C overnight in a J-Young NMR tube with the formation of a white precipitate; monitoring by multinuclear NMR revealed essentially quantitative formation of **4** and $B(C_6F_5)_3$ (Figures S18 and S19).

With $CO_2$. A solution of **3** (15 mg, 0.011 mmol) in $C_6D_6$ (0.4 mL) was degassed by three freeze-pump-thaw cycles, before $CO_2$ was admitted (at ca. 1 atm pressure), leading to a near colourless solution; monitoring by multinuclear NMR revealed essentially quantitative formation of **5** (Figure S20).

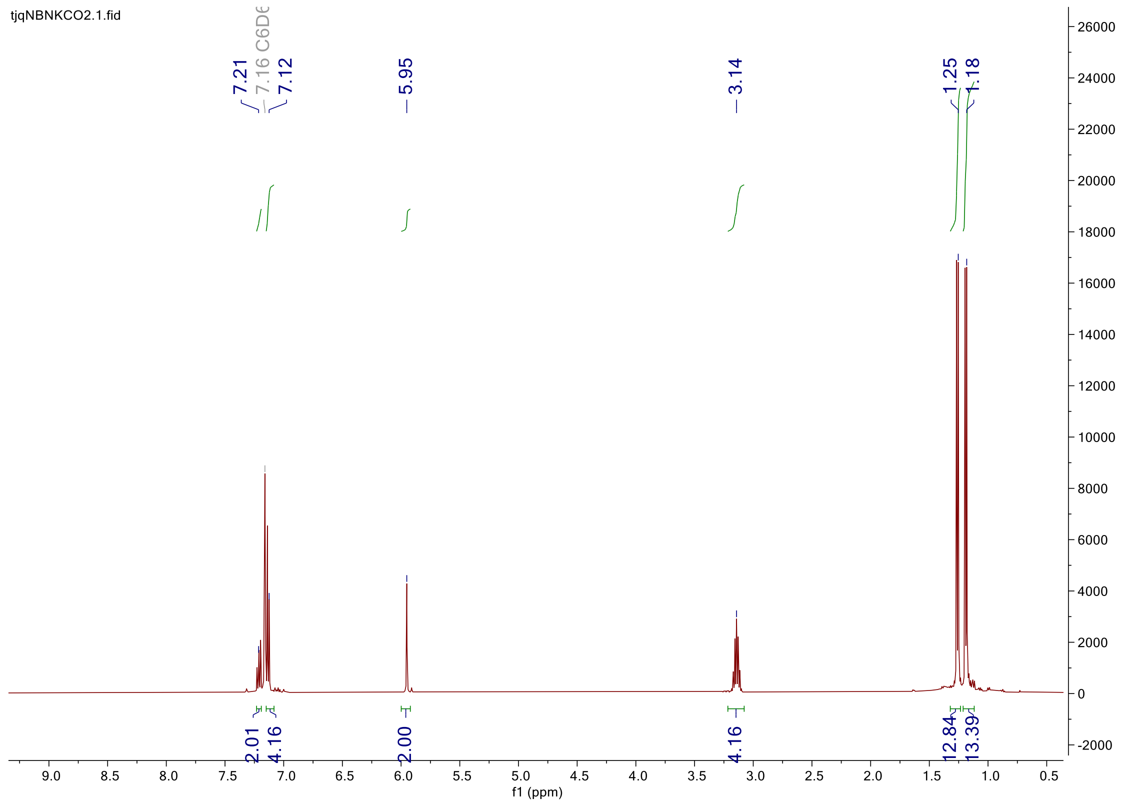


**Figure S1**: $^{1}H$ NMR spectrum of **5** (500 MHz, $C_6D_6$, 298 K).

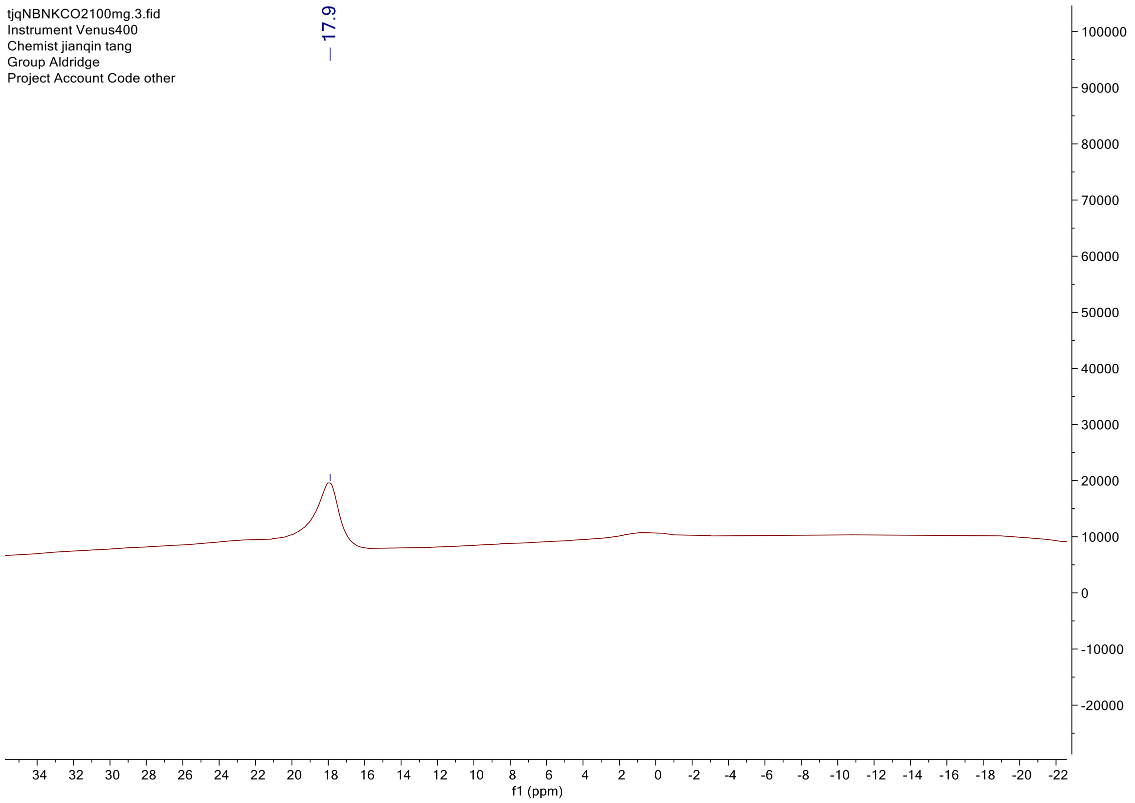


**Figure S2**: $^{11}B\{^{1}H\}$ NMR spectrum of **5** (128 MHz, $C_6D_6$, 298 K).

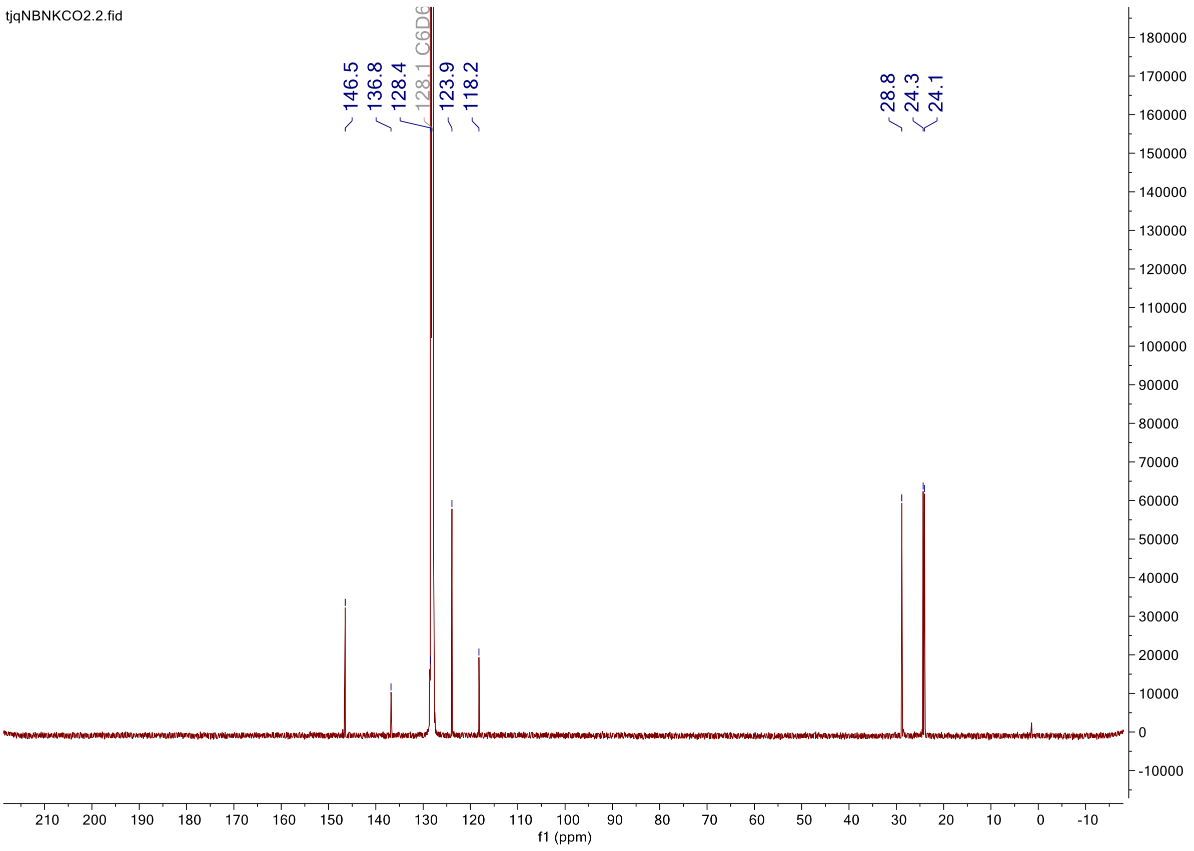


**Figure S3**: $^{13}C\{^1H\}$ NMR spectrum of **5** (126 MHz, $C_6D_6$, 298 K).

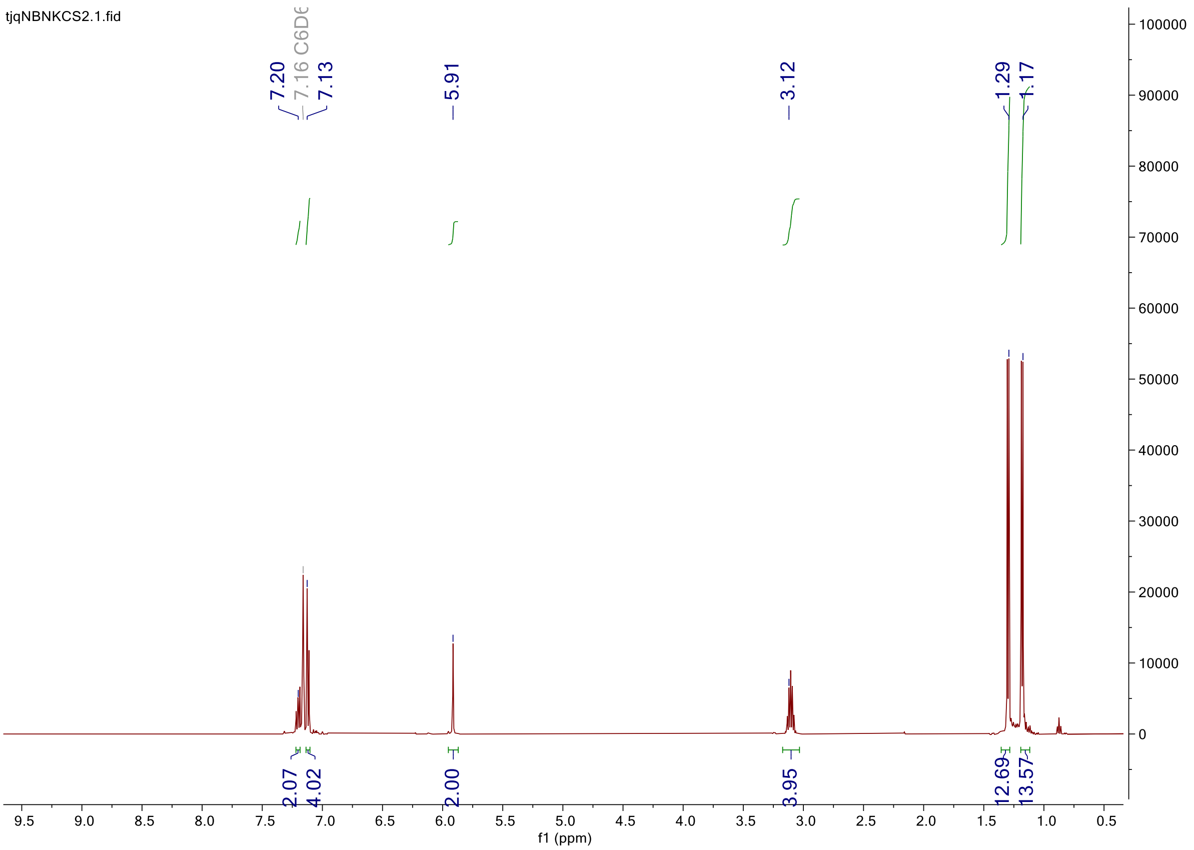


**Figure S4**: $^1H$ NMR spectrum of **6** (500 MHz, $C_6D_6$, 298 K).

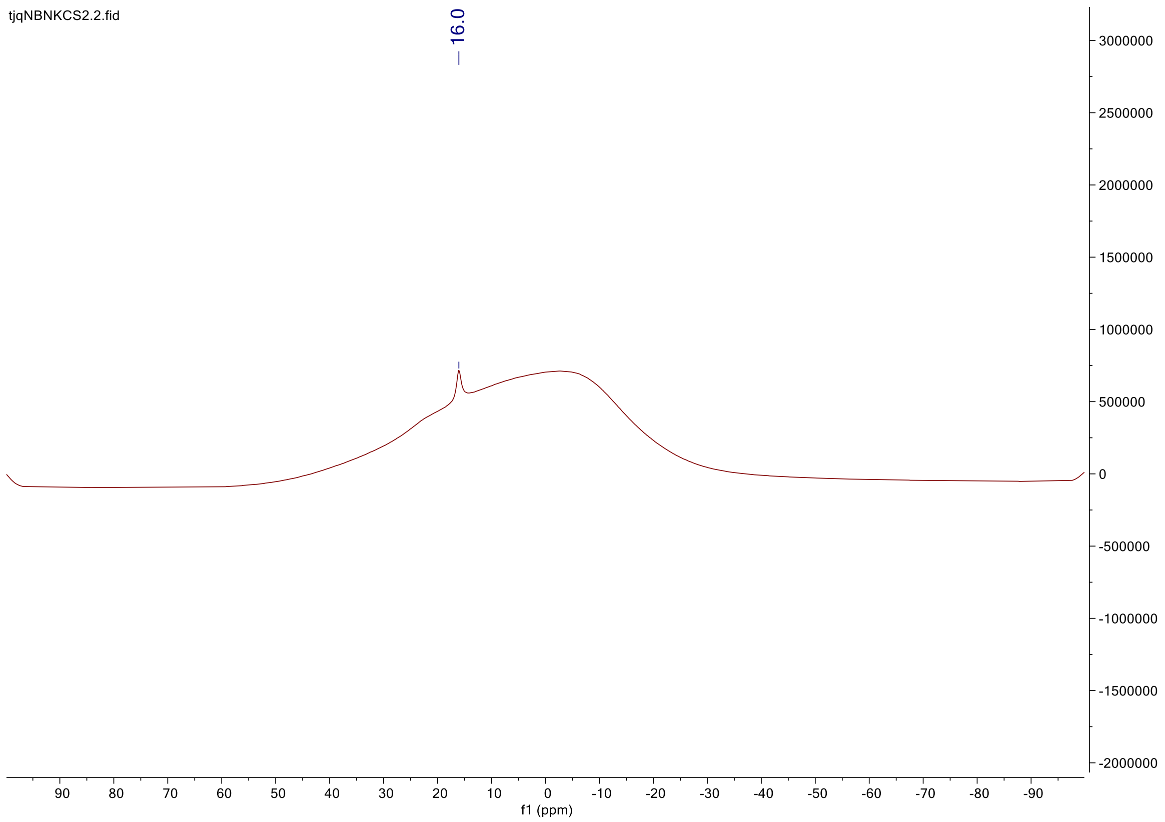


**Figure S5**: $^{11}B\{^{1}H\}$ NMR spectrum of **6** (160 MHz, $C_6D_6$, 298 K).

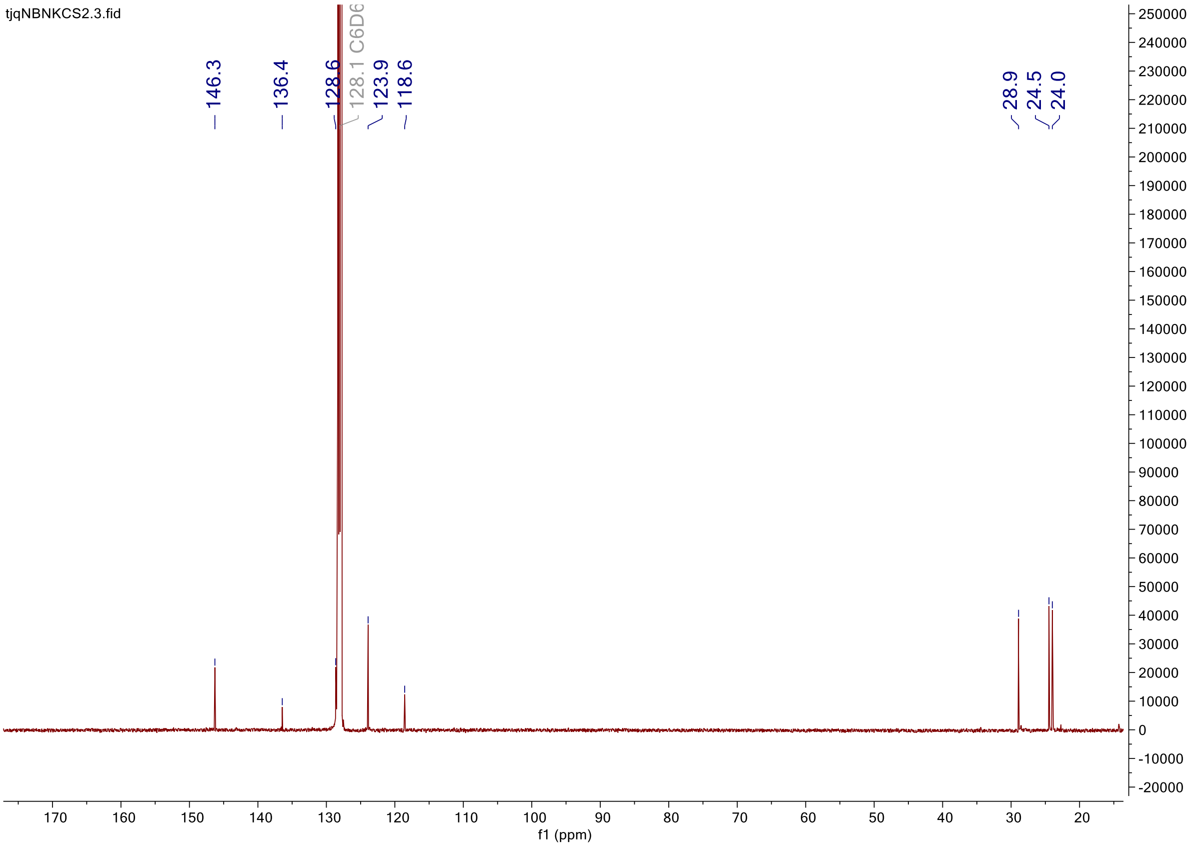


**Figure S6**: $^{13}C\{^{1}H\}$ NMR spectrum of **6** (126 MHz, $C_6D_6$, 298 K).

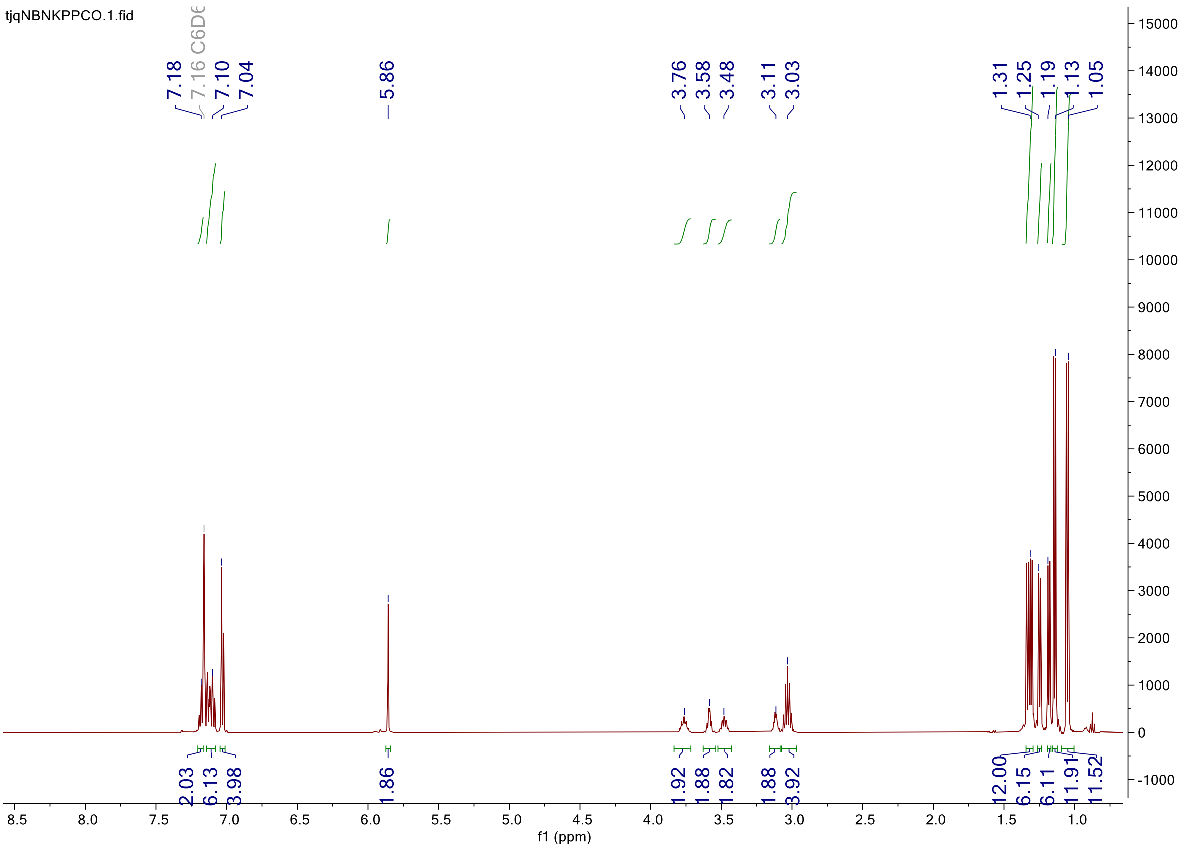


**Figure S7**: $^{1}H$ NMR spectrum of **7** (500 MHz, $C_6D_6$, 298 K).

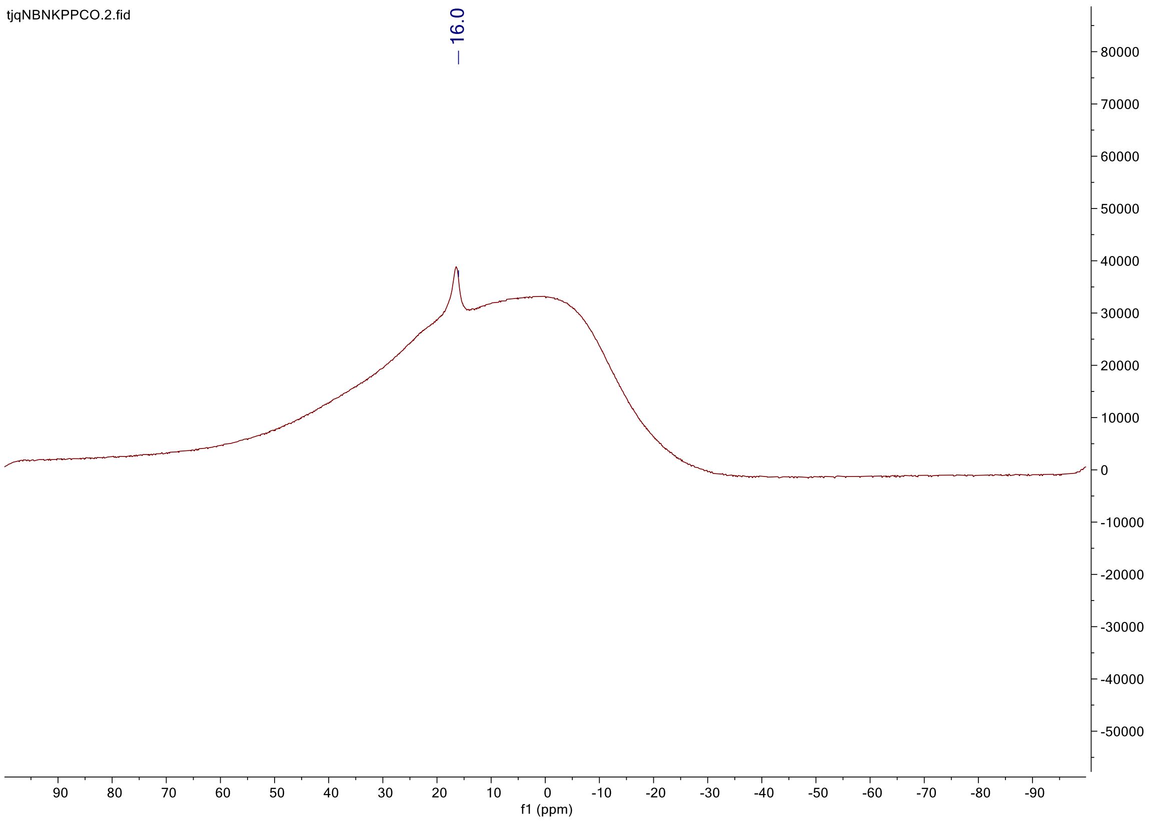


**Figure S8**: $^{11}B\{^{1}H\}$ NMR spectrum of **7** (160 MHz, $C_6D_6$, 298 K).

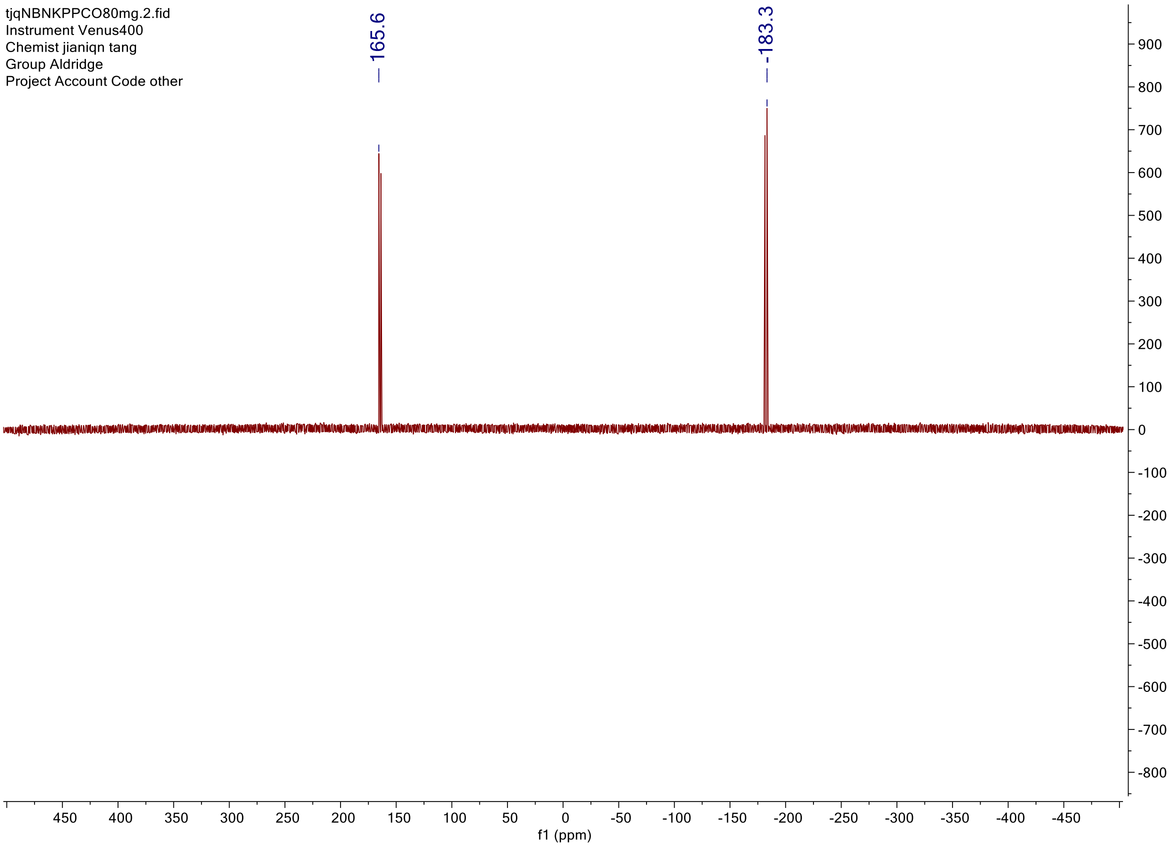


**Figure S9**: $^{31}$P NMR spectrum of **7** (162 MHz, $C_6D_6$, 298 K).

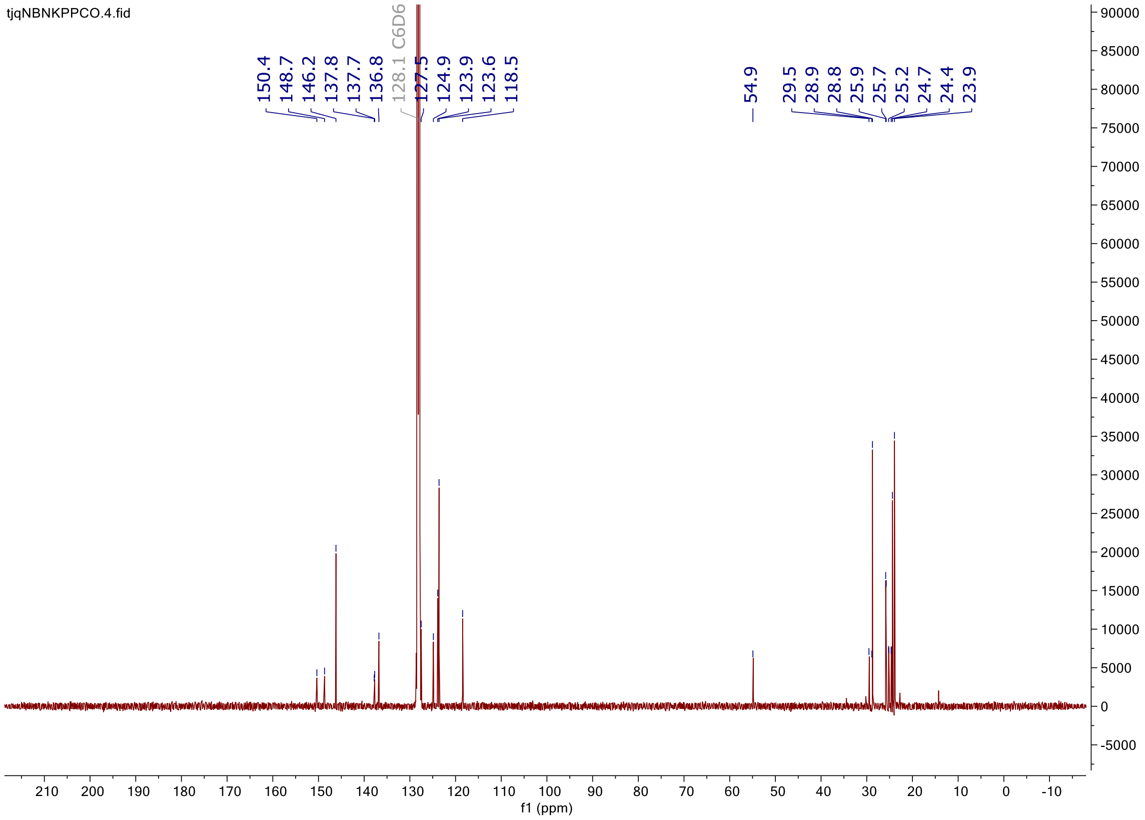


**Figure S10**: $^{13}$C{$^{1}$H} NMR spectrum of **7** (126 MHz, $C_6D_6$, 298 K).

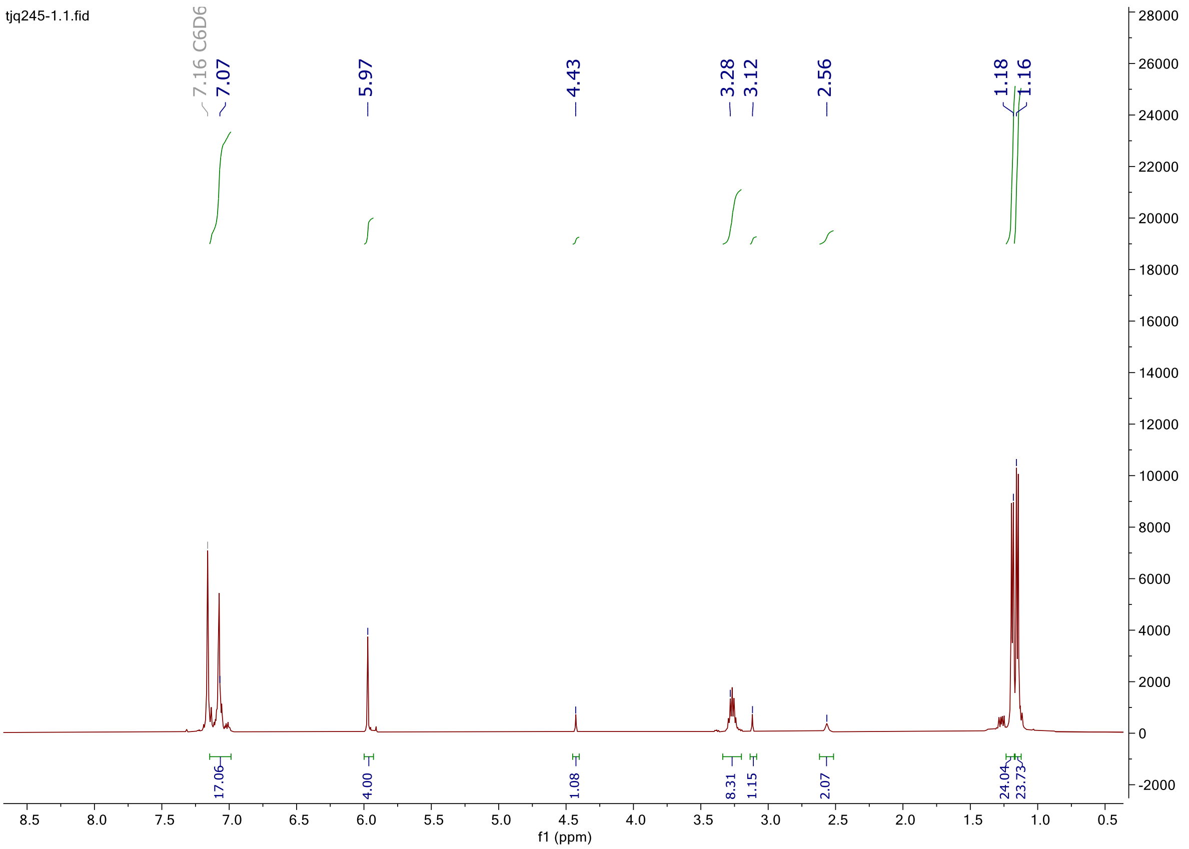


**Figure S11**: $^{1}H$ NMR spectrum of **8** (500 MHz, $C_6D_6$, 298 K).

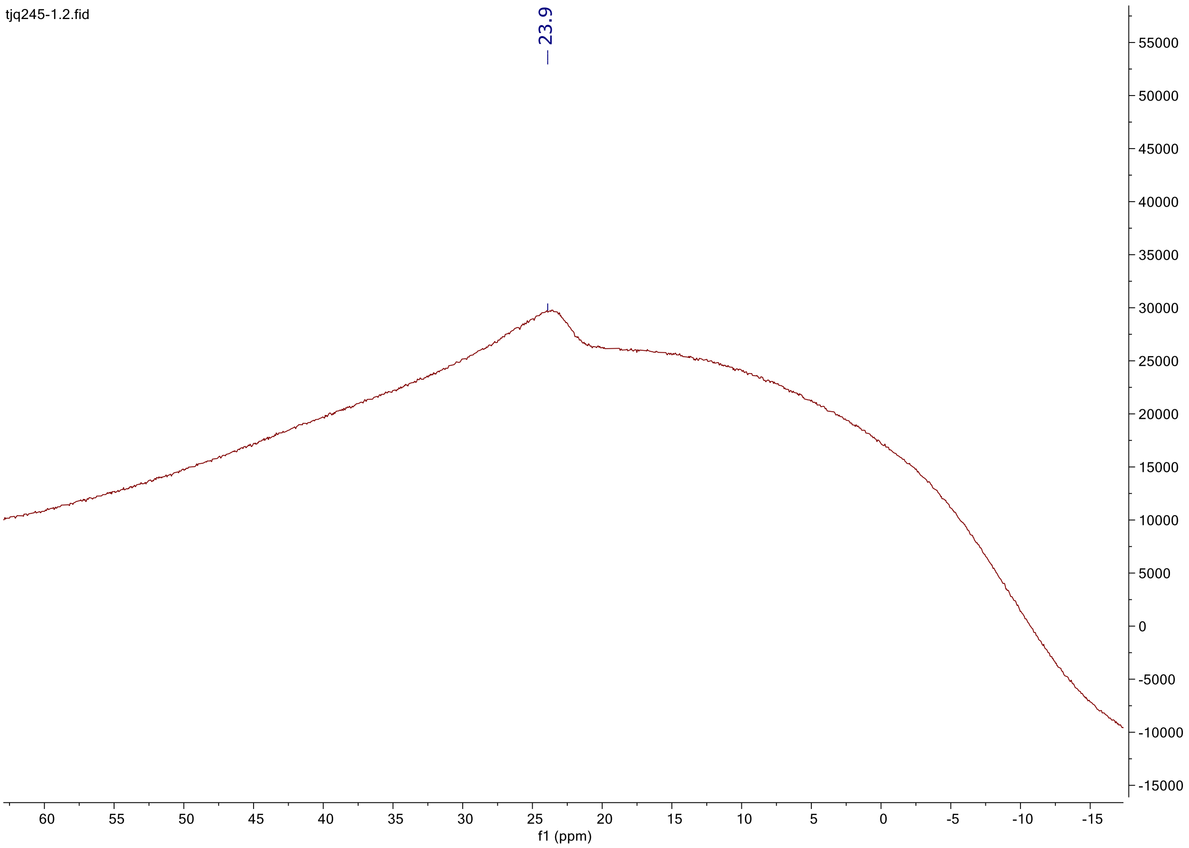


**Figure S12**: $^{11}B\{^{1}H\}$ NMR spectrum of **8** (160 MHz, $C_6D_6$, 298 K).

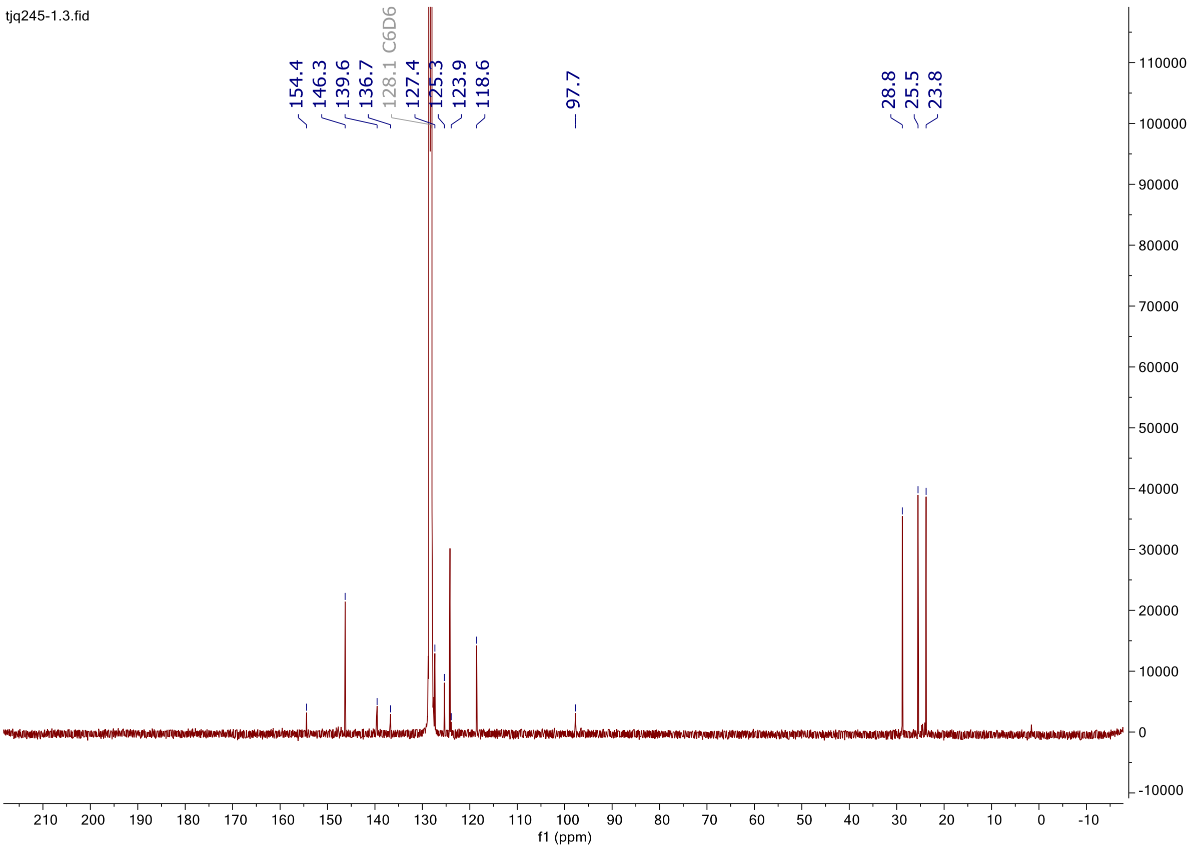


**Figure S13.** $^{13}C\{^1H\}$ NMR spectrum of **8** (126 MHz, $C_6D_6$, 298 K).

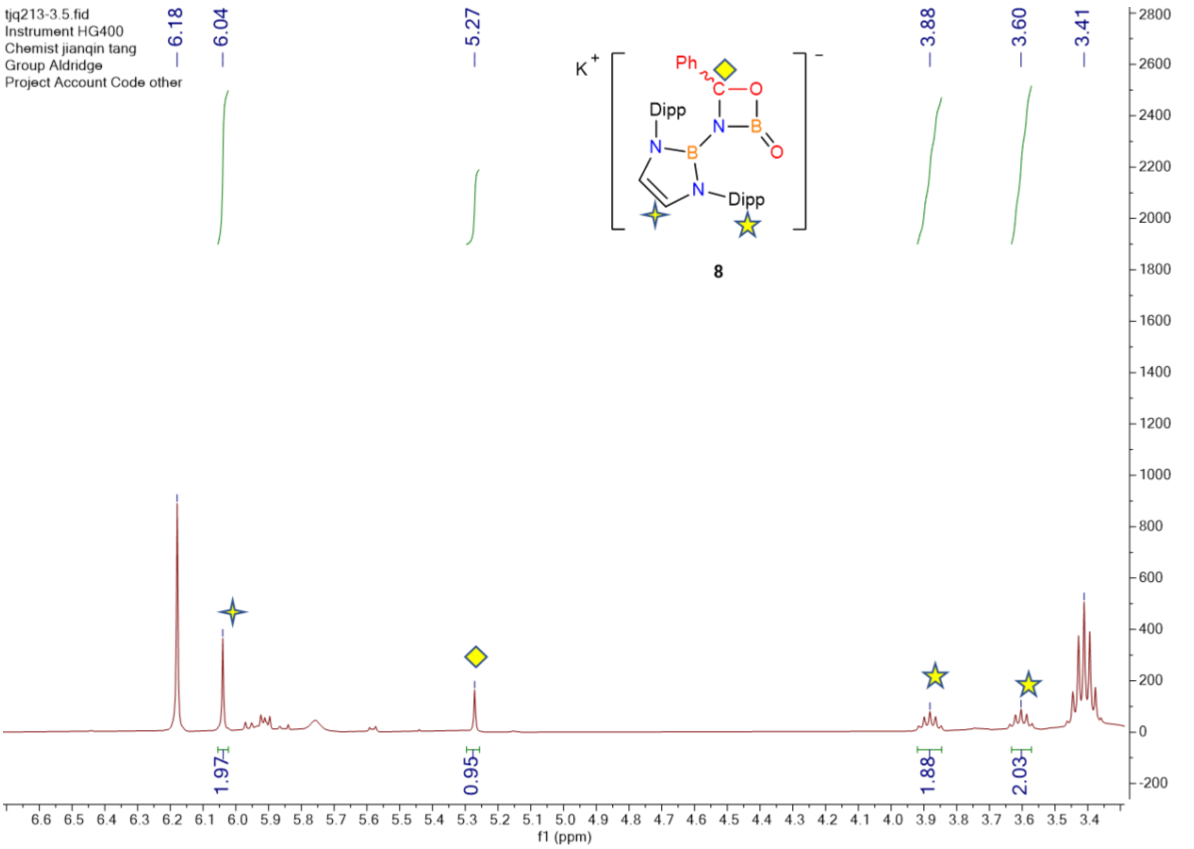


**Figure S14**: $^1H$ NMR spectrum(part) of **9** (400 MHz, $C_6D_6$, 298 K).

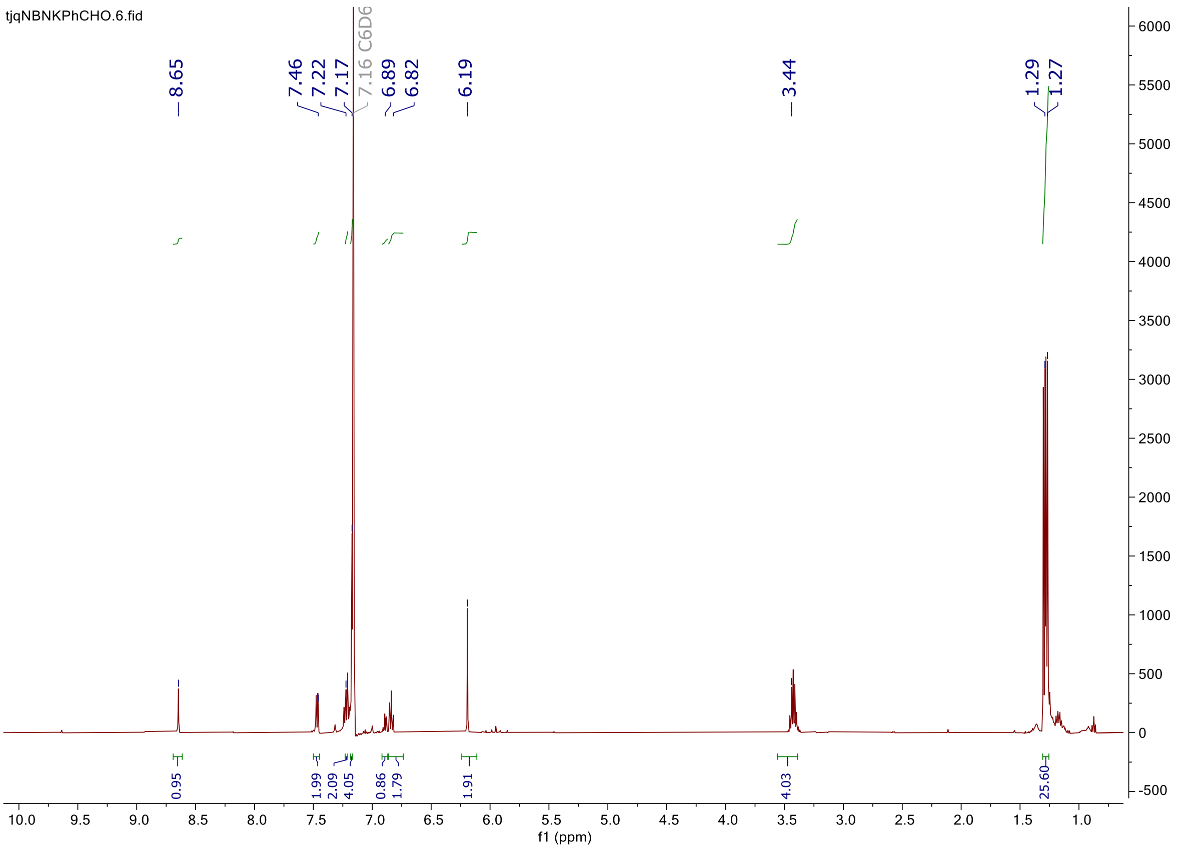


**Figure S15**: $^{1}$H NMR spectrum of **10** (500 MHz, $C_6D_6$, 298 K).

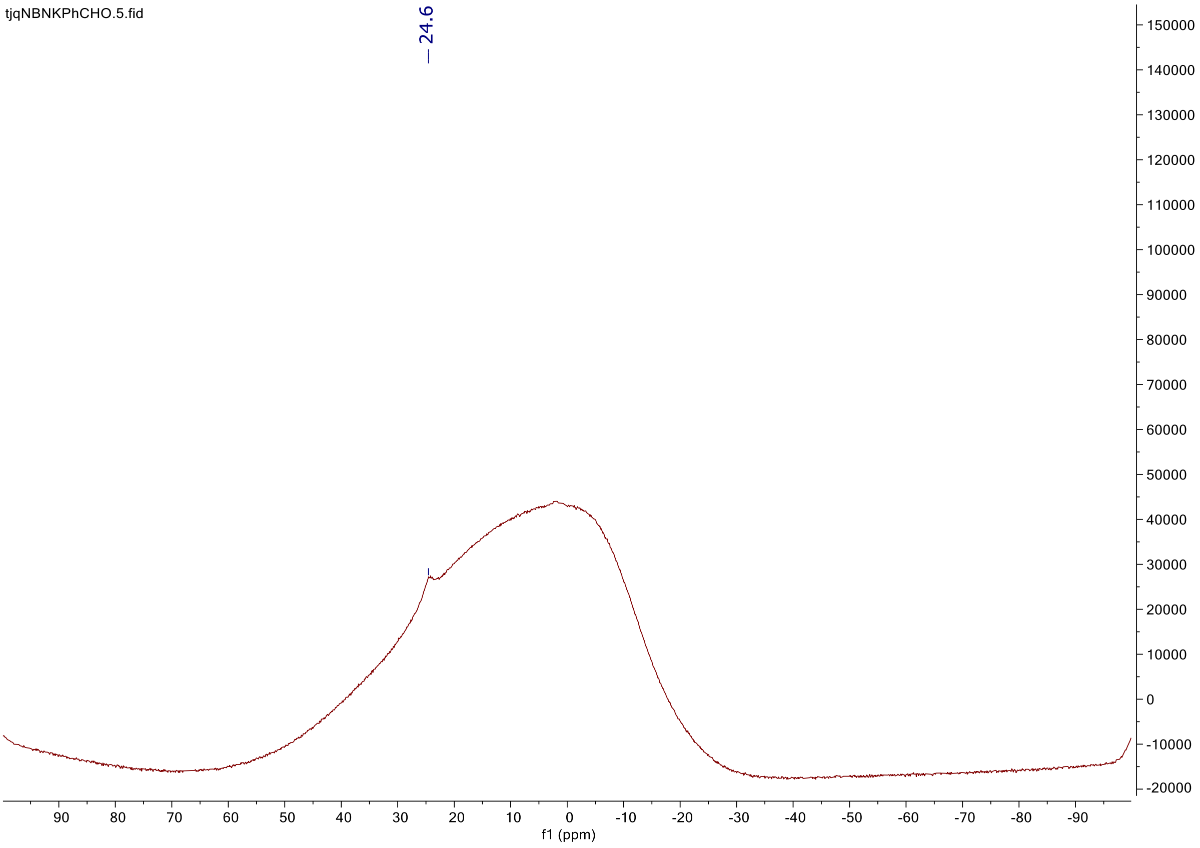


**Figure S16**: $^{11}$B{$^{1}$H} NMR spectrum of **10** (160 MHz, $C_6D_6$, 298 K).

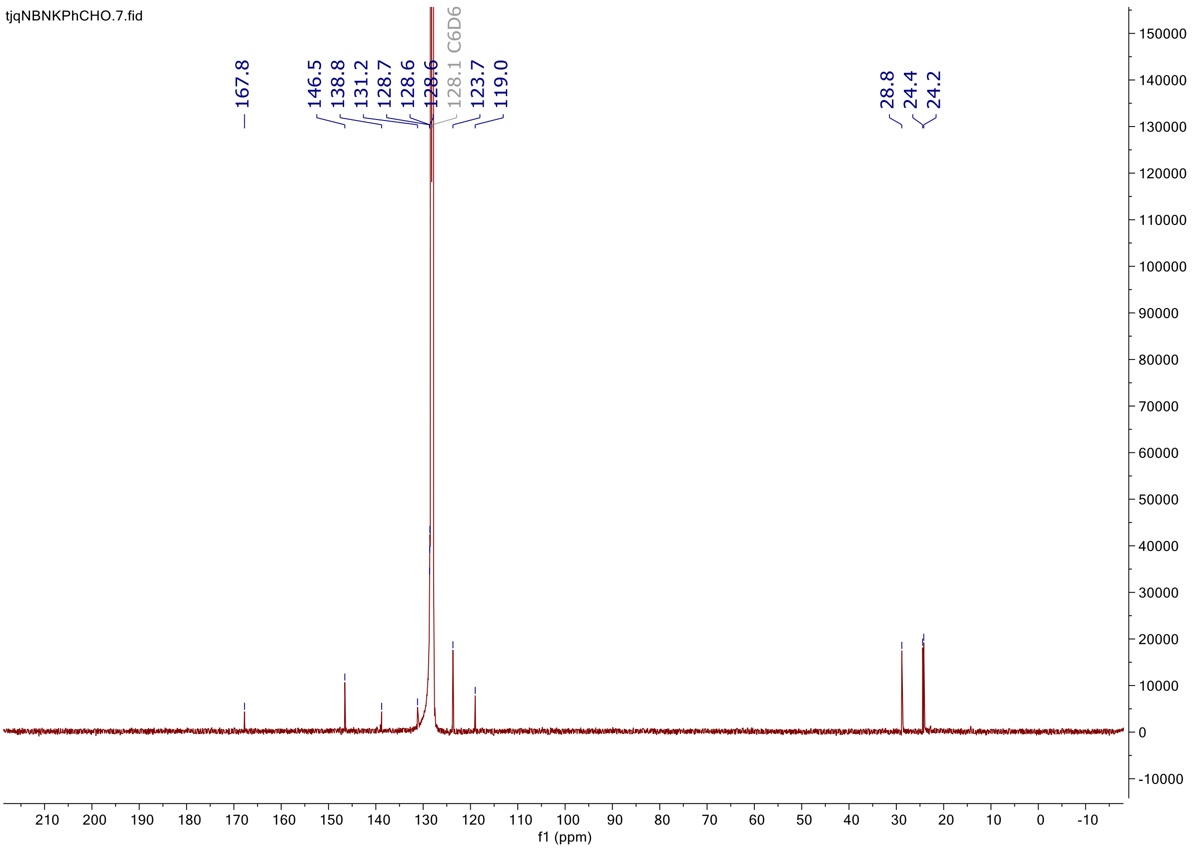


**Figure S17**: $^{13}C\{^{1}H\}$ NMR spectrum of **10** (126 MHz, $C_6D_6$, 298 K).

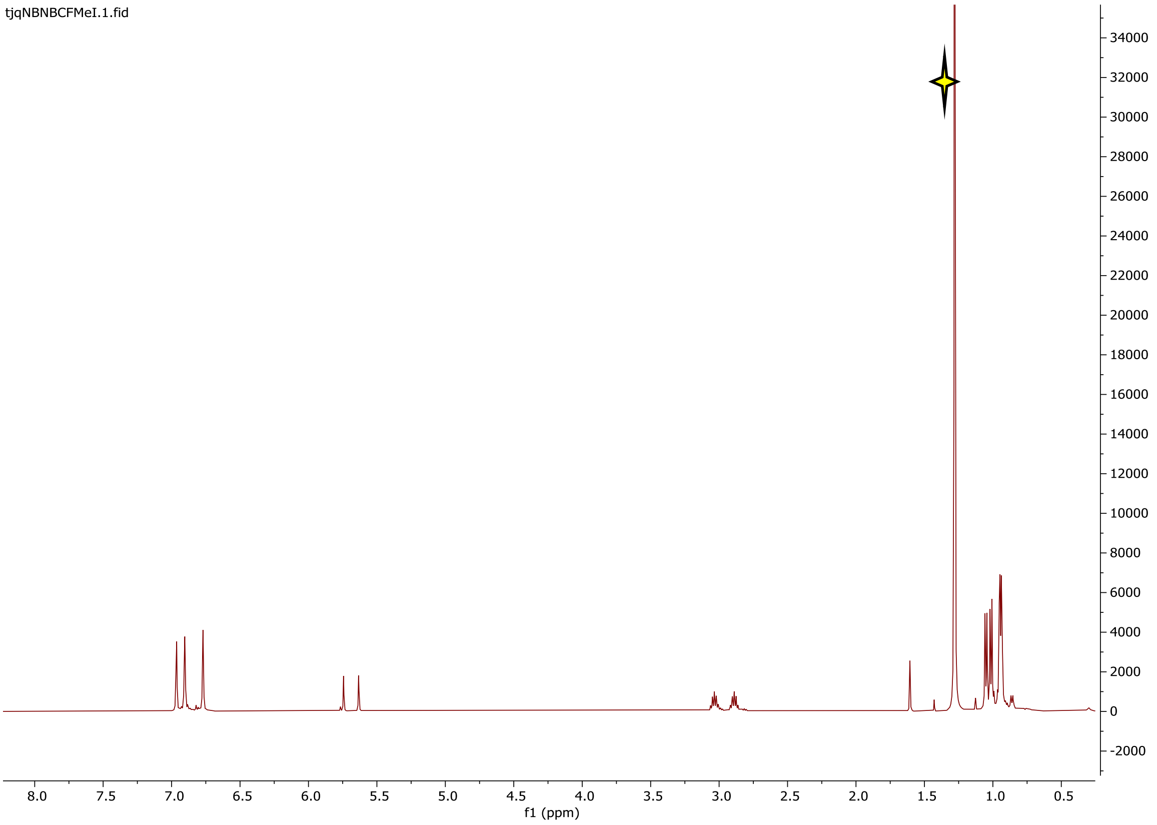


**Figure S18.** $^{1}H$ NMR monitoring of the reaction of **3** with MeI, showing the formation of **4** (500 MHz, $C_6D_6$, 298 K) (yellow star: excess MeI).

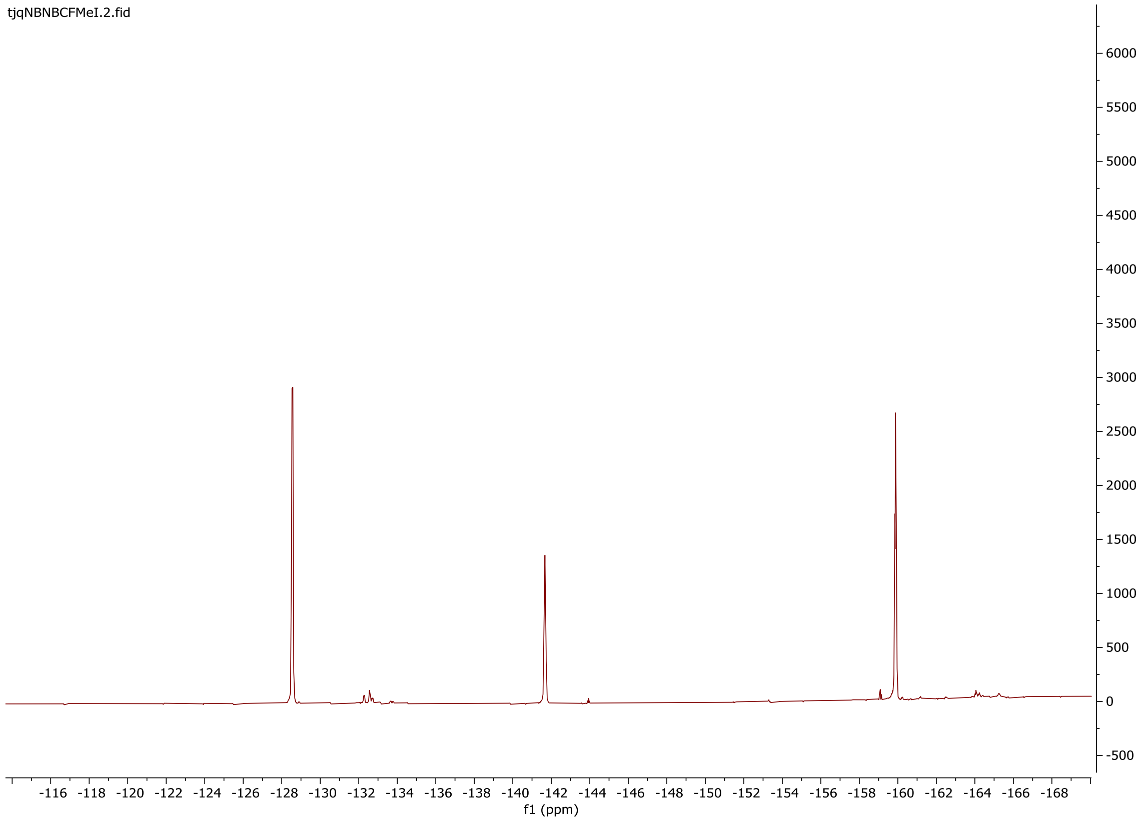


**Figure S19.** $^{19}F$ NMR monitoring of the reaction of **3** with MeI, showing the formation of $B(C_6F_5)_3$ (470 MHz, $C_6D_6$, 298 K).

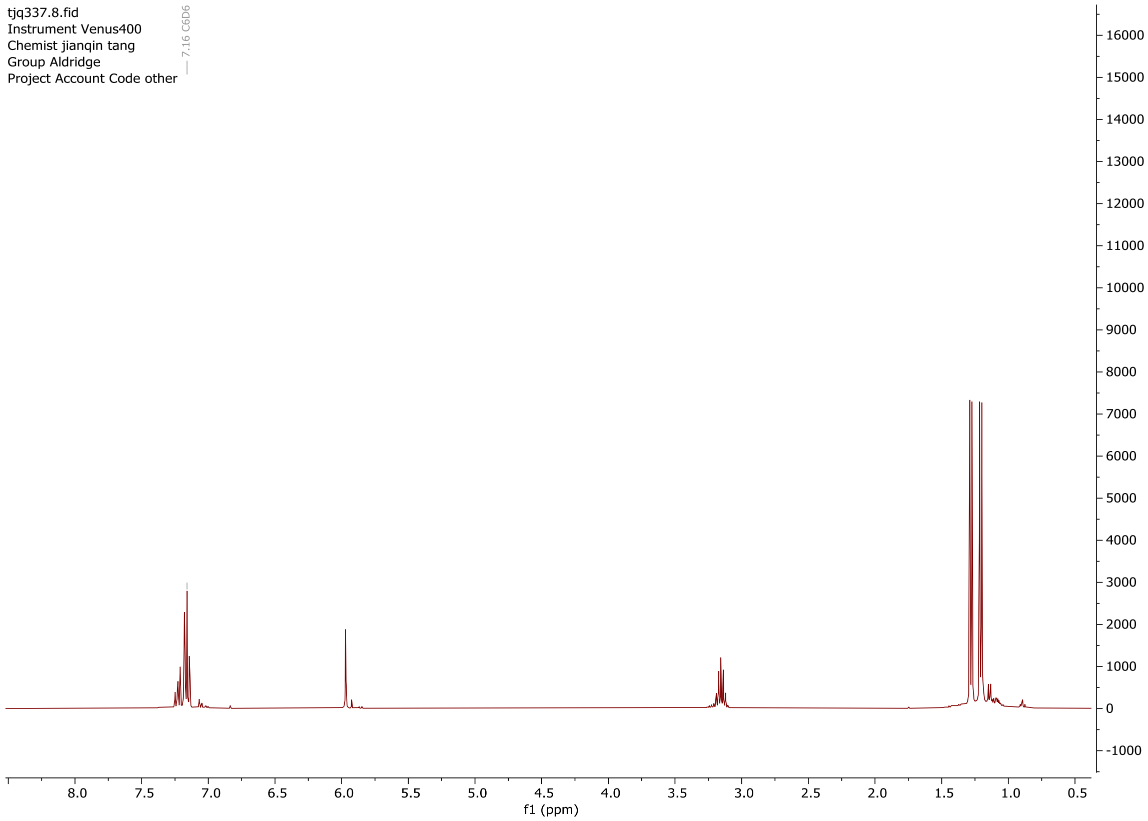


**Figure S20.** $^{1}H$ NMR monitoring of the reaction of **3** with $CO_2$, showing the formation of **5** (400 MHz, $C_6D_6$, 298 K).

## 4. Details of X-ray crystallography

Single-crystal X-ray diffraction data for all compounds were collected on an Oxford Diffraction/Agilent SuperNova diffractometer equipped with a 135 mm Atlas CCD area detector or a Rigaku XtaLAB Synergy-DW VHF equipped with a PhotonJet-R dual wavelength rotating anode and HyPix-Arc 150° detector. Crystals were selected under Paratone-N oil, mounted on MiTeGen Micromount loops and quench-cooled using an Oxford Cryosystems open flow $N_2$ cooling device.[S3] Data were collected at 150 K using mirror monochromated Cu Kα radiation (λ = 1.5418 Å). Data collected were processed using the CrysAlisPro package, including unit cell parameter refinement and inter-frame scaling (which was carried out using SCALE3 ABSPACK within CrysAlisPro).[S4] Equivalent reflections were merged and diffraction patterns processed with the CrysAlisPro suite.[S4] Structures were solved ab initio from the integrated intensities using SHELXT[S5] and refined on $F^2$ using SHELXL[S6] with the graphical interface OLEX2.[S7] Selected crystallographic data are summarised in Tables S1-S3, and full details are given in the supplementary deposited CIF files (2377740-45). These data can be obtained free of charge from the Cambridge Crystallographic Data Centre via http://www.ccdc.cam.ac.uk/data_request/cif.

**Table S1**. Selected crystallographic data and refinement parameters for compounds **5** and **6**.

| compound | 5 | 6 |
|---|---|---|
| Empirical formula | $C_{27}H_{36}BN_3O$ | $C_{27}H_{36}BN_3S$ |
| Fw /g mol$^{-1}$ | 429.4 | 445.46 |
| T/K | 100.00(10) | 100.00(10) |
| Cell setting | monoclinic | monoclinic |
| Space group | $P2_1/c$ | I2/a |
| a/Å | 12.67284(7) | 16.90996(6) |
| b/Å | 9.17962(5) | 9.55705(3) |
| c/Å | 22.43254(13) | 33.35081(10) |
| α/° | 90 | 90 |
| β/° | 96.7556(5) | 94.9584(3) |
| γ/° | 90 | 90 |
| V/Å$^3$ | 2591.50(3) | 5369.63(3) |
| Z | 4 | 8 |
| $\rho_{calc}$g/cm$^3$ | 1.101 | 1.102 |
| μ/mm$^{-1}$ | 0.512 | 1.189 |
| Radiation | Cu Kα (λ = 1.54184) | Cu Kα (λ = 1.54184) |
| Reflections collected | 56620 | 109063 |
| Independent reflections | 5317 [$R_{int}$ = 0.0229] | 5534 [$R_{int}$ = 0.0239] |
| parameters | 297 | 311 |
| GooF | 1.026 | 1.027 |
| Final R indexes [I>=2σ (I)] | $R_1$ = 0.0399, $wR_2$ = 0.1008 | $R_1$ = 0.0353, $wR_2$ = 0.0895 |
| Final R indexes [all data] | $R_1$ = 0.0417, $wR_2$ = 0.1021 | $R_1$ = 0.0359, $wR_2$ = 0.0900 |
| CCDC ref | 2377740 | 2377741 |

**Table S2**. Selected crystallographic data and refinement parameters for compounds **7** and **8**.

| compound | 7 | 8 |
|---|---|---|
| **Empirical formula** | $C_{53}H_{74}BN_5P_2$ | $C_{60}H_{81}B_3N_6O$ |
| **Fw /g mol$^{-1}$** | 853.92 | 934.73 |
| **T/K** | 100.00(10) | 100.00(10) |
| **Cell setting** | monoclinic | monoclinic |
| **Space group** | $P2_1/c$ | C2/c |
| **a/Å** | 12.89680(10) | 22.24980(10) |
| **b/Å** | 19.8442(2) | 13.50310(10) |
| **c/Å** | 40.8371(5) | 37.4373(2) |
| **α/°** | 90 | 90 |
| **β/°** | 96.9990(10) | 99.0810(10) |
| **γ/°** | 90 | 90 |
| **V/Å$^3$** | 10373.42(18) | 11106.73(12) |
| **Z** | 8 | 8 |
| **$\rho_{calc}$g/cm$^3$** | 1.094 | 1.118 |
| **μ/mm$^{-1}$** | 1.04 | 0.498 |
| **Radiation** | Cu Kα (λ = 1.54184) | Cu Kα (λ = 1.54184) |
| **Reflections collected** | 167460 | 67625 |
| **Independent reflections** | 21356 [$R_{int}$ = 0.0625] | 11344 [$R_{int}$ = 0.0205] |
| **parameters** | 1691 | 698 |
| **GooF** | 1.084 | 1.029 |
| **Final R indexes [I>=2σ (I)]** | $R_1$ = 0.0581, $wR_2$ = 0.1446 | $R_1$ = 0.0406, $wR_2$ = 0.1037 |
| **Final R indexes [all data]** | $R_1$ = 0.0767, $wR_2$ = 0.1516 | $R_1$ = 0.0460, $wR_2$ = 0.1074 |
| **CCDC ref** | 2377742 | 2377745 |

**Table S3**. Selected crystallographic data and refinement parameters for compounds **9** and **10**.

| compound | 9 | 10 |
|---|---|---|
| **Empirical formula** | $C_{45}H_{52.4}B_2KN_3O_2$ | $C_{33}H_{42}BN_3$ |
| **Fw /g mol$^{-1}$** | 728.01 | 491.5 |
| **T/K** | 100.00(10) | 100.00(10) |
| **Cell setting** | tetragonal | monoclinic |
| **Space group** | I-4 | $P2_1/n$ |
| **a/Å** | 22.7178(7) | 9.70980(10) |
| **b/Å** | 22.7178(7) | 16.9985(2) |
| **c/Å** | 15.9414(8) | 17.7195(2) |
| **α/°** | 90 | 90 |
| **β/°** | 90 | 93.8430(10) |
| **γ/°** | 90 | 90 |
| **V/Å$^3$** | 8227.3(7) | 2918.06(6) |
| **Z** | 8 | 4 |
| **$\rho_{calc}$g/cm$^3$** | 1.175 | 1.119 |
| **μ/mm$^{-1}$** | 1.428 | 0.487 |
| **Radiation** | Cu Kα (λ = 1.54184) | Cu Kα (λ = 1.54184) |
| **Reflections collected** | 30711 | 24546 |
| **Independent reflections** | 8001 [$R_{int}$ = 0.0416] | 24546 |
| **parameters** | 527 | 345 |
| **GooF** | 1.016 | 1.055 |
| **Final R indexes [I>=2σ (I)]** | $R_1$ = 0.0551, $wR_2$ = 0.1556 | $R_1$ = 0.0501, $wR_2$ = 0.1745 |
| **Final R indexes [all data]** | $R_1$ = 0.0595, $wR_2$ = 0.1600 | $R_1$ = 0.0541, $wR_2$ = 0.1774 |
| **CCDC ref** | 2377743 | 2377744 |

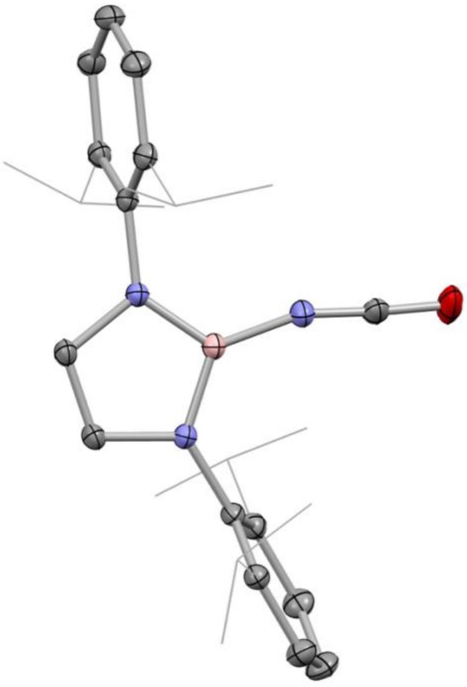

**Figure S16**. Molecular structure of **5** in the solid state as determined by X-ray crystallography. All Hs omitted and $^{i}$Pr groups shown in wireframe format for clarity; thermal ellipsoids shown at the 35% probability level.

## 5. Details of quantum chemical calculations

Geometry optimizations were carried out using the Gaussian 16 package with the M06-2X functional.[S8,S9] The def2-SVP basis set was used for all atoms.[S10,S11] Frequency calculations at same level of theory were performed to identify the number of imaginary frequencies (zero for local minimum and one for transition states), and provide the thermal corrections of Gibbs free energy. Single-point energy calculations were performed at the M06-2X/def2-TZVP level of theory for the modelling of compounds in solution (benzene).[S12,S13] The gas-phase geometry was used for all the solution phase calculations. The SMD method was used with the corresponding solvent, while Bondi radii were chosen as the atomic radii to define the molecular cavity.[S14,S15] The corrections of Gibbs free energy from frequency calculations were added to the single-point energies to obtain the Gibbs free energy in solution. All energies reported in the paper correspond to the reference state of 1 mol/L, 298 K.

**Table S6.** Calculated energies of intermediates and transition states.

| Species | Thermal Corrections of Gibbs Free Energies (Hartree) | Solvation Energies (Hartree) |
|---|---|---|
| **1** (anionic component) | 1.061871 | -2428.25269 |
| PhCHO | 0.080626 | -345.564694 |
| (boryl)N=C(H)Ph | 0.615977 | -1471.941512 |
| IN3 | 0.632052 | -1647.489932 |
| IN2 | 0.522271 | -1301.886084 |
| BO2 | -0.008553 | -175.532068 |
| IN1 | 1.173277 | -2773.848201 |
| TS1 | 1.169813 | -2773.797297 |
| TS2 | 1.169729 | -2773.805785 |
| TS3 | 0.629418 | -1647.439945 |
| TS4 | 0.626958 | -1647.444168 |

Coordinates for optimised geometries

**PhCHO**

| | | | |
|---|---|---|---|
| C | 3.43481100 | 1.01852900 | 0.00937100 |
| C | 4.82327200 | 1.02236600 | -0.14245000 |
| C | 5.51885600 | 2.22997400 | -0.15353600 |
| C | 4.82160500 | 3.42964200 | -0.01229200 |
| C | 3.43166300 | 3.42750800 | 0.14005300 |
| C | 2.73709000 | 2.22344900 | 0.15091800 |
| H | 5.35663000 | 0.07452100 | -0.25152700 |
| H | 6.60307600 | 2.23792600 | -0.27216800 |
| H | 5.36456300 | 4.37618600 | -0.02104400 |
| H | 2.89460200 | 4.37063000 | 0.25021700 |
| H | 1.65259700 | 2.18442400 | 0.26754100 |
| C | 2.69222400 | -0.26919900 | 0.02065100 |
| O | 1.50054700 | -0.36040800 | 0.14595600 |
| H | 3.33093100 | -1.17893200 | -0.09929800 |

**1** (anionic component only)

| | | | |
|---|---|---|---|
| N | 3.76389000 | 0.76964700 | 0.20547100 |
| N | -3.61635700 | -0.76975400 | 0.51321800 |
| N | 2.42913000 | 2.44059900 | -0.62093100 |
| N | 1.35455300 | 0.10758500 | -0.38722900 |
| N | -1.25591700 | -0.19916600 | -0.29940400 |
| N | -2.37801300 | -2.52448100 | -0.28650300 |
| C | 4.15619600 | -0.31657700 | 1.02686800 |
| C | 4.47803400 | 1.97194400 | 0.17631700 |
| H | 5.50816000 | 2.03144500 | 0.52131400 |
| C | 0.25363600 | 3.50211800 | -0.29852100 |
| C | -4.03469200 | 0.47671900 | 1.03434200 |
| C | 3.69485900 | 2.95378600 | -0.33094900 |
| H | 3.94446800 | 3.99734100 | -0.51460200 |
| C | -4.34455000 | 1.52965900 | 0.14699200 |

| | | | |
|---|---|---|---|
| C | -4.36051400 | -1.94877000 | 0.60495300 |
| H | -5.37058400 | -1.95795900 | 1.00993500 |
| C | -1.61950900 | -3.77209200 | -2.24228200 |
| C | 4.85660700 | -1.40413800 | 0.46731000 |
| C | -1.40075400 | -3.32964600 | -0.92011800 |
| C | -4.24034700 | 1.30923100 | -1.35409400 |
| H | -3.29004400 | 0.77817600 | -1.51771300 |
| C | -0.19938600 | -3.62641700 | -0.24255400 |
| C | -3.63274300 | -2.98304100 | 0.11825400 |
| H | -3.91413500 | -4.03090300 | 0.03165200 |
| C | -4.07099100 | 0.66672800 | 2.43157600 |
| C | 0.02287600 | -3.14034100 | 1.18127000 |
| H | -0.37771100 | -2.11734400 | 1.23654900 |
| C | -2.82221500 | -3.26074400 | -3.01971000 |
| H | -3.67294500 | -3.19304500 | -2.32618700 |
| C | 1.38159300 | 3.62635800 | -2.47841800 |
| C | 1.35560100 | 3.20987000 | -1.13103500 |
| C | 0.24309600 | 3.05053200 | 1.15386500 |
| H | 0.66068400 | 2.03376300 | 1.17387200 |
| C | -0.65010900 | -4.57497300 | -2.84990800 |
| H | -0.80471500 | -4.93503700 | -3.86823600 |
| B | 0.04750900 | -0.04373200 | -0.38128600 |
| C | -4.82109000 | 2.94357000 | 2.06498000 |
| H | -5.13201300 | 3.90991200 | 2.46712000 |
| C | -3.53493800 | -0.41902100 | 3.35119300 |
| H | -3.79189200 | -1.39211300 | 2.90924700 |
| C | -4.73585100 | 2.75877400 | 0.68743400 |
| H | -4.97288400 | 3.58995800 | 0.02230500 |
| C | 3.81135400 | -0.30033400 | 2.39303900 |
| C | -0.80355100 | 4.24459500 | -0.83473100 |
| H | -1.67405700 | 4.46357800 | -0.21439800 |
| C | -4.48523900 | 1.90601000 | 2.92924800 |

| | | | |
|---|---|---|---|
| H | -4.52391400 | 2.07133000 | 4.00719200 |
| B | -2.29611400 | -1.07401100 | -0.05462100 |
| C | 5.22476000 | -2.46357200 | 1.30100700 |
| H | 5.76458800 | -3.31608200 | 0.88510200 |
| C | 0.52898200 | -4.89452500 | -2.18313500 |
| H | 1.28536600 | -5.50936700 | -2.67508300 |
| C | 2.49133300 | 3.13395700 | -3.39347900 |
| H | 3.42696400 | 3.12895900 | -2.81527400 |
| C | 0.31841100 | 4.39259300 | -2.96460800 |
| H | 0.32411800 | 4.73323400 | -4.00111400 |
| C | -1.15608300 | 2.96651600 | 1.75811100 |
| H | -1.62772500 | 3.95782000 | 1.85744500 |
| H | -1.09618900 | 2.53517300 | 2.76878700 |
| H | -1.81026800 | 2.31782500 | 1.15499200 |
| C | -0.76617000 | 4.70185600 | -2.14892300 |
| H | -1.59880800 | 5.28626000 | -2.54629400 |
| C | -2.52424300 | -1.83992200 | -3.52058800 |
| H | -1.70069500 | -1.86549900 | -4.25176900 |
| H | -2.21388200 | -1.18254900 | -2.69479900 |
| H | -3.40919200 | -1.40366200 | -4.00983100 |
| C | 1.49448300 | -3.06599100 | 1.58034800 |
| H | 1.58419700 | -2.61710400 | 2.58035600 |
| H | 2.06426900 | -2.43284100 | 0.88141100 |
| H | 1.96390800 | -4.06221200 | 1.63033800 |
| C | 0.75605700 | -4.40897200 | -0.89910800 |
| H | 1.69606000 | -4.64031700 | -0.39751700 |
| C | -5.38811600 | 0.42769100 | -1.86171200 |
| H | -5.39332500 | -0.55283500 | -1.36600400 |
| H | -5.29263100 | 0.26196900 | -2.94597100 |
| H | -6.35760200 | 0.91832000 | -1.67582000 |
| C | 2.96489000 | 0.81979100 | 2.97671400 |
| H | 2.91178100 | 1.62512200 | 2.22958000 |

| | | | |
|---|---|---|---|
| B | 2.39935800 | 1.00736800 | -0.28825000 |
| C | 4.20040100 | -1.37897100 | 3.19425100 |
| H | 3.92972000 | -1.38653000 | 4.25304800 |
| C | -0.77295400 | -4.00788900 | 2.16446400 |
| H | -0.42689500 | -5.05343600 | 2.12009100 |
| H | -1.84815500 | -3.98772800 | 1.93353800 |
| H | -0.63513400 | -3.64593200 | 3.19528200 |
| C | 4.90489200 | -2.45092900 | 2.65694200 |
| H | 5.19636600 | -3.28837300 | 3.29405500 |
| C | -4.17518600 | 2.60646700 | -2.15646900 |
| H | -5.12538300 | 3.16364100 | -2.11269200 |
| H | -3.97559600 | 2.37725700 | -3.21348200 |
| H | -3.36621400 | 3.25850500 | -1.79765100 |
| C | 5.10895200 | -1.44184200 | -1.02871600 |
| H | 5.23238800 | -0.39899700 | -1.35725600 |
| C | 1.15356500 | 3.95003500 | 1.99969400 |
| H | 2.18207700 | 3.96305900 | 1.61024300 |
| H | 1.18670100 | 3.59061100 | 3.04108800 |
| H | 0.77145200 | 4.98386800 | 2.00763900 |
| C | -3.24014400 | -4.16489600 | -4.17811900 |
| H | -4.18113700 | -3.80163400 | -4.61730600 |
| H | -3.39104000 | -5.20416300 | -3.84949100 |
| H | -2.48720500 | -4.16736200 | -4.98145200 |
| C | 2.71083100 | 4.01090500 | -4.62482500 |
| H | 1.85729600 | 3.95424700 | -5.31806400 |
| H | 3.59930200 | 3.66906600 | -5.17624600 |
| H | 2.85869400 | 5.06673000 | -4.35232300 |
| C | -2.00223000 | -0.32776600 | 3.39652700 |
| H | -1.57361500 | -0.27141600 | 2.38357300 |
| H | -1.57049700 | -1.19751400 | 3.91628900 |
| H | -1.69671800 | 0.58167700 | 3.93876200 |
| C | 3.87149700 | -2.00735500 | -1.73916700 |

| | | | |
|---|---|---|---|
| H | 3.99908900 | -1.97291300 | -2.83272600 |
| H | 2.96319400 | -1.44660100 | -1.46958500 |
| H | 3.71727700 | -3.05860600 | -1.44517000 |
| C | 6.36643800 | -2.21819600 | -1.41746800 |
| H | 6.25001000 | -3.29686000 | -1.22881200 |
| H | 7.25018500 | -1.86838700 | -0.86282800 |
| H | 6.56400000 | -2.09909600 | -2.49318600 |
| C | 3.57649600 | 1.41409500 | 4.24685500 |
| H | 2.97190900 | 2.26431200 | 4.59770200 |
| H | 4.60028400 | 1.77227200 | 4.06357400 |
| H | 3.61396300 | 0.67606000 | 5.06359500 |
| C | -4.12206700 | -0.38094300 | 4.76101100 |
| H | -3.79527100 | 0.51451100 | 5.31205200 |
| H | -3.77976300 | -1.25556100 | 5.33375000 |
| H | -5.22233400 | -0.38833500 | 4.74402600 |
| C | 1.53320700 | 0.32641000 | 3.21950000 |
| H | 1.51408800 | -0.46195500 | 3.99080800 |
| H | 1.10644300 | -0.08243300 | 2.29052300 |
| H | 0.89037800 | 1.15338800 | 3.56193900 |
| C | 2.19855800 | 1.68362800 | -3.80473800 |
| H | 2.03131000 | 1.04450000 | -2.92563000 |
| H | 3.03319400 | 1.26760900 | -4.39058900 |
| H | 1.28792500 | 1.64619500 | -4.42339800 |

**TS1**

| | | | |
|---|---|---|---|
| N | 0.69781500 | 1.96236200 | 7.85609900 |
| N | 1.96108300 | 3.12576500 | 6.34144500 |
| N | 3.98918600 | 7.25041900 | 11.28599500 |
| N | 4.33317500 | 5.40005900 | 12.59312400 |
| N | 2.01081500 | 3.97511600 | 8.77182500 |
| N | 4.12117000 | 4.93832100 | 10.06357700 |
| C | 0.31027500 | 1.30296200 | 9.05104400 |

| | | | |
|---|---|---|---|
| C | -1.02617400 | 1.39551700 | 9.48796400 |
| C | 4.18246500 | 4.09525300 | 13.14712100 |
| C | 3.07245000 | 3.82814500 | 5.81233400 |
| C | 3.44885300 | 8.17931700 | 10.34871600 |
| C | 0.55292000 | 1.39159600 | 6.58567700 |
| H | -0.09683200 | 0.53517200 | 6.41694600 |
| C | 4.27334600 | 8.79225500 | 9.38717700 |
| C | 1.29944300 | 2.08218700 | 5.69212100 |
| H | 1.39170800 | 1.92612800 | 4.61993000 |
| C | -1.39610600 | 0.72998000 | 10.65997100 |
| H | -2.42678300 | 0.78454200 | 11.01415600 |
| C | 4.14759600 | 7.63729600 | 12.62095700 |
| H | 4.10480800 | 8.68473700 | 12.91043600 |
| C | 5.28945800 | 3.42980500 | 13.70613800 |
| C | 4.25856200 | 3.13031400 | 5.48991500 |
| C | 1.28095200 | 0.59603500 | 9.79364100 |
| C | 3.00193900 | 5.23043800 | 5.68738700 |
| C | -1.99690300 | 2.28090600 | 8.72389200 |
| H | -1.75941900 | 2.17641200 | 7.65471200 |
| C | -0.45715400 | 0.00402200 | 11.38934700 |
| H | -0.75813400 | -0.50942200 | 12.30516500 |
| C | 4.35661400 | 6.54591500 | 13.39226100 |
| H | 4.52473900 | 6.48759700 | 14.46569400 |
| C | 1.56489700 | 9.48810700 | 9.58828600 |
| H | 0.50368300 | 9.74215800 | 9.64435400 |
| C | 2.90262900 | 3.49140800 | 13.13052400 |
| C | 2.07770700 | 8.50357000 | 10.43723500 |
| C | 0.86641600 | -0.05233900 | 10.96361900 |
| H | 1.60107700 | -0.59965300 | 11.55661200 |
| C | 3.71781300 | 9.77139000 | 8.55693000 |
| H | 4.34900900 | 10.24842000 | 7.80374700 |
| C | 1.73099400 | 5.99164700 | 6.02545400 |

| | | | |
|---|---|---|---|
| H | 1.09666000 | 5.32681600 | 6.62331400 |
| C | 5.27392600 | 5.23888300 | 4.82937700 |
| H | 6.13577900 | 5.79093200 | 4.44917400 |
| C | 5.33747600 | 3.85700300 | 4.97772700 |
| H | 6.25812400 | 3.32970200 | 4.71626900 |
| C | 2.37949700 | 10.13162300 | 8.66235400 |
| H | 1.96346900 | 10.89692100 | 8.00423800 |
| C | 1.67277900 | 4.25075900 | 12.65423800 |
| H | 1.97328400 | 4.89841600 | 11.81826500 |
| C | 4.11399100 | 5.91609700 | 5.19025300 |
| H | 4.06936800 | 7.00243200 | 5.08721400 |
| C | 4.38461000 | 1.62380000 | 5.67135500 |
| H | 3.53894600 | 1.29437500 | 6.29101600 |
| C | 5.10747200 | 2.14392300 | 14.22720000 |
| H | 5.96270500 | 1.61560700 | 14.65554800 |
| C | 5.70191900 | 8.34277800 | 9.17278700 |
| H | 5.97749700 | 7.70248500 | 10.02019700 |
| C | 2.77211100 | 2.19965200 | 13.64744500 |
| H | 1.79997300 | 1.70590500 | 13.61272700 |
| C | 6.66627100 | 4.06916700 | 13.76243100 |
| H | 6.59659800 | 5.04405300 | 13.26183400 |
| C | 3.86382900 | 1.52678700 | 14.19044600 |
| H | 3.74198800 | 0.51460600 | 14.58054600 |
| C | 1.13937800 | 7.76153600 | 11.37456500 |
| H | 1.74906200 | 7.12595900 | 12.02999300 |
| C | 2.72889600 | 0.52420200 | 9.33455300 |
| H | 2.98594900 | 1.46820000 | 8.83317800 |
| C | -3.46298800 | 1.89981300 | 8.91916200 |
| H | -3.79731200 | 2.09786600 | 9.94942600 |
| H | -3.63896400 | 0.83550400 | 8.70287200 |
| H | -4.10002500 | 2.49808800 | 8.25091400 |
| C | 6.69984500 | 9.50005900 | 9.11632900 |

| | | | |
|---|---|---|---|
| H | 6.53166100 | 10.14067800 | 8.23611900 |
| H | 7.72275500 | 9.10043400 | 9.04175600 |
| H | 6.63754300 | 10.13222000 | 10.01476800 |
| C | 2.91347100 | -0.61906800 | 8.32902600 |
| H | 3.95414100 | -0.63875600 | 7.97179500 |
| H | 2.25328700 | -0.49818700 | 7.45694200 |
| H | 2.68944100 | -1.59075700 | 8.80017800 |
| C | 1.15099000 | 5.14739700 | 13.78597900 |
| H | 0.85243800 | 4.52973100 | 14.64811000 |
| H | 0.26842800 | 5.71380500 | 13.44898700 |
| H | 1.91226800 | 5.86633400 | 14.12147900 |
| C | 0.55805800 | 3.35449800 | 12.12535900 |
| H | 0.92716200 | 2.67792100 | 11.34300000 |
| H | -0.22939100 | 3.97672700 | 11.67669100 |
| H | 0.09123200 | 2.75507700 | 12.92315100 |
| C | 1.99833600 | 7.23591300 | 6.87011300 |
| H | 2.58450000 | 7.99296000 | 6.32457300 |
| H | 2.54838800 | 6.98140500 | 7.79017800 |
| H | 1.04952700 | 7.70981100 | 7.16563900 |
| C | 0.97060900 | 6.33919000 | 4.74080700 |
| H | 0.02877400 | 6.86080100 | 4.97233700 |
| H | 0.73196400 | 5.43008400 | 4.16915400 |
| H | 1.57651400 | 6.99586700 | 4.09572900 |
| B | 3.05778100 | 4.44728200 | 9.35460000 |
| C | 3.73077200 | 0.41322500 | 10.48171700 |
| H | 3.68977900 | -0.56683000 | 10.98774200 |
| H | 3.55975300 | 1.20410400 | 11.22752800 |
| H | 4.74053500 | 0.57075800 | 10.07971500 |
| C | 0.34689600 | 8.71076300 | 12.27577400 |
| H | 1.01971600 | 9.35973600 | 12.85573700 |
| H | -0.27172000 | 8.13755600 | 12.98302800 |
| H | -0.32835200 | 9.35524200 | 11.69150900 |

| | | | |
|---|---|---|---|
| B | 4.12734100 | 5.80353600 | 11.20466500 |
| C | 5.76878600 | 7.48831300 | 7.90318200 |
| H | 5.09804100 | 6.61893300 | 7.97692100 |
| H | 6.78976500 | 7.11426000 | 7.74008400 |
| H | 5.46757900 | 8.08263300 | 7.02367300 |
| B | 1.59210500 | 3.10809200 | 7.75583900 |
| C | 0.21212100 | 6.84171900 | 10.57104800 |
| H | -0.42293900 | 7.42575100 | 9.88502500 |
| H | -0.45037300 | 6.27649400 | 11.24597400 |
| H | 0.78293200 | 6.11659200 | 9.96907900 |
| C | 7.09296000 | 4.32494500 | 15.21212200 |
| H | 7.18021900 | 3.37834600 | 15.76845700 |
| H | 6.36178900 | 4.95569900 | 15.73880400 |
| H | 8.07164400 | 4.82763400 | 15.24524400 |
| C | 7.71518200 | 3.23374600 | 13.02263200 |
| H | 8.68012900 | 3.76290000 | 12.99846000 |
| H | 7.40933400 | 3.03950500 | 11.98512100 |
| H | 7.87253500 | 2.26444500 | 13.52158300 |
| C | 4.29847700 | 0.90700500 | 4.31834600 |
| H | 4.31691900 | -0.18524100 | 4.45735900 |
| H | 3.38100300 | 1.16943700 | 3.77240000 |
| H | 5.15549600 | 1.18386100 | 3.68269700 |
| C | 5.65901200 | 1.22001800 | 6.41739800 |
| H | 5.65989600 | 1.66470000 | 7.42422800 |
| H | 5.70235800 | 0.12261100 | 6.50687700 |
| H | 6.56667400 | 1.53349000 | 5.87577100 |
| C | -1.76209800 | 3.74995500 | 9.10212900 |
| H | -0.70167900 | 4.02405500 | 8.99498700 |
| H | -2.36659200 | 4.41851600 | 8.46938200 |
| H | -2.05180900 | 3.91706700 | 10.15223700 |
| O | 4.93287300 | 2.87014900 | 9.01980600 |
| C | 5.34086700 | 3.74868200 | 9.84684200 |

| | | | |
|---|---|---|---|
| C | 6.59010200 | 4.53997300 | 9.43508200 |
| C | 7.13821200 | 4.29829200 | 8.17343300 |
| C | 7.24854400 | 5.42000800 | 10.29840700 |
| C | 8.32845300 | 4.91242100 | 7.78595500 |
| H | 6.60000100 | 3.61179000 | 7.51883500 |
| C | 8.43670000 | 6.04361100 | 9.91379300 |
| H | 6.82341700 | 5.62017400 | 11.28688400 |
| C | 8.98501700 | 5.78536400 | 8.65589500 |
| H | 8.74561500 | 4.71178700 | 6.79620000 |
| H | 8.93839600 | 6.73018900 | 10.59975700 |
| H | 9.91917700 | 6.26506600 | 8.35586400 |
| H | 5.45309400 | 3.44771400 | 10.92135500 |

**IN1**

| | | | |
|---|---|---|---|
| N | 0.59398700 | 2.04417900 | 7.84671200 |
| N | 2.06410300 | 3.05240300 | 6.39990800 |
| N | 3.79144700 | 7.29941000 | 11.36469900 |
| N | 4.32341100 | 5.47021000 | 12.62823400 |
| N | 2.02964000 | 3.96186000 | 8.83652200 |
| N | 4.11983100 | 5.03271600 | 10.10034700 |
| C | 0.17969500 | 1.34788500 | 9.00718100 |
| C | -1.12750300 | 1.53640700 | 9.50222700 |
| C | 4.28565100 | 4.14017600 | 13.13273900 |
| C | 3.18720200 | 3.70527200 | 5.85229400 |
| C | 3.28378300 | 8.23818200 | 10.42223300 |
| C | 0.52238900 | 1.42892000 | 6.59077200 |
| H | -0.17657000 | 0.61795100 | 6.39326100 |
| C | 4.14635000 | 8.86852300 | 9.50896300 |
| C | 1.37862100 | 2.03341500 | 5.73728200 |
| H | 1.53026000 | 1.84111600 | 4.67649500 |
| C | -1.52785800 | 0.82174600 | 10.63336900 |
| H | -2.53543500 | 0.95416500 | 11.03122400 |

| | | | |
|---|---|---|---|
| C | 3.95181500 | 7.67921700 | 12.70110100 |
| H | 3.82734200 | 8.71527400 | 13.00728800 |
| C | 5.44776300 | 3.54868500 | 13.66123000 |
| C | 4.32152800 | 2.95980300 | 5.45349000 |
| C | 1.07977700 | 0.47250700 | 9.64712300 |
| C | 3.18015500 | 5.11258200 | 5.73009200 |
| C | -2.02911600 | 2.55005000 | 8.81910300 |
| H | -1.82839900 | 2.47195200 | 7.73998500 |
| C | -0.65364600 | -0.06397400 | 11.26275900 |
| H | -0.98147400 | -0.61898500 | 12.14432300 |
| C | 4.27110300 | 6.59870100 | 13.44975900 |
| H | 4.45793700 | 6.53698500 | 14.51979600 |
| C | 1.42768800 | 9.55593000 | 9.62058700 |
| H | 0.36588900 | 9.80965600 | 9.63849900 |
| C | 3.06402400 | 3.42610700 | 13.07035500 |
| C | 1.90796700 | 8.55144100 | 10.46376600 |
| C | 0.63669300 | -0.23270000 | 10.77311400 |
| H | 1.32182100 | -0.91855200 | 11.27788200 |
| C | 3.62141400 | 9.86654100 | 8.67976200 |
| H | 4.28002700 | 10.36336400 | 7.96317000 |
| C | 1.96117700 | 5.93949100 | 6.10327600 |
| H | 1.23398600 | 5.26574100 | 6.57059500 |
| C | 5.40444300 | 5.02068400 | 4.75001900 |
| H | 6.26705900 | 5.53454600 | 4.32095800 |
| C | 5.40210100 | 3.63473000 | 4.87622700 |
| H | 6.27725400 | 3.06184200 | 4.55624700 |
| C | 2.27966700 | 10.22204500 | 8.74305100 |
| H | 1.88881500 | 11.00418900 | 8.08931900 |
| C | 1.77829700 | 4.09710600 | 12.60553500 |
| H | 2.01503500 | 4.72843900 | 11.73706800 |
| C | 4.30391000 | 5.74813400 | 5.19246900 |
| H | 4.30596000 | 6.83768700 | 5.10487400 |

| | | | |
|---|---|---|---|
| C | 4.43009100 | 1.45788200 | 5.68527500 |
| H | 3.50751600 | 1.12167200 | 6.17578900 |
| C | 5.38095300 | 2.22754800 | 14.11907000 |
| H | 6.27738900 | 1.75664400 | 14.52973200 |
| C | 5.59357800 | 8.44472300 | 9.35587000 |
| H | 5.80902700 | 7.71121400 | 10.14441400 |
| C | 3.05246000 | 2.10177600 | 13.51589100 |
| H | 2.13166400 | 1.52182300 | 13.44221300 |
| C | 6.76272500 | 4.30447000 | 13.74245300 |
| H | 6.61983800 | 5.26450400 | 13.22636600 |
| C | 4.19839600 | 1.50428200 | 14.03691400 |
| H | 4.16597500 | 0.46693400 | 14.37528100 |
| C | 0.95210100 | 7.74391400 | 11.32519400 |
| H | 1.54061700 | 7.25348700 | 12.11274700 |
| C | 2.50488500 | 0.29018000 | 9.14920200 |
| H | 2.69623500 | 1.05692600 | 8.38513400 |
| C | -3.51844000 | 2.30008300 | 9.04631400 |
| H | -3.80019900 | 2.47361700 | 10.09679100 |
| H | -3.80485200 | 1.27079200 | 8.78257700 |
| H | -4.11420600 | 2.99228100 | 8.43280000 |
| C | 6.56968700 | 9.61093800 | 9.52276100 |
| H | 6.43782900 | 10.36406800 | 8.72966600 |
| H | 7.60584200 | 9.24376500 | 9.46301100 |
| H | 6.43365400 | 10.11212400 | 10.49269600 |
| C | 2.68071700 | -1.07923800 | 8.48495800 |
| H | 3.70578000 | -1.18888100 | 8.09807900 |
| H | 1.98059700 | -1.19843000 | 7.64467900 |
| H | 2.49798400 | -1.89485800 | 9.20402600 |
| C | 1.22033100 | 4.98920700 | 13.72391400 |
| H | 0.96215600 | 4.37521100 | 14.60194100 |
| H | 0.30426400 | 5.49603300 | 13.38122500 |
| H | 1.93916800 | 5.75854200 | 14.03841300 |

| | | | |
|---|---|---|---|
| C | 0.71000300 | 3.11991900 | 12.13147400 |
| H | 1.09871300 | 2.44983100 | 11.35363100 |
| H | -0.12113900 | 3.68113900 | 11.68206100 |
| H | 0.29878300 | 2.51705200 | 12.95762600 |
| C | 2.29555800 | 7.02265800 | 7.12638400 |
| H | 3.01045700 | 7.75906400 | 6.72450300 |
| H | 2.72767800 | 6.57195500 | 8.03252400 |
| H | 1.38695300 | 7.57088900 | 7.42055100 |
| C | 1.31784200 | 6.53488100 | 4.84572800 |
| H | 0.40652300 | 7.09494500 | 5.10689400 |
| H | 1.04589800 | 5.74575600 | 4.12888300 |
| H | 2.00758400 | 7.22923200 | 4.33915200 |
| B | 3.32080500 | 4.11585400 | 9.21857200 |
| C | 3.52895900 | 0.51486700 | 10.26351300 |
| H | 3.44648600 | -0.24701100 | 11.05651900 |
| H | 3.39729700 | 1.50841600 | 10.71668700 |
| H | 4.54847400 | 0.47495000 | 9.85213400 |
| C | -0.10850800 | 8.60091100 | 12.01722300 |
| H | 0.34689700 | 9.42147600 | 12.59181400 |
| H | -0.69792200 | 7.98071400 | 12.70938500 |
| H | -0.81399000 | 9.03942400 | 11.29450700 |
| B | 4.04618200 | 5.87540900 | 11.25320900 |
| C | 5.78653500 | 7.74088900 | 8.00958600 |
| H | 5.11827600 | 6.87142600 | 7.92628600 |
| H | 6.82210400 | 7.38560700 | 7.90311900 |
| H | 5.56646700 | 8.42772500 | 7.17444100 |
| B | 1.60904300 | 3.12154100 | 7.81447100 |
| C | 0.30781800 | 6.63944800 | 10.47722800 |
| H | -0.31661800 | 7.08047500 | 9.68195500 |
| H | -0.34159600 | 6.00517300 | 11.10235500 |
| H | 1.05001600 | 5.98322800 | 9.99104500 |
| C | 7.13062400 | 4.61258200 | 15.19800800 |

| | | | |
|---|---|---|---|
| H | 7.27325300 | 3.68146700 | 15.76897300 |
| H | 6.33909700 | 5.19134300 | 15.69560200 |
| H | 8.06648200 | 5.18986400 | 15.25020300 |
| C | 7.89801400 | 3.55167200 | 13.04198900 |
| H | 8.80826100 | 4.17017000 | 13.00892000 |
| H | 7.62787900 | 3.29203300 | 12.00846500 |
| H | 8.14514800 | 2.61999800 | 13.57446300 |
| C | 4.55964400 | 0.69060300 | 4.36630700 |
| H | 4.59483800 | -0.39423800 | 4.55120200 |
| H | 3.71077700 | 0.89870200 | 3.69812000 |
| H | 5.48218200 | 0.97098400 | 3.83295800 |
| C | 5.58299600 | 1.12813500 | 6.63946800 |
| H | 5.42880200 | 1.63590400 | 7.60261500 |
| H | 5.63589800 | 0.04106800 | 6.81197000 |
| H | 6.55383600 | 1.44772300 | 6.22579500 |
| C | -1.64077700 | 3.97019300 | 9.25130800 |
| H | -0.55792900 | 4.14233100 | 9.14275100 |
| H | -2.18198900 | 4.72130800 | 8.65425500 |
| H | -1.90831100 | 4.12341400 | 10.31000900 |
| O | 4.48721000 | 3.28048400 | 8.97249900 |
| C | 5.20440000 | 4.06823400 | 9.88832500 |
| C | 6.48559000 | 4.64340600 | 9.32026200 |
| C | 6.83975200 | 4.40601200 | 7.99187800 |
| C | 7.33190100 | 5.41316800 | 10.12564000 |
| C | 8.03829500 | 4.90741900 | 7.48243200 |
| H | 6.15627200 | 3.83046800 | 7.36718200 |
| C | 8.52694800 | 5.92025100 | 9.61712600 |
| H | 7.04683900 | 5.61476100 | 11.16255000 |
| C | 8.88893900 | 5.65860300 | 8.29329500 |
| H | 8.30044000 | 4.71312200 | 6.43992400 |
| H | 9.17952600 | 6.51912200 | 10.25602100 |
| H | 9.82767100 | 6.04891500 | 7.89515100 |

| | | | |
|---|---|---|---|
| H | 5.45136900 | 3.48991300 | 10.80522200 |

**TS2**

| | | | |
|---|---|---|---|
| N | 0.45559600 | 1.81853700 | 7.55503600 |
| N | 1.68129700 | 3.22460500 | 6.22390100 |
| N | 3.46708900 | 7.32044500 | 11.31034800 |
| N | 4.03782900 | 5.67246400 | 12.81358600 |
| N | 2.28433700 | 3.16303200 | 8.72341100 |
| N | 4.38458100 | 5.15085400 | 10.27386600 |
| C | -0.02730300 | 1.02878400 | 8.62992800 |
| C | -1.28629900 | 1.32421300 | 9.19004600 |
| C | 4.39390500 | 4.47014000 | 13.46820200 |
| C | 2.82131300 | 3.87180500 | 5.67195400 |
| C | 3.10499500 | 8.11574500 | 10.19767900 |
| C | 0.02301100 | 1.70243100 | 6.22966200 |
| H | -0.79224300 | 1.03760100 | 5.95146500 |
| C | 4.09445700 | 8.54486500 | 9.28452400 |
| C | 0.76002200 | 2.52249500 | 5.44165200 |
| H | 0.69533200 | 2.67431300 | 4.36636300 |
| C | -1.75229800 | 0.53796400 | 10.24801700 |
| H | -2.72655800 | 0.74831600 | 10.69157700 |
| C | 3.28178900 | 7.77915600 | 12.61830200 |
| H | 2.93508700 | 8.79045200 | 12.82074300 |
| C | 5.60464700 | 4.40631400 | 14.19308600 |
| C | 3.98695800 | 3.11739500 | 5.43853000 |
| C | 0.78088100 | -0.00369700 | 9.15358900 |
| C | 2.77581400 | 5.25154300 | 5.38710900 |
| C | -2.04346300 | 2.55544400 | 8.72351300 |
| H | -1.87990900 | 2.65986900 | 7.64094200 |
| C | -0.97575200 | -0.49691500 | 10.75944700 |
| H | -1.34624500 | -1.09700900 | 11.59275800 |
| C | 3.62543700 | 6.81654200 | 13.50212500 |

| | | | |
|---|---|---|---|
| H | 3.59593700 | 6.85011600 | 14.58968200 |
| C | 1.42237900 | 9.35884500 | 8.98127900 |
| H | 0.38708700 | 9.67233400 | 8.84227700 |
| C | 3.55675100 | 3.33848500 | 13.34362500 |
| C | 1.75186100 | 8.49532500 | 10.03062000 |
| C | 0.28277400 | -0.75391000 | 10.22383000 |
| H | 0.89154000 | -1.55043200 | 10.65256800 |
| C | 3.70994700 | 9.37982400 | 8.22959200 |
| H | 4.45900900 | 9.71765300 | 7.51231400 |
| C | 1.53666600 | 6.05063400 | 5.74161700 |
| H | 0.66763000 | 5.40979200 | 5.52257900 |
| C | 5.06400900 | 5.11847800 | 4.58872100 |
| H | 5.94224000 | 5.60881100 | 4.16285300 |
| C | 5.10131800 | 3.76287400 | 4.89158900 |
| H | 6.01829600 | 3.19360600 | 4.71979600 |
| C | 2.39120700 | 9.80146900 | 8.08635600 |
| H | 2.11451100 | 10.46501800 | 7.26442000 |
| C | 2.23506400 | 3.43244700 | 12.59518500 |
| H | 2.40949700 | 4.05715700 | 11.70677700 |
| C | 3.91052100 | 5.85794500 | 4.84245200 |
| H | 3.89647000 | 6.92493900 | 4.61648400 |
| C | 4.06710100 | 1.63739800 | 5.77439700 |
| H | 3.08707100 | 1.32726300 | 6.16479400 |
| C | 5.95240500 | 3.20052200 | 14.80900800 |
| H | 6.88116000 | 3.12822900 | 15.37657500 |
| C | 5.54580200 | 8.13865400 | 9.48280700 |
| H | 5.52984900 | 7.07025100 | 9.74626800 |
| C | 3.95789000 | 2.14733500 | 13.95614200 |
| H | 3.33467200 | 1.25783400 | 13.85818400 |
| C | 6.54938400 | 5.60095300 | 14.20217800 |
| H | 5.94132900 | 6.50947800 | 14.32435500 |
| C | 5.13898000 | 2.07707800 | 14.68832400 |

| | | | |
|---|---|---|---|
| H | 5.43180000 | 1.13842500 | 15.16297200 |
| C | 0.67474600 | 7.89363000 | 10.92472400 |
| H | 1.02974600 | 7.93542600 | 11.96366900 |
| C | 2.15051600 | -0.29435900 | 8.55727500 |
| H | 2.62145500 | 0.67871400 | 8.35669900 |
| C | -3.55015800 | 2.48322700 | 8.96401500 |
| H | -3.78994700 | 2.51469400 | 10.03801700 |
| H | -3.98660600 | 1.56494300 | 8.54336000 |
| H | -4.04614300 | 3.34663300 | 8.49680700 |
| C | 6.17351600 | 8.91503500 | 10.64728300 |
| H | 6.18122800 | 9.99644500 | 10.43194700 |
| H | 7.21356100 | 8.58985700 | 10.80636000 |
| H | 5.61801700 | 8.75090200 | 11.58194200 |
| C | 2.02293300 | -1.04414100 | 7.22556500 |
| H | 3.01934300 | -1.21214100 | 6.78647700 |
| H | 1.42317200 | -0.47815700 | 6.49871200 |
| H | 1.54621500 | -2.02535800 | 7.38204500 |
| C | 1.18910600 | 4.14815400 | 13.45929700 |
| H | 1.02622900 | 3.59573700 | 14.39902300 |
| H | 0.22866600 | 4.20511600 | 12.92442700 |
| H | 1.50815800 | 5.17177500 | 13.70600800 |
| C | 1.70169200 | 2.08704700 | 12.10470100 |
| H | 2.47132100 | 1.54044300 | 11.53954000 |
| H | 0.84559900 | 2.24358100 | 11.42980800 |
| H | 1.35845300 | 1.45116300 | 12.93739900 |
| C | 1.53080000 | 6.35283900 | 7.24433500 |
| H | 2.39324800 | 6.98351900 | 7.51362000 |
| H | 1.59746100 | 5.43727300 | 7.84808800 |
| H | 0.61613500 | 6.90138300 | 7.51770500 |
| C | 1.39270700 | 7.34525600 | 4.94341400 |
| H | 0.41395900 | 7.80307600 | 5.14979000 |
| H | 1.47482500 | 7.17489100 | 3.85903300 |

| | | | |
|---|---|---|---|
| H | 2.15796900 | 8.07927500 | 5.24036700 |
| B | 3.32192000 | 3.20677000 | 9.47207800 |
| C | 3.08297900 | -1.04673100 | 9.50455500 |
| H | 2.74941600 | -2.08358600 | 9.67146900 |
| H | 3.15867200 | -0.54241300 | 10.47851500 |
| H | 4.09224500 | -1.08963600 | 9.07037100 |
| C | -0.65794400 | 8.63842300 | 10.87211200 |
| H | -0.53825000 | 9.71142200 | 11.08521100 |
| H | -1.35018700 | 8.21492300 | 11.61464900 |
| H | -1.13696800 | 8.53699600 | 9.88566600 |
| B | 3.99730300 | 5.93577100 | 11.35437400 |
| C | 6.39780200 | 8.28331500 | 8.22329500 |
| H | 5.93161600 | 7.78000100 | 7.36213600 |
| H | 7.38244700 | 7.82120400 | 8.38823600 |
| H | 6.56517600 | 9.34010200 | 7.95607800 |
| B | 1.53510600 | 2.79517800 | 7.60886400 |
| C | 0.46367300 | 6.41294700 | 10.58063000 |
| H | 0.00403200 | 6.31739600 | 9.58697100 |
| H | -0.20732900 | 5.93943200 | 11.31363300 |
| H | 1.41178600 | 5.85394000 | 10.56004700 |
| C | 7.56293500 | 5.56732600 | 15.34530900 |
| H | 8.30035200 | 4.76139500 | 15.20655800 |
| H | 7.07437900 | 5.41804100 | 16.31977900 |
| H | 8.12001500 | 6.51507700 | 15.37997500 |
| C | 7.27376500 | 5.72300100 | 12.85368800 |
| H | 7.96322500 | 6.58136200 | 12.86447200 |
| H | 6.56597700 | 5.86735300 | 12.02439500 |
| H | 7.85991200 | 4.81282500 | 12.64809900 |
| C | 4.34119700 | 0.79883100 | 4.52222500 |
| H | 4.34317600 | -0.27384000 | 4.77025400 |
| H | 3.57277200 | 0.97355200 | 3.75467700 |
| H | 5.32212400 | 1.04374800 | 4.08513300 |

| | | | |
|---|---|---|---|
| C | 5.10957400 | 1.36969600 | 6.86517800 |
| H | 4.88079000 | 1.92116000 | 7.78868000 |
| H | 5.14048400 | 0.29487900 | 7.10745400 |
| H | 6.11895900 | 1.67565500 | 6.54387100 |
| C | -1.44193100 | 3.79354100 | 9.40220100 |
| H | -0.35556100 | 3.85570000 | 9.23767600 |
| H | -1.90636500 | 4.71665900 | 9.02205500 |
| H | -1.61221200 | 3.74582100 | 10.49020000 |
| O | 4.37285100 | 2.82790000 | 10.18747400 |
| C | 5.20368800 | 4.03570500 | 10.42860400 |
| C | 6.34711600 | 3.94634300 | 9.42007100 |
| C | 6.31452300 | 4.68571300 | 8.23599800 |
| C | 7.42112000 | 3.08321400 | 9.65795500 |
| C | 7.35514700 | 4.57532000 | 7.31248600 |
| H | 5.45854400 | 5.34516900 | 8.06814300 |
| C | 8.45741200 | 2.96586000 | 8.73315000 |
| H | 7.43478700 | 2.49774400 | 10.58166100 |
| C | 8.42837900 | 3.71785100 | 7.55712100 |
| H | 7.32271200 | 5.16377100 | 6.39359400 |
| H | 9.29303400 | 2.29101500 | 8.93087600 |
| H | 9.24311900 | 3.63682900 | 6.83435900 |
| H | 5.63218600 | 3.84893800 | 11.43191200 |

**IN2**

| | | | |
|---|---|---|---|
| O | 12.03478100 | 10.01509200 | 10.30164100 |
| N | 12.48609000 | 7.55076300 | 10.91495300 |
| N | 10.35774800 | 6.35934600 | 11.80642400 |
| N | 12.36467900 | 5.30031000 | 12.14887400 |
| C | 8.05718400 | 9.24609100 | 11.46522600 |
| H | 7.89633700 | 10.23305400 | 11.90076300 |
| C | 9.30886900 | 7.18675400 | 11.34447800 |
| C | 7.21160600 | 8.79657500 | 10.45677400 |

| | | | |
|---|---|---|---|
| H | 6.38671000 | 9.42509600 | 10.11480100 |
| C | 13.72196200 | 4.93835500 | 12.29210800 |
| C | 8.49621000 | 6.74765600 | 10.27620800 |
| C | 9.11343700 | 8.45475100 | 11.92791900 |
| C | 7.43583500 | 7.55765000 | 9.86185800 |
| H | 6.78939200 | 7.22995300 | 9.04601800 |
| C | 15.65200100 | 4.57733700 | 13.70377600 |
| H | 16.13366800 | 4.61154200 | 14.68233100 |
| B | 11.80720300 | 6.53262100 | 11.54333600 |
| B | 12.22455200 | 8.82349300 | 10.58357300 |
| C | 14.32946000 | 5.00859300 | 13.56465900 |
| C | 10.14439400 | 5.16160300 | 12.48861500 |
| H | 9.14593000 | 4.83735100 | 12.77722300 |
| C | 16.37390600 | 4.12881700 | 12.60149700 |
| H | 17.40809200 | 3.79960700 | 12.72236900 |
| C | 8.87344700 | 5.48599600 | 9.51650900 |
| H | 9.24289800 | 4.74731400 | 10.24294100 |
| C | 11.32898600 | 4.54243400 | 12.70314600 |
| H | 11.52116600 | 3.59742100 | 13.20793800 |
| C | 14.45810400 | 4.52873200 | 11.16050800 |
| C | 10.00696300 | 8.91793700 | 13.06635800 |
| C | 10.18853400 | 10.43357100 | 13.11262400 |
| H | 9.25321700 | 10.95688600 | 13.37103800 |
| H | 10.56650400 | 10.79097300 | 12.14331200 |
| H | 10.92938200 | 10.69074900 | 13.88512300 |
| C | 15.78578500 | 4.12685400 | 11.33970500 |
| H | 16.37304300 | 3.80519400 | 10.47864700 |
| C | 12.79904400 | 3.36802000 | 9.66329100 |
| H | 12.32596600 | 3.37803800 | 8.66905200 |
| H | 12.00528500 | 3.43488400 | 10.42055400 |
| H | 13.31765400 | 2.40250600 | 9.78428000 |
| C | 13.79511200 | 4.52715000 | 9.79214200 |

| | | | |
|---|---|---|---|
| H | 13.24237900 | 5.47754500 | 9.72358400 |
| C | 14.79258900 | 4.49560000 | 8.63612800 |
| H | 15.32406700 | 3.53135700 | 8.57888300 |
| H | 15.53955000 | 5.29725600 | 8.72782400 |
| H | 14.26011600 | 4.63444700 | 7.68378600 |
| C | 13.62344600 | 7.19747600 | 14.50906800 |
| H | 14.65612200 | 7.56274800 | 14.62931500 |
| H | 12.98806900 | 7.70606800 | 15.25124300 |
| H | 13.28646000 | 7.47849000 | 13.49975000 |
| C | 13.58314800 | 5.67469700 | 14.70961500 |
| C | 14.10581400 | 5.29697900 | 16.09411900 |
| H | 14.13780400 | 4.20588100 | 16.23499400 |
| H | 13.45480200 | 5.72560600 | 16.87047800 |
| H | 15.11911300 | 5.69279200 | 16.26572300 |
| C | 10.03180200 | 5.81225100 | 8.56225900 |
| H | 10.38568800 | 4.90243000 | 8.05232400 |
| H | 10.87513500 | 6.27127700 | 9.10035400 |
| H | 9.69469900 | 6.53003400 | 7.79743000 |
| C | 7.71100700 | 4.84921700 | 8.75688400 |
| H | 6.84490400 | 4.67108400 | 9.41209600 |
| H | 8.02468500 | 3.88500500 | 8.32975200 |
| H | 7.37980900 | 5.48278800 | 7.91929300 |
| C | 9.49087700 | 8.35761300 | 14.39645800 |
| H | 10.15264700 | 8.65348900 | 15.22567200 |
| H | 9.44689400 | 7.25845100 | 14.36645100 |
| H | 8.47887300 | 8.73897200 | 14.61069500 |
| H | 12.52958200 | 5.36461700 | 14.65428700 |
| H | 11.00063300 | 8.48606600 | 12.88647700 |

**7**

| | | | |
|---|---|---|---|
| N | 3.16497200 | 6.04311000 | 5.63085300 |
| N | 3.40439400 | 6.16717000 | 3.36415700 |

| | | | |
|---|---|---|---|
| N | 4.28436600 | 8.18723000 | 4.77463000 |
| C | 2.93538600 | 6.38827800 | 6.99478700 |
| C | 2.61125100 | 4.90528100 | 5.04497400 |
| H | 2.14680200 | 4.12663500 | 5.64513900 |
| C | 2.75571300 | 4.97619200 | 3.69868700 |
| H | 2.44025800 | 4.26881700 | 2.93519600 |
| C | 3.62855500 | 6.57899300 | 2.02031800 |
| C | 5.13295900 | 8.68630500 | 3.97210600 |
| H | 5.52961800 | 8.12932200 | 3.09830900 |
| C | 1.81649700 | 7.18249900 | 7.30551000 |
| C | 2.88295900 | 7.66530700 | 1.50565500 |
| C | 5.04427400 | 5.11661800 | 7.60393200 |
| H | 4.77390600 | 4.51506200 | 6.72269100 |
| C | 3.81723500 | 5.92539400 | 7.98931600 |
| C | 4.60360600 | 5.92332000 | 1.24779400 |
| C | 1.81470200 | 8.34980800 | 2.34809600 |
| H | 2.20950900 | 8.45123600 | 3.36983000 |
| C | 5.66744400 | 10.05665700 | 4.11467700 |
| C | 6.50840500 | 10.57677000 | 3.12521700 |
| H | 6.76370700 | 9.95861900 | 2.26073200 |
| C | 5.45169400 | 4.79328100 | 1.80883000 |
| H | 5.18979700 | 4.66899900 | 2.86858500 |
| C | 3.13678800 | 8.06912700 | 0.19322900 |
| H | 2.58403700 | 8.90829400 | -0.22892600 |
| C | 1.60515700 | 7.52579900 | 8.64526000 |
| H | 0.74954900 | 8.15000900 | 8.91125000 |
| C | 4.80565100 | 6.35117800 | -0.07003900 |
| H | 5.55270600 | 5.84792900 | -0.68802800 |
| C | 0.87338200 | 7.69042500 | 6.22594800 |
| H | 1.09808600 | 7.14313500 | 5.29802100 |
| C | 5.33265700 | 10.84349900 | 5.22528500 |
| H | 4.68086500 | 10.41170400 | 5.98689000 |

| | | | |
|---|---|---|---|
| C | 3.56141500 | 6.28315700 | 9.31529400 |
| H | 4.22678100 | 5.93672200 | 10.10700900 |
| C | 5.83467900 | 12.13470600 | 5.33799700 |
| H | 5.57712100 | 12.74615600 | 6.20415900 |
| C | 4.08274500 | 7.41436000 | -0.59413500 |
| H | 4.25738400 | 7.74227000 | -1.62003000 |
| C | 6.67138100 | 12.65153200 | 4.34395000 |
| H | 7.06311400 | 13.66595200 | 4.43542300 |
| C | 7.00792200 | 11.87315000 | 3.23730600 |
| H | 7.66044400 | 12.27686900 | 2.46189100 |
| C | 2.46674100 | 7.08056000 | 9.64153200 |
| H | 2.28420300 | 7.35386200 | 10.68209900 |
| C | 0.54915100 | 7.48589700 | 2.42627000 |
| H | 0.10734500 | 7.36285000 | 1.42524400 |
| H | -0.20077000 | 7.96391600 | 3.07585100 |
| H | 0.76436600 | 6.48768400 | 2.83304000 |
| C | 1.11705700 | 9.17992200 | 5.95538700 |
| H | 0.88893700 | 9.77883500 | 6.85142200 |
| H | 0.46939400 | 9.53411800 | 5.13763700 |
| H | 2.16595300 | 9.35871000 | 5.67711900 |
| C | -0.59448900 | 7.42199100 | 6.56614200 |
| H | -0.76767400 | 6.35638400 | 6.77382700 |
| H | -1.23882600 | 7.71808000 | 5.72488100 |
| H | -0.91851900 | 7.99760600 | 7.44643700 |
| C | 5.51161200 | 4.15916300 | 8.69911200 |
| H | 5.92455100 | 4.70271600 | 9.56232600 |
| H | 6.31073300 | 3.50951200 | 8.31417100 |
| H | 4.69115400 | 3.52099700 | 9.05779600 |
| C | 6.94435000 | 5.13203700 | 1.74154300 |
| H | 7.28557800 | 5.23712900 | 0.70045200 |
| H | 7.54002000 | 4.33439400 | 2.20931000 |
| H | 7.16256300 | 6.07378600 | 2.26565400 |

| | | | |
|---|---|---|---|
| C | 6.17954400 | 6.06166300 | 7.18941700 |
| H | 5.86241800 | 6.73662100 | 6.38216500 |
| H | 7.05665800 | 5.49159100 | 6.84771500 |
| H | 6.48452800 | 6.68345000 | 8.04531300 |
| C | 1.47257600 | 9.75913300 | 1.86900500 |
| H | 2.37379100 | 10.38099100 | 1.76619200 |
| H | 0.80491900 | 10.24495700 | 2.59482100 |
| H | 0.94791500 | 9.74556000 | 0.90134600 |
| B | 3.69186100 | 6.87857300 | 4.58692100 |
| C | 5.15674100 | 3.47137600 | 1.09315600 |
| H | 4.09271300 | 3.20503600 | 1.16801900 |
| H | 5.74819800 | 2.65343700 | 1.53027200 |
| H | 5.41053500 | 3.53778600 | 0.02383000 |

**TS3**

| | | | |
|---|---|---|---|
| O | 11.34381400 | 9.49350400 | 9.61756700 |
| O | 14.29141900 | 9.30411400 | 10.64231400 |
| N | 12.58194000 | 7.60594700 | 10.92222700 |
| N | 10.49384100 | 6.08345800 | 11.00272600 |
| C | 14.11699000 | 8.29250300 | 11.37457800 |
| N | 12.38879100 | 5.21301200 | 11.95619800 |
| C | 13.96088200 | 8.57016000 | 12.87288700 |
| C | 7.93867200 | 8.64907700 | 10.19342900 |
| H | 7.49638500 | 9.54583500 | 10.62892200 |
| C | 9.50443000 | 6.82355200 | 10.29922200 |
| C | 13.05680900 | 9.55018300 | 13.30234900 |
| H | 12.42428500 | 10.03590800 | 12.55323800 |
| C | 7.50579500 | 8.20830600 | 8.94848600 |
| H | 6.71815800 | 8.75010400 | 8.42115200 |
| C | 13.63017800 | 4.97906500 | 12.59761100 |
| C | 9.11695700 | 6.39760300 | 9.01161100 |
| C | 8.94255600 | 7.97048000 | 10.88914800 |

| | | | |
|---|---|---|---|
| C | 8.09884800 | 7.09605100 | 8.35794100 |
| H | 7.77617400 | 6.78166500 | 7.36450300 |
| C | 14.93521700 | 4.61293500 | 14.58830400 |
| H | 15.02103400 | 4.52471200 | 15.67045200 |
| C | 12.98940000 | 9.91073500 | 14.64459300 |
| H | 12.27555900 | 10.67134900 | 14.96999100 |
| B | 11.89792600 | 6.41063100 | 11.26451900 |
| B | 12.00620600 | 8.64812900 | 10.19985800 |
| C | 13.68550700 | 4.85711900 | 14.00621900 |
| C | 10.22400300 | 4.80695800 | 11.50080900 |
| H | 9.24014000 | 4.35529400 | 11.39324000 |
| C | 16.08744800 | 4.50337900 | 13.81613000 |
| H | 17.05071700 | 4.32668900 | 14.29861700 |
| C | 14.80896700 | 7.97915200 | 13.80675700 |
| H | 15.51971400 | 7.21809300 | 13.47168800 |
| C | 9.88417400 | 5.28374900 | 8.31543100 |
| H | 10.12579100 | 4.51436800 | 9.06373100 |
| C | 11.33806200 | 4.29546900 | 12.06630500 |
| H | 11.48443700 | 3.32116100 | 12.52567500 |
| C | 14.78822100 | 4.85489300 | 11.79980100 |
| C | 13.84535300 | 9.31273900 | 15.57850900 |
| H | 13.79775200 | 9.60201300 | 16.63059500 |
| C | 9.41130500 | 8.44960200 | 12.25256900 |
| C | 9.35102700 | 9.96877100 | 12.40154200 |
| H | 8.31470800 | 10.34102100 | 12.44600900 |
| H | 9.87106900 | 10.44458200 | 11.55689200 |
| H | 9.84689300 | 10.26718100 | 13.33786500 |
| C | 16.01337700 | 4.62644600 | 12.43276200 |
| H | 16.92248200 | 4.54210700 | 11.83688100 |
| C | 14.75740100 | 8.34875900 | 15.15504500 |
| H | 15.43163900 | 7.87575200 | 15.87363800 |
| C | 14.08149900 | 3.60924300 | 9.74129700 |

| | | | |
|---|---|---|---|
| H | 13.97753800 | 3.66050200 | 8.64651300 |
| H | 13.08768800 | 3.41781000 | 10.17041300 |
| H | 14.73619100 | 2.75680900 | 9.98480700 |
| C | 14.68020600 | 4.91421200 | 10.28230000 |
| H | 13.98893200 | 5.73270300 | 10.03449800 |
| C | 16.00416800 | 5.22479900 | 9.58806300 |
| H | 16.72551800 | 4.39818800 | 9.69193200 |
| H | 16.45948600 | 6.14215100 | 9.98880200 |
| H | 15.83007200 | 5.37737900 | 8.51329200 |
| C | 11.71809100 | 6.34636400 | 14.72290700 |
| H | 12.39771200 | 7.17936300 | 14.94940700 |
| H | 10.87158100 | 6.38925400 | 15.42562300 |
| H | 11.31525900 | 6.49650900 | 13.71397200 |
| C | 12.43298600 | 4.99522200 | 14.87541600 |
| C | 12.70548500 | 4.74183600 | 16.35860900 |
| H | 13.17090100 | 3.76166500 | 16.53822900 |
| H | 11.75863700 | 4.77518200 | 16.91632300 |
| H | 13.36349200 | 5.52079300 | 16.77508300 |
| C | 11.21202500 | 5.84083500 | 7.78285300 |
| H | 11.80581900 | 5.04436100 | 7.30762400 |
| H | 11.81117100 | 6.28968200 | 8.58800300 |
| H | 11.01999000 | 6.62830900 | 7.03802200 |
| C | 9.10064400 | 4.60487700 | 7.19304500 |
| H | 8.11337200 | 4.26037100 | 7.53565000 |
| H | 9.65956400 | 3.73436700 | 6.81897100 |
| H | 8.94806400 | 5.28398600 | 6.34009100 |
| C | 8.63609200 | 7.73556300 | 13.36473200 |
| H | 9.00190200 | 8.05112900 | 14.35414400 |
| H | 8.75462300 | 6.64391200 | 13.28826800 |
| H | 7.56162600 | 7.97232400 | 13.29786700 |
| H | 14.77451100 | 7.39277700 | 11.24588500 |
| H | 10.46992100 | 8.17107700 | 12.35374400 |

| | | | |
|---|---|---|---|
| H | 11.72304100 | 4.22033200 | 14.54487300 |

**IN3**

| | | | |
|---|---|---|---|
| O | 11.28738700 | 9.51740000 | 9.80386600 |
| O | 13.64116200 | 9.45523000 | 10.69655200 |
| N | 12.57552700 | 7.69287300 | 11.14509000 |
| N | 10.56510500 | 6.11827300 | 11.05743200 |
| C | 13.85841700 | 8.29631200 | 11.46176400 |
| N | 12.42844300 | 5.28111600 | 12.11208400 |
| C | 14.06119500 | 8.64213600 | 12.92874300 |
| C | 7.97432000 | 8.58017100 | 10.06367100 |
| H | 7.47487500 | 9.46550200 | 10.45852200 |
| C | 9.60335600 | 6.82592600 | 10.28226900 |
| C | 13.21440400 | 9.58776200 | 13.52183700 |
| H | 12.42990500 | 10.04221800 | 12.91095100 |
| C | 7.62520800 | 8.10261600 | 8.80565800 |
| H | 6.84117200 | 8.60022600 | 8.23101200 |
| C | 13.69453200 | 5.05728400 | 12.69909600 |
| C | 9.31752100 | 6.37744000 | 8.97788900 |
| C | 8.97021800 | 7.95885500 | 10.82000600 |
| C | 8.30513400 | 7.01832300 | 8.25908300 |
| H | 8.05946900 | 6.68365900 | 7.25004600 |
| C | 15.07612900 | 4.70089300 | 14.64446600 |
| H | 15.19383300 | 4.60476200 | 15.72393700 |
| C | 13.39066000 | 9.95403100 | 14.85207000 |
| H | 12.72760300 | 10.69469300 | 15.30404800 |
| B | 11.93472100 | 6.47400900 | 11.41753100 |
| B | 12.28361900 | 9.00481800 | 10.37983800 |
| C | 13.80764600 | 4.93123000 | 14.10250600 |
| C | 10.30590900 | 4.82894900 | 11.52524400 |
| H | 9.33949300 | 4.35683200 | 11.36050100 |
| C | 16.20206000 | 4.60995800 | 13.83091000 |

| | | | |
|---|---|---|---|
| H | 17.18404400 | 4.43852600 | 14.27627000 |
| C | 15.08143000 | 8.07400800 | 13.68818000 |
| H | 15.73971800 | 7.33120700 | 13.23014200 |
| C | 10.21009900 | 5.33237900 | 8.32712700 |
| H | 10.47179700 | 4.57872000 | 9.08499200 |
| C | 11.39472700 | 4.33345700 | 12.15370100 |
| H | 11.53612600 | 3.35673900 | 12.61042100 |
| C | 14.82763900 | 4.96018600 | 11.86060500 |
| C | 14.41993500 | 9.38322000 | 15.60949600 |
| H | 14.55967300 | 9.67474200 | 16.65261700 |
| C | 9.36812200 | 8.48252700 | 12.18790000 |
| C | 9.20368000 | 9.99549000 | 12.30897900 |
| H | 8.14452000 | 10.30180900 | 12.30909000 |
| H | 9.73306100 | 10.47396500 | 11.47151300 |
| H | 9.64084200 | 10.34188000 | 13.25895800 |
| C | 16.07692900 | 4.74247100 | 12.45142700 |
| H | 16.96640900 | 4.67076600 | 11.82467600 |
| C | 15.26200100 | 8.44012700 | 15.02558600 |
| H | 16.06430800 | 7.98134600 | 15.60791700 |
| C | 14.04855000 | 3.73159600 | 9.81543000 |
| H | 13.92561400 | 3.78647200 | 8.72301400 |
| H | 13.06070800 | 3.54790400 | 10.26080800 |
| H | 14.70134200 | 2.87402800 | 10.04579400 |
| C | 14.66886600 | 5.03012000 | 10.34805800 |
| H | 13.96731300 | 5.84824000 | 10.12769400 |
| C | 15.97020200 | 5.33742200 | 9.60999800 |
| H | 16.68304100 | 4.49957300 | 9.67391600 |
| H | 16.45978300 | 6.23852700 | 10.00822100 |
| H | 15.75852300 | 5.51024200 | 8.54508000 |
| C | 12.06437400 | 6.53718700 | 14.99149900 |
| H | 12.82640300 | 7.22040700 | 15.39435400 |
| H | 11.16080100 | 6.61372800 | 15.61588100 |

| | | | |
|---|---|---|---|
| H | 11.80762000 | 6.88010000 | 13.98008000 |
| C | 12.58173300 | 5.09246400 | 14.99469000 |
| C | 12.81490100 | 4.63073700 | 16.43236200 |
| H | 13.20573100 | 3.60319100 | 16.47972900 |
| H | 11.86778800 | 4.66414100 | 16.99009300 |
| H | 13.52435700 | 5.29366200 | 16.95206300 |
| C | 11.51181200 | 6.01561800 | 7.88253600 |
| H | 12.22091100 | 5.28061700 | 7.46864100 |
| H | 11.98762200 | 6.54609200 | 8.72030100 |
| H | 11.29439700 | 6.76940000 | 7.11031400 |
| C | 9.55777300 | 4.60000800 | 7.15596100 |
| H | 8.58728400 | 4.16445000 | 7.43799900 |
| H | 10.21308200 | 3.78749900 | 6.80773200 |
| H | 9.39312100 | 5.27395200 | 6.30112600 |
| C | 8.62466700 | 7.72810600 | 13.29515400 |
| H | 8.94506900 | 8.08109800 | 14.28828400 |
| H | 8.82223900 | 6.64667600 | 13.23622900 |
| H | 7.53685000 | 7.88468700 | 13.20788100 |
| H | 14.73731400 | 7.71370700 | 11.11176200 |
| H | 10.44190900 | 8.28122200 | 12.30031300 |
| H | 11.78334300 | 4.46310900 | 14.57378000 |

**TS4**

| | | | |
|---|---|---|---|
| O | 11.23153600 | 10.52323500 | 10.53472800 |
| O | 13.60183800 | 9.68715400 | 10.48560600 |
| N | 12.75685500 | 7.27301600 | 11.17952900 |
| N | 10.61701900 | 5.85407300 | 11.11491000 |
| C | 13.77659500 | 8.02004900 | 11.41062800 |
| N | 12.44250200 | 4.95297200 | 12.19413700 |
| C | 14.00202400 | 8.56567500 | 12.79825400 |
| C | 8.26998800 | 8.58593900 | 10.23223500 |
| H | 7.86248000 | 9.50072500 | 10.66137600 |

| | | | |
|---|---|---|---|
| C | 9.70456400 | 6.65669700 | 10.38404100 |
| C | 13.07955400 | 9.41736300 | 13.41907900 |
| H | 12.20664100 | 9.76659300 | 12.86004800 |
| C | 7.91000000 | 8.21761000 | 8.94049900 |
| H | 7.20844900 | 8.83385500 | 8.37459600 |
| C | 13.68595400 | 4.76894700 | 12.83675700 |
| C | 9.38853200 | 6.30133800 | 9.05581600 |
| C | 9.15873400 | 7.81065400 | 10.98277200 |
| C | 8.46668400 | 7.08372700 | 8.35562800 |
| H | 8.19971800 | 6.82278800 | 7.33029600 |
| C | 15.00643300 | 4.58046500 | 14.85262300 |
| H | 15.08070000 | 4.55073400 | 15.94022800 |
| C | 13.28556300 | 9.83031200 | 14.73229500 |
| H | 12.56814400 | 10.50586400 | 15.20276300 |
| B | 12.00254600 | 6.16798500 | 11.47600600 |
| B | 12.39571700 | 10.10938600 | 10.47488700 |
| C | 13.75449500 | 4.72836900 | 14.24765800 |
| C | 10.31324900 | 4.57091300 | 11.57713800 |
| H | 9.33318000 | 4.12758300 | 11.41169600 |
| C | 16.16721500 | 4.49638800 | 14.09014300 |
| H | 17.13708900 | 4.39220100 | 14.58073200 |
| C | 15.11400200 | 8.12271600 | 13.51598700 |
| H | 15.82074600 | 7.43734500 | 13.03933400 |
| C | 10.14585200 | 5.16978500 | 8.37818600 |
| H | 10.29754800 | 4.36973500 | 9.11772000 |
| C | 11.37838300 | 4.04426800 | 12.22546800 |
| H | 11.47275600 | 3.06581000 | 12.69202100 |
| C | 14.85916200 | 4.68269500 | 12.05125500 |
| C | 14.40215400 | 9.38687600 | 15.44800000 |
| H | 14.55674300 | 9.71054700 | 16.47937400 |
| C | 9.52136900 | 8.17629800 | 12.41402700 |
| C | 9.19016600 | 9.62136500 | 12.77944600 |

| | | | |
|---|---|---|---|
| H | 8.10053000 | 9.78817000 | 12.82156100 |
| H | 9.64844400 | 10.31036500 | 12.05606400 |
| H | 9.59088700 | 9.84228400 | 13.78161800 |
| C | 16.09080600 | 4.55244600 | 12.70177400 |
| H | 17.00773000 | 4.49054000 | 12.11467900 |
| C | 15.31143800 | 8.52463900 | 14.83933700 |
| H | 16.17879300 | 8.15770900 | 15.39227700 |
| C | 14.17516600 | 3.36421700 | 10.02549900 |
| H | 14.08767400 | 3.37957500 | 8.92812300 |
| H | 13.17514700 | 3.18576500 | 10.44442500 |
| H | 14.82838100 | 2.52196500 | 10.30640100 |
| C | 14.76386300 | 4.68710500 | 10.53246600 |
| H | 14.06700600 | 5.49292200 | 10.25820200 |
| C | 16.09207700 | 4.97509700 | 9.83546600 |
| H | 16.81155800 | 4.15094900 | 9.96898400 |
| H | 16.55465000 | 5.90022400 | 10.21002100 |
| H | 15.92447900 | 5.09458600 | 8.75529600 |
| C | 12.10533200 | 6.42906100 | 15.05072900 |
| H | 12.92006000 | 7.05226000 | 15.45106200 |
| H | 11.20138500 | 6.59987500 | 15.65574000 |
| H | 11.90138500 | 6.76887900 | 14.02531000 |
| C | 12.50140200 | 4.94766400 | 15.08296900 |
| C | 12.63745600 | 4.46748300 | 16.52726400 |
| H | 12.98868800 | 3.42598700 | 16.58352100 |
| H | 11.66307800 | 4.53067400 | 17.03340000 |
| H | 13.33978400 | 5.09742500 | 17.09498900 |
| C | 11.53365100 | 5.67610000 | 7.95884000 |
| H | 12.14174400 | 4.85512000 | 7.54651800 |
| H | 12.06800100 | 6.12539700 | 8.80906500 |
| H | 11.43007900 | 6.45372400 | 7.18563500 |
| C | 9.41120600 | 4.56191900 | 7.18456700 |
| H | 8.38934100 | 4.25195900 | 7.45033200 |

| | | | |
|---|---|---|---|
| H | 9.95565400 | 3.67907800 | 6.81736900 |
| H | 9.34403300 | 5.27361500 | 6.34718000 |
| C | 8.86248100 | 7.21089500 | 13.40807000 |
| H | 9.12805300 | 7.48972300 | 14.43968800 |
| H | 9.17823200 | 6.17078400 | 13.24505900 |
| H | 7.76511400 | 7.26130700 | 13.31388400 |
| H | 14.71903000 | 7.87051000 | 10.85124400 |
| H | 10.61463100 | 8.06456800 | 12.49086600 |
| H | 11.68139400 | 4.37871400 | 14.62116500 |

**$BO_2^-$**

| | | | |
|---|---|---|---|
| B | -2.05658400 | 1.17410800 | -0.41303100 |
| O | -1.14070900 | 0.31678300 | -0.47463400 |
| O | -2.97205700 | 2.03194900 | -0.35258700 |

## 6. References for supporting information


[S1] J. Tang, C. Hu, A. E. Crumpton, D. Sarkar, L. P. Griffin, J. M. Goicoechea and S. Aldridge, *J. Am. Chem. Soc.*, 2024, **146**, 30778-30783.

[S2] L. Liu, D. A. Ruiz, D. Munz and G. Bertrand, *Chem.*, 2016, **1**, 147–153.

[S3] J. Cosier and A. M. Glazer, *A Nitrogen-Gas-Stream Cryostat for General X-Ray Diffraction Studies*, (1986).

[S4] *CrysAlisPro, Agilent Technologies, Version 1.171.42.57a*.

[S5] G. M. Sheldrick, *Acta Cryst. A*, 2015, **71**, 3–8.

[S6] G. M. Sheldrick, *Acta Cryst. C,* 2015, **71**, 3–8.

[S7] O. V. Dolomanov, L. J. Bourhis, R. J. Gildea, J. A. K. Howard and H. Puschmann, *J. Appl. Cryst.*, 2009, **42**, 339–341.

[S8] M. J. Frisch, G. W. Trucks, H. B. Schlegel, G. E. Scuseria, M. A. Robb, J. R. Cheeseman, G. Scalmani, V. Barone, G. A. Petersson, H. Nakatsuji, X. Li, M. Caricato, A. V. Marenich, J. Bloino, B. G. Janesko, R. Gomperts, B. Mennucci, H. P. Hratchian, J. V. Ortiz, A. F. Izmaylov, J. L. Sonnenberg, D. Williams-Young, F. Ding, F. Lipparini, F. Egidi, J. Goings, B. Peng, A. Petrone, T. Henderson, D. Ranasinghe, V. G. Zakrzewski, J. Gao, N. Rega, G. Zheng, W. Liang, M. Hada, M. Ehara, K. Toyota, R. Fukuda, J. Hasegawa, M. Ishida, T. Nakajima, Y. Honda, O. Kitao, H. Nakai, T., Vreven, K. Throssell, J. A. Montgomery Jr., J. E. Peralta, F. Ogliaro, M. J. Bearpark, J. J. Heyd, E. N. Brothers, K. N. Kudin, V. N. Staroverov, T. A. Keith, R. Kobayashi, J. Normand, K. Raghavachari, A. P. Rendell, J. C. Burant, S. S. Iyengar, J. Tomasi, M. Cossi, J. M. Millam, M. Klene, C. Adamo, R. Cammi, J. W. Ochterski, R. L. Martin, K. Morokuma, O. Farkas, J. B. Foresman and D. J. Fox, Gaussian 16, Revision B.01, Wallingford, CT, (2016).

[S9] Y. Zhao and D. G. Truhlar, *Theor. Chem. Acc.*, 2008, **120**, 215−241.

[S10] F. Weigend and R. Ahlrichs, *Phys. Chem. Chem. Phys.*, 2005, **7**, 3297−3305.

[S11] F. Weigend, *Phys. Chem. Chem. Phys.*, 2006, **8**, 1057−1065.

[S12] A. Schäfer, H. Horn and R. Ahlrichs, *J. Chem. Phys.*, 1992, **97**, 2571−2577.

[S13] A. Schäfer, C. Huber and R. Ahlrichs, *J. Chem. Phys.*, 1994, **100**, 5829−5835.

[S14] A. V. Marenich, C. J. Cramer and D. G. Truhlar, *J. Phys. Chem. B*, 2009, **113**, 6378−6396.

[S15] A. Bondi, *J. Phys. Chem.*, 1964, **68**, 441–451.
